# *In vitro* modeling of isoniazid resistance mechanisms in *Mycobacterium tuberculosis* H37Rv

**DOI:** 10.3389/fmicb.2023.1171861

**Published:** 2023-07-10

**Authors:** Thanadon Dokrungkoon, Orawan Tulyaprawat, Kamol Suwannakarn, Popchai Ngamskulrungroj

**Affiliations:** Department of Microbiology, Faculty of Medicine Siriraj Hospital, Mahidol University, Bangkoknoi, Bangkok, Thailand

**Keywords:** isoniazid (INH), *Mycobacterium tuberculosis*, resistance-associated variant (RAV), *katG*, *inhA*, *in vitro* model

## Abstract

**Introduction:**

*Mycobacterium tuberculosis* (MTB), the causative agent of tuberculosis, has been a global threat to human beings for several decades. Treating tuberculosis has become more difficult as the prevalence of drug-resistant tuberculosis has increased globally. Evidence suggests that the comprehensive landscape of resistance mechanisms in MTB is ambiguous. More importantly, little is known regarding the series of events connected to resistance mechanisms in MTB before exposure to anti-TB drugs, during exposure to the drugs, and finally, when the MTB becomes resistant after exposure, upon analyses of its genome.

**Methods:**

We used the wild-type strain of MTB (H37Rv) in an *in vitro* model for generating induced resistance using a sub-inhibitory concentration of isoniazid, and the generated resistance-associated variants (RAVs) were identified using the whole genome sequencing method.

**Results:**

The detection of an *inhA* promoter mutation (*fabG1*−15C>T), which results in increased production of InhA protein, was found to be a major mechanism for developing resistance to isoniazid in the first place. We observed adaptation of MTB resistance mechanisms in high isoniazid stress by alteration and abolishment of KatG due to the detection of *katG* S315N, the common region of mutation that confers isoniazid resistance, along with *katG* K414N, *katG* N138S, and *katG* A162E. Furthermore, we detected the *ahpC*−72C>T and *ahpC* 21C>A mutations, but further investigation is needed to determine their role in compensating for the loss of KatG activity.

**Discussion:**

This suggests that increased InhA production is the main mechanism where there are low levels of isoniazid, whereas the alteration of KatG was found to be utilized in mycobacterium with a high concentration of isoniazid. Our work demonstrates that this *in vitro* approach of generating induced resistance could provide clinically relevant information after the *fabG1*−15C>T mutation, which is the common mutation found in clinical isolates. Moreover, other mutations detected in this work can also be found in clinical isolates. These findings may shed light on the impact of isoniazid in generating RAV and the resistance mechanism scenario that mycobacterium used under various isoniazid-pressuring conditions. More research is needed to understand better the role of RAV and mechanical resistance events within the mycobacterium genome in promoting a promising drug prediction platform that could lead to the right treatment for patients with MDR-TB and XDR-TB.

## 1. Introduction

Tuberculosis (TB), one of the main infectious airborne diseases caused by *Mycobacterium tuberculosis* (MTB), remains a global health problem that caused approximately 10.6 million cases and an estimated 1.6 million deaths worldwide in 2021 (World Health Organization, 2022). There were 1.4 million deaths among HIV-negative individuals and 187,000 among HIV-positive patients. The recommended therapeutic course for new patients with drug-susceptible TB is a 6-month regimen using a combination of four first-line anti-TB drugs (isoniazid, rifampicin, ethambutol, and pyrazinamide) (World Health Organization, 2022). However, in the past decade, treatment has become more difficult due to the increasing global multidrug resistance of tuberculosis. According to the World Health Organization (WHO), the estimated global number of patients enrolled in treatment for multidrug-resistant TB (MDR-TB; resistant to isoniazid and rifampin) and extensively drug-resistant TB (XDR-TB; resistant to rifampicin, plus any fluoroquinolone, plus at least one of the drugs bedaquiline and linezolid) in 2021 were 141,953 and 25,038, respectively (World Health Organization, 2022).

MTB is characterized by a low mutation rate (Ford et al., 2011), with an approximate evolutionary rate of 0.4–0.5 single nucleotide polymorphisms (SNP)/genome/year (Walker et al., 2013). Despite this low mutation rate, drug resistance has increased worldwide, especially in MDR and XDR cases, due to the acquisition of multiple unfixed drug resistance mutations and selective clonal sweeps of MTB populations in patients (Sun et al., 2012). The primary mechanism for acquiring drug resistance in MTB is through chromosomal mutations. The genes associated with resistance to first-line drugs are well-characterized, including *rpoB* mutations for rifampin resistance, *katG* and *inhA* mutations for isoniazid resistance, *embB* mutations for ethambutol resistance, and *pncA* mutations for pyrazinamide resistance (Coll et al., 2015). Since drug targets are generally involved in important biological functions, mutations in the genes involved should exhibit a biological cost, resulting in reduced fitness of resistant strains; for example, mutations in *kat*G lead to reduced protection against oxidative damage. The MTB genome is dynamically complex, and mutations associated with a high level of drug resistance and low or no fitness cost have been reported; for example, the *kat*G S315T, *rpo*B S531L, and *inh*A promoter mutation (Müller et al., 2013). However, the proportion of fitness cost depends on the mutation, the genetic background of the strain, and also the compensatory mutation, which is the process that alleviates loss of fitness due to drug resistance-associated mutations (Li et al., 2016; Nguyen et al., 2018).

The effects of drugs that affect the MTB genome in each generation remain unclear; some mutation-related drug resistance mechanisms and several fitness compensatory evolution mechanisms have not been widely investigated in MTB. Furthermore, resistance mechanisms and mechanical resistance events are not fully known (Oppong et al., 2019). Due to this ignorance, more work is needed to understand the complex factors driving drug resistance in MTB to control the rapid spread of highly drug-resistant strains, especially MDR-TB and XDR-TB strains.

Recently, whole genome sequencing (WGS) of MTB has been applied more frequently, and data retrieved from WGS can be used in many aspects (Galagan, 2014). In this study, the fully susceptible strain H37Rv (reference strain) was used to generate induced mutants with the first-line drug until it became a drug-resistant strain. Subsequently, WGS was used to determine the genome variation in MTB in each period of time (passage), and the agar proportion method was used as a phenotypic characterization method to eventually confirm resistance-associated variants (RAV). This approach was adopted to gain information on RAVs, mutation patterns or signatures, and potential details of evolutionary processes in the MTB genome.

Together, essential data on RAVs and promising details of the evolutionary processes of the MTB genome after exposure to isoniazid helped develop a precise model to predict the evolution of drug resistance and promote successful therapeutic strategies that can provide appropriate treatment to MDR and XDR-TB patients achieve better outcomes.

## 2. Materials and methods

All mycobacterial cultures and some DNA extraction parts were performed at the BSL-3 facility in the Microbiology Building, Department of Microbiology, Siriraj Hospital Faculty of Medicine. Biosafety approval was obtained from the Mahidol University Biosafety Committee (MU-IBC 2021-034).

### 2.1. Antimicrobial preparation

Isoniazid (Sigma-Aldrich Co., St Louis, MO, USA) was formulated in sterile distilled water according to the Clinical and Laboratory Standards Institute (CLSI) M24-A2 vol.31. Isoniazid was prepared at stock concentrations of 10 mg/ml and sterilized using sterile syringe filter with a 0.2 μm PTFE membrane (Merck, Kenilworth, NJ, USA) and finally maintained at−80°C. The stock solutions were continuously diluted with sterile distilled water to obtain the working concentrations, aliquoted in 2 ml vial tubes, and stored at−20°C until further testing.

### 2.2. Mycobacterial strain and culture condition

The *M. tuberculosis* American Type Culture Collection (ATCC) reference strain with a complete susceptibility profile was used to assess the impact of isoniazid on the genome and contribute to the type of RAV generated. In this study, the fully susceptible strain of *M. tuberculosis* H37Rv, ATCC27294, was collected in the mycobacterial laboratory of the Department of Microbiology of the Faculty of Medicine of Siriraj Hospital and used as a mycobacterial model. A multidrug-resistant strain derived from the faculty (resistant to isoniazid, rifampicin, ethambutol, and pyrazinamide) was used as a resistant control. Mycobacteria were grown on Löwenstein-Jensen (LJ) medium and incubated at 37°C for 3–4 weeks until further use in the drug resistance generation step.

Before initiating the generation of induced resistance, to ensure that H37Rv was fully susceptible to the first-line anti-TB drug, phenotypic susceptibility tests were carried out in triplicate using the agar proportion method on Middlebrook 7H10 agar (BD Difco, Sparks, MD, USA) supplemented with 10% v/v OADC (oleic acid, albumin, dextrose, catalase) and the BACTEC MGIT 960 system for pyrazinamide (World Health Organization, 2018). The drugs were used at the following critical concentrations: isoniazid at 0.2 μg/ml, rifampicin at 1.0 μg/ml, ethambutol at 5.0 μg/ml, and pyrazinamide at 100 μg/ml.

### 2.3. Generation of induced resistance in H37Rv

The MTB H37Rv cell stock was recovered and grown on LJ medium and then used to prepare the mycobacterial suspension and adjust the turbidity to the McFarland 1.0 standard. The generation of the induced resistance step was adapted and carried out following the Ismail et al. protocol, as previously described (Ismail et al., 2018). Briefly, several loops full of H37Rv mycobacterial colonies were suspended in phosphate-buffered saline (PBS) pH 7.4 containing six to seven 5 mm glass beads. The mycobacterial suspension was then vortexed for 10 m and allowed to settle for 15 m. Subsequently, the suspension was adjusted with PBS to create the standard 1.0 McFarland turbidity. Approximately 100 μl of each mycobacterial suspension was then inoculated on Middlebrook 7H10 agar supplemented with 10% OADC containing 0.5x the suggested critical concentration (CC) for solid media of isoniazid (0.1 μg/ml) together with the drug-free control plate. All culture plates were incubated at 37°C for 4 weeks. For the next passage, the suspension of each culture was prepared as mentioned above and inoculated onto the drug-free plate, the plate with growth allowing for drug concentration (same concentration as colonies were scraped), and the plate with two times higher drug concentration than the previous passage. This procedure was repeated until 6 passages or until it reached at least 4x the proposed CC. Sub-inhibitory concentrations (0.5xCC) were used to increase the growth of mycobacteria and build resistance to accumulation through each passage. This concentration allowed bacteria to adapt to the isoniazid environment while maintaining their growth, which subsequently led to the accumulation of resistance-associated mutations.

### 2.4. Phenotypic DST checking the resistance status

The susceptibility test for the MTB H37Rv drug was performed using the agar proportion method. This method was carried out in parallel with the generation of the induced resistance step to check the resistance status in each passage of induced MTB. The agar proportion was performed on Middlebrook 7H10 agar using the same drug concentrations used in the induced resistance step. In summary, a four quadrant plate of Middlebrook 7H10 agar was used, with the first quadrant of the plate containing drug-free or proportion control and the other quadrants containing the drug concentrations of isoniazid. The mycobacterial suspensions were adjusted to turbidity using PBS pH 7.4 until the 1.0 McFarland standard was reached, and then 0.1 ml of the suspension was transferred to a tube containing 9.9 ml of diluent and properly mixed. The resulting suspension was a 10^−2^ dilution of the original sample. Another 0.1 ml of 10^−2^ suspension was transferred to a fresh tube containing 9.9 ml to make a 10^−4^ dilution. Each quadrant was inoculated with a standard 0.1 ml of 10^−2^ and 10^−4^ mycobacterial diluted suspension. The plates were incubated at 37°C and examined at 21 and 28 days of incubation. Cultures were identified as resistant when colonies in the drug-containing quadrant were more than 1% compared to those in the drug-free control quadrant (World Health Organization, 2018).

### 2.5. Genomic DNA extraction of induced resistant cultures

The induction of mycobacterial cultures with isoniazid in each passage was collected as a stock. The induced mycobacterial stocks were seeded on Middlebrook 7H10 agar containing the drug with the same concentration as the induced stocks and incubated at 37°C for 4 weeks. Induced resistant cultures were extracted using genomic DNA to identify the evolution of the RAV and the H37Rv genome. Genomic DNA extraction was performed using the cetyl-trimethyl-ammonium bromide (Sigma-Aldrich Co., St Louis, MO, USA)-sodium chloride (CTAB/NaCl) and lysozyme method (Larsen et al., 2007). Mycobacterial cultures were harvested in a 15 ml tube containing TE buffer and 6–7 5 mm glass beads and then mixed in a vortex. After vigorous mixing, the suspensions were heated to 80°C for 20 m and incubated with 50 μl of 10 mg/ml lysozyme (VWR Life Science, Solon, OH, USA) overnight. Subsequently, the mixture was then added to 70 μl of 10% SDS and 5 μl of 20 mg/ml proteinase K (VWR Life Science, Solon, OH, USA) and incubated at 65°C for 20 m. Subsequently, 100 μl of 5 M NaCl and 100 μl of CTAB/NaCl were added. The mixture was incubated at 65°C for 10 m before adding 750 μl of 24:1 chloroform:isoamyl alcohol and centrifuged at 10,000 rpm for 5 m. After centrifugation, the supernatant was transferred to a new 1.5 ml microcentrifuge tube. The RNA was removed using 10 μl of 10 mg/ml of RNase A (VWR Life Science, Solon, OH, USA). After incubation at 37°C for 1 h, DNA was precipitated by adding 2x the volume of absolute ethanol and left at −20°C for 1 h. The DNA was then centrifuged at 10,000 rpm for 5 m, the supernatant was discarded, and then the DNA pellet was washed with 70% alcohol. After 5 m of centrifugation at 10,000 rpm, the pellet was air-dried at room temperature and then resuspended in ddH_2_O for downstream testing.

### 2.6. Whole-genome sequencing

DNA samples were measured in concentration and quality using the Qubit dsDNA BR (Broad-Range) Assay Kit (Life Technologies, Carlsbad, CA, USA) and were visualized on 1% agarose gel. The library preparation of the DNA samples was carried out using the NEBNext^®^ Ultra™ DNA Library Prep Kit for Illumina^®^ (New England Biolabs, Ipswich, MA, USA), following the manufacturer's protocol. The size selection of the adaptor-ligated DNA was performed using AxyPrep Mag PCR Clean-Up (Axygen, Union City, CA, USA), and each sample was then amplified by PCR for 8 cycles with primers that can be annealed with a flow cell to perform bridge PCR. The PCR products were cleaned with AxyPrep Mag PCR Clean-up, the size was validated using Agilent 2100 bioanalyzer (Agilent Technologies, Palo Alto, CA, USA), and quantified with Qubit 2.0 fluorometer (Life Technologies, Carlsbad, CA, USA). The DNA libraries prepared with wild-type H37Rv and induced H37Rv were sequenced using the Illumina HiSeq Next Generation Sequencer (Illumina, San Diego, CA, USA) with a paired-end configuration of 2x150, according to the manufacturer's instructions.

### 2.7. Bioinformatics and data analysis

All sequence reads of each sample were inspected using the read quality control tool FastQC v0.11.9 (www.bioinformatics.babraham.ac.uk/projects/fastqc/) as a fundamental assessment of data quality. Sequence reads were trimmed using trimmomatic v0.39 (Bolger et al., 2014) to exclude low-quality and possibly contaminating adaptor reads that may interfere with downstream analysis. Trimmed, paired-end raw reads of each induced resistant sample were mapped against MTB H37Rv reference genome (GenBank accession number: NC_000962.3) using BWA-mem v0.7.17 (Li, 2013). The coverage of the reference genome was at least 95%, with a mean depth of coverage of at least 30x. Samtools v1.15.1 (Li et al., 2009) was used to convert SAM to BAM format and sort mapped sequences. *In silico* prediction of drug resistance profiles and lineages was performed using the TB-profiler v4.3.0 (Phelan et al., 2019) with the database version tbdb_4738132 and the original parameters. Integrative Genomics Viewer (IGV) (Robinson et al., 2017) was used for the visual exploration of all genomic data.

## 3. Results

### 3.1. Generated induced resistance in H37Rv

After a mycobacterial model of MTB H37Rv was continuously induced, resistance to isoniazid was generated. Isoniazid-resistant H37Rv was generated in 5 passages to reach the proposed concentration. To allow passage, a culture with sufficiently fresh growth development (3–4 weeks) was used to prepare the turbidity of the cell suspension at the 1.0 McFarland standard and inoculated in a fresh Middlebrook 7H10 drug-containing medium.

### 3.2. Genotypic characterization of RAVs generated in isoniazid-resistant H37Rv

**Figure 1:**
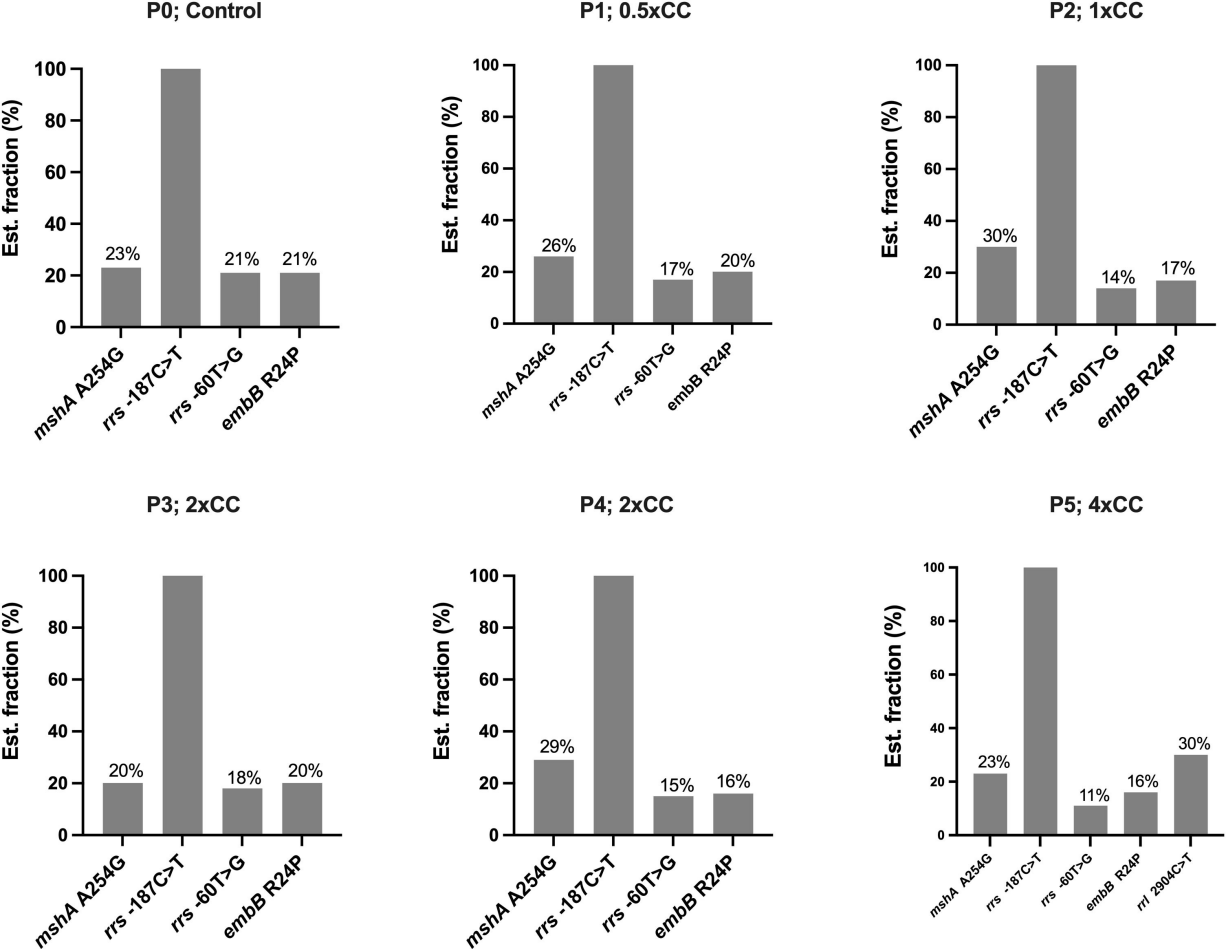
The distribution of variants not associated with isoniazid resistance detected in the *Mycobacterium tuberculosis* H37Rv genome after exposure to isoniazid concentrations ranging from 0.5xCC to 4xCC across passages 1 to 5. The X-axis represents the variants detected on induced concentrations, with variants not associated with isoniazid resistance shown in gray. The Y-axis represents the estimated fraction of variants in percent. P, passage number; CC, critical concentration.

The DST results of H37Rv proved that the mycobacterial model was phenotypically susceptible to isoniazid, rifampicin, ethambutol, and pyrazinamide. Whole genome sequencing was performed in naïve H37Rv for genotypic confirmation of phenotypic DST results and was also used as a genomic background for H37Rv before induction with first-line drugs (Table 1). We identified four variants in the drug-related resistance gene *mshA* A254G —*rrs*−187C>T, *rrs*−60T>G, *rrl* 2904C>T, and *embB* R24P—and these variants do not contribute to drug resistance (Figure 1). Additionally, no significant frequency change was observed in these variants not associated with isoniazid resistance across passages or at higher isoniazid concentrations.

**Table 1:** Drug susceptibility testing (DST) results in each passage performed using the agar proportion method, the BACTEC MGIT 960 system, and whole-genome sequence-based DST.

**Induced passage**	**Phenotypic DST**				**Whole-genome sequence-based DST**			
	**Agar proportion method**			**MGIT 960**	**TB-Profiler v4.3.0**			
	**Isoniazid**	**Rifampicin**	**Ethambutol**	**Pyrazinamide**	**Isoniazid**	**Rifampicin**	**Ethambutol**	**Pyrazinamide**
Naïve (before induction)	Susceptible	Susceptible	Susceptible	Susceptible	Susceptible	Susceptible	Susceptible	Susceptible
1	Susceptible				Susceptible	Susceptible	Susceptible	Susceptible
2	Resistant				Resistant	Susceptible	Susceptible	Susceptible
3	Resistant				Resistant	Susceptible	Susceptible	Susceptible
4	Resistant				Resistant	Susceptible	Susceptible	Susceptible
5	Resistant				Resistant	Susceptible	Susceptible	Susceptible

**Figure 2:**
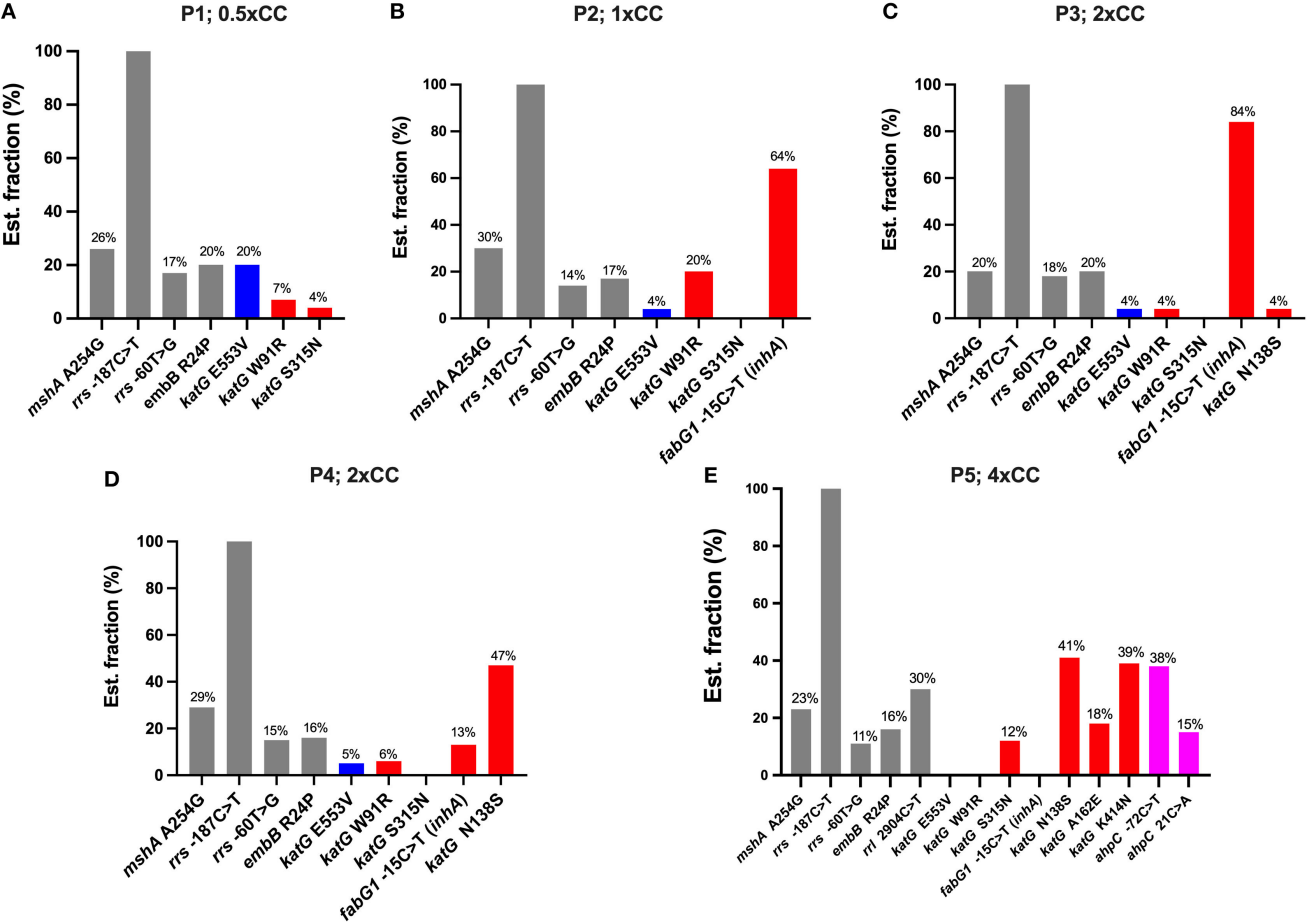
The distribution of variants detected in the *Mycobacterium tuberculosis* H37Rv genome after exposure to isoniazid concentrations ranging from 0.5xCC to 4xCC across passages 1–5. The X-axis represents the variants detected on each induced concentration, variants not associated with isoniazid resistance (gray), low-confidence variants (blue), high-confidence variants/RAVs (red), and suggested compensatory mutations (pink). The Y-axis represents the estimated fraction of variants in percentages. P, passage number; CC, critical concentration.

**Figure 3:**
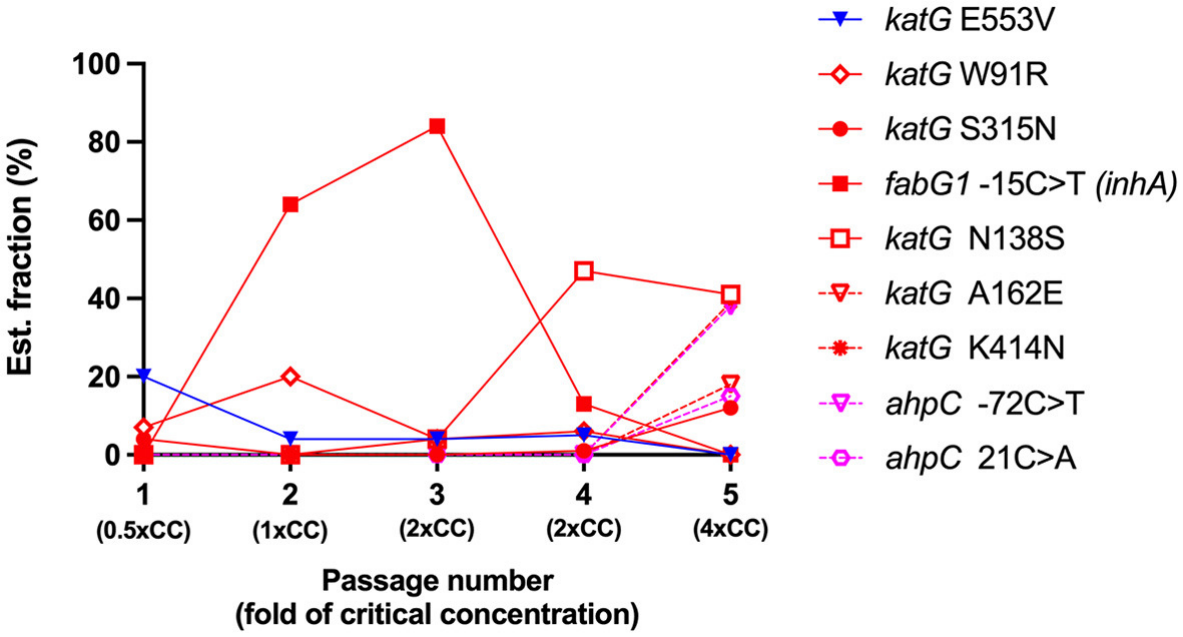
The evolution of variant frequencies in response to increasing concentrations of isoniazid. The X-axis represents the number of induced *Mycobacterium tuberculosis* H37Rv passages and the isoniazid concentration used in each passage. The Y-axis represents the estimated fraction of variants in percent. Each color represents the significance of the variants, the low confidence variant (blue), the high confidence variants/RAVs (red), and the suggested compensatory mutations (pink). CC, critical concentration.

In the first passage, a sub-inhibitory concentration (0.5xCC) of isoniazid was used to induce resistance in H37Rv. Three variants were detected on *katG*: *katG* E553V (20% of the estimated fraction) and two small fractions of RAVs —*katG* W91R and *katG* S315N at 7 and 4%, respectively (Figure 2A). The mycobacterial cell of the first passage was then induced in another passage with a 2-fold higher concentration of isoniazid, 1xCC. The TB profiler reported it as isoniazid-resistant tuberculosis in concordance with the phenotypic DST result (Table 1). The *inhA* promoter mutation (*fabG1*−15C>T) was found primarily at 64% in this passage, along with *katG* W91R (20%) and a small amount of *katG* E553V (4%), while *katG* S315N was not found in this passage (Figure 2B). In the third passage, at 2xCC, we found that the constant increase in *fabG1*−15C>T to 84% alone, while *katG* E553V remained the same as in the previous passage and *katG* W91R decreased significantly to 4%. Furthermore, we found that *katG* N138S was raised for the first time at this concentration (Figure 2C). We observed a marked decrease in *fabG1*−15C>T to 13% and a marked increase in *katG* N138S to 47% in this fourth passage at 2xCC (Figure 2D). The change in the amount of these two RAVs may indicate the adaptation of the resistance mechanism that the mycobacterium exploited in the high concentration of the isoniazid environment. Surprisingly, *fabG1*−15C>T, *katG* W91R, and *katG* E553V were not found in the fifth passage at 4xCC. Besides, we found that *katG* S315N increased to 12%, while *katG* N138 and *katG* K414N were detected at 41% and 39%, respectively. Further, four variants in 2 genes (*katG* and *ahpC*) were found only in 4xCC—*katG* A162E, *katG* K414N, *ahpC*−72C>T, and *ahpC* 21C>A (G7R) (Figure 2E). Taken together, we plotted a graph to track the evolution of each variant discovered from passages 1 to 5, from 0.5xCC to 4xCC (Figure 3). Each variant's rise and fall patterns represented the mechanisms that the mycobacterium used under isoniazid stress. The alteration of the KatG mycobacterial catalase-peroxidase mechanism was discovered in dealing with the sub-inhibitory concentration of isoniazid. The detection of the *inhA* promoter mutation in the second passage or in 1xCC could provide resistance to isoniazid in the first place by increasing the production of the inhA protein and neutralizing the active INH processed by the KatG enzyme (Ramaswamy and Musser, 1998; Tekwu et al., 2014). Up-regulation of the *inhA* promoter could take the fitness cost of the mycobacterial cell by interrupting the lipid metabolism of the mycobacterial cell. In higher concentrations of isoniazid (2x – 4xCC), we observed the adaptation of the resistance mechanism, as we found the decreasing until the absence of the *inhA* promoter mutation. In contrast, *katG* mutations were found in a large number of fractions along with *ahpC* mutations. As a result, at higher concentrations of isoniazid, the mycobacterium may benefit from changing KatG alone to combat INH stress rather than increasing InhA protein production (Figure 3), which may affect overall cell metabolism due to the disruption of lipid metabolism. Our findings may shed light on the impact of isoniazid in generating RAV, as well as the resistance mechanism scenario that mycobacterium used under various isoniazid-pressuring conditions.

## 4. Discussion

Isoniazid is a prodrug that must be intracellularly converted by mycobacterial catalase-peroxidase KatG to an active form, isonicotinoyl. The activated form of isoniazid can interact with NAD+, and the isonicotinoyl-NAD+ complex could fit into the NADH binding pocket of inhA, which is a key enzyme in the synthesis of mycobacterial mycolic acid (Zhang et al., 1992, 1993). Generally, inhA is an enoyl acyl carrier protein reductase that plays a role in mycolic acid synthesis and utilizes NADH as the cofactor (Banerjee et al., 1994; Quemard et al., 1995). However, in the presence of isoniazid, the affinity of InhA to the isonicotinoyl-NAD+ complex is 200 times higher than that of NADH itself. Any mutation that lowers KatG's ability to activate isoniazid will confer resistance to isoniazid, and most HR-TB (47–90%) carried the mutation within *katG* (Heym et al., 1995; Hazbón et al., 2006). Furthermore, mutations in the upstream region of *inhA* result in increased levels of *inhA* expression, hence elevating the InhA protein and inducing isoniazid resistance through a titration mechanism, the state that isoniazid levels cannot fully inhibit the amount of InhA (Ramaswamy and Musser, 1998).

We generated *in vitro* isoniazid-resistant H37Rv according to Ismail's protocol (Ismail et al., 2018). We also mimicked clinical conditions when patients did not adhere to the anti-TB drug or the drug concentration in the blood was less than the therapeutic concentration (Wang et al., 2019). This was done by inducing MTB H37Rv with isoniazid at a sub-inhibitory concentration (0.5xCC, 0.1 μg/ml) that cannot completely kill mycobacterial cells. At this concentration, we found three variants of the *katG* gene: one was an unknown significant variant, *katG* E553V; and two RAVs, *katG* W91R and *katG* S315N. Several amino acid substitutions that reduced the stability or activity of the KatG enzyme have been reported (Portelli et al., 2018). This finding suggests a common mechanism by which mycobacterium responds to the presence of isoniazid. However, this was still not enough to make the mycobacterium resist isoniazid, and we performed the agar proportion as phenotypically DST in parallel with the generation of induced resistance. At 1xCC, genotypically, DST was consistent with pDST in which isoniazid-resistant tuberculosis was observed. The *inhA* promoter−15C>T mutation was predominantly found and could play an important role in isoniazid resistance in this passage by synthesis of more InhA protein than the amount of active isoniazid that can fully inhibit and thus confer resistance. Replacement of thymine (T) on the 15^th^ nucleotide (C, cytosine) in front of the *fabG1*-*inhA* operon is the hot spot of the *inhA* promoter mutation. However, mutations at −8, −17, and −47 contributed to isoniazid resistance and have been reported elsewhere (Seifert et al., 2015). We also found an estimated 20% fraction of *katG* W91R, which is one of the RAV reported as a novel mutation from clinical isolates in Brazil (Cardoso et al., 2004). Together, after comparing the fractions of both genes throughout the mycobacterium genome, we can assume that mycobacterium tends to use the overexpression of the InhA protein as the main resistance mechanism in a combination of alteration of the KatG enzyme. The findings of 2xCC demonstrated that the *inhA* promoter mutation was used primarily after we discovered an increase in *fabG1*−15C>T to 84%, while other katG mutations were significantly reduced (Figure 2C). In our study, the reversible adaptation of the resistance mechanism was observed as the *fabG1* mutation decreased (Figure 2D) and was no longer found at a critical concentration of 4x (Figure 2E); *katG* N138S and *katG* K414N were at 41 and 39%, respectively. These two are uncommon mutations in *katG*, but both reported decreased peroxidase and catalase activity (DeVito and Morris, 2003; Bolotin et al., 2009). Moreover, we found an increasing amount of *katG* S315N, which is the most common region responsible for isoniazid resistance. However, in clinical isolates, the substitution of threonine (T) for 315 amino acids was reported in more than half of isoniazid-resistant clinical isolates, more frequently than asparagine (N) substitutions (Hazbón et al., 2006). The difference in amino acid substitution at this position is a key advantage of the mycobacterium in survival and in becoming a worldwide spread strain, since *katG* S315T retained catalase-peroxidase activity, while S315N had lost both enzyme activities (Ando et al., 2010). We also detected the variant *katG* A162E, which was reported in French clinical isolates in 2016. This mutation displayed similar enzyme activity behavior to S315T, with moderately decreased catalase activity and drastically decreased isoniazid conversion (Brossier et al., 2016).

However, most of the other *KatG* mutations reduce the virulence of the strain, except S315T. This is probably because KatG defends against reactive oxygen species (ROS) of the host immune system (Heym et al., 1997; Ng et al., 2004) and could protect harmful peroxides produced during mycobacterial oxidative metabolism (Manca et al., 1999). The loss of KatG activity can be compensated by increasing the expression of alkylhydroperoxidase, AhpC. A mutation in *ahpC* alone cannot produce isoniazid-resistant tuberculosis and is also undetectable in drug-susceptible tuberculosis. However, it can be found in some isoniazid-resistant strains that have mutations within *katG*. The increased expression of *ahpC* is usually a result of mutations at its promoter site (Sherman et al., 1996). We found two mutations in *ahpC*, one in the promoter region, which is *ahpC*−72C>T, and *ahpC* 21C>A (G7R) (Figure 2E). These suggested compensatory mutations might play a role in compensating for the loss of KatG activity due to the accumulation of *katG* mutations. However, *ahpC* promoter mutations can occasionally be detected in some isoniazid-susceptible strains without alterations in the *katG* gene (Hazbón et al., 2008; Casali et al., 2014). Moreover, we detected the specific emergence of the *rrl* 2904C>T variant in passage 5 (Figure 2E). This variant is not typically associated with isoniazid resistance, as reported in previous literature (Nguyen et al., 2018; Alame Emane et al., 2021). The *rrl* gene, encoding the 23S rRNA, is primarily linked with resistance to antibiotics that target the bacterial ribosome, particularly linezolid (Kadura et al., 2020). Therefore, the reasons for the *rrl* 2904C>T occurring only in passage 5 remain elusive.

In contrast, there is one study that reported that resistant mutants of MTB selected *in vitro* did not represent the *in vivo* resistance mechanism of isoniazid since the study did not detect clinically prevalent mutations in *katG, inhA*, and *ahpC*. These could be from the *in vitro* model; the selection of spontaneous mutants might not represent the *in vivo* situation (Bergval et al., 2009). However, our approach has successfully led to isoniazid-resistant mutants, and those mutations are clinically relevant, such as *fabG1*−15C>T and *katG* S315N. The resistance induction method has been demonstrated to generate clinically significant RAVs and offers initial insights into the underlying mechanisms of resistance emergence. Our findings provide a basic understanding of the development of a resistance process through the generation of the RAVs, which was observed during every passage, even though the experiment was performed once. Additionally, the WGS data obtained from each passage can help draw a clearer picture of the resistance mechanisms that develop under different isoniazid conditions, including both high and low concentrations of isoniazid.

There are several limitations to this study that should be considered. Firstly, we did not perform whole genome sequencing on DNA extracted from a single colony. Instead, we used multiple loops from various colonies that had grown in each passage, which may have resulted in the lack of certainty that the same MTB variants carried all observed mutations. Therefore, caution should be taken when interpreting our findings, as detected mutations might originate primarily from the predominant isoniazid-resistant MTB variants rather than assuming that they are all carried by a single MTB variant. Secondly, it is important to note that our analysis tool, TB-profiler, mainly identified variants on genes primarily associated with resistance. This tool provided comprehensive insights into these specific genes; however, its analysis did not extend to the entire genome. Consequently, certain non-canonical genes potentially contributing to antibiotic stress adaptation, such as those involved in efflux pump systems or membrane permeability, have not been fully examined in our analysis. Thirdly, we identified mutations in the *ahpC* gene in passage 5 but did not experimentally confirm whether these mutations were compensatory. Therefore, further research, including complementary experiments such as conducting functional assays to test enzymatic activity or oxidative stress response. Conversely, differential gene expression analysis in MTB variants is necessary to fully understand the implications of these mutations and their potential effects on the fitness of MTB variants during oxidative stress or host-pathogen interactions. Moreover, comprehensive whole-genome sequencing on individual colonies for verifying each mutation is also needed.

Eventually, the generation of induced resistance by continually exposing mycobacterium at low concentrations until it becomes resistant may be more representative of the *in vivo* situation and may also be driven toward high-level resistance. The study of RAVs generated by several passages could provide more clinically relevant details. Our *in vitro* findings suggest the occurrence of mechanical resistance events in the induced H37Rv strain after exposure to sub-inhibitory concentrations of isoniazid, up to four times the critical concentration. Mycobacterium exploits (1) overexpression of the InhA protein through a mutation in the promoter region at 1xCC and then switches to (2) alteration of KatG at high levels of isoniazid, along with detected *ahpC* mutations.

## 5. Conclusions

In conclusion, we demonstrated the impact of isoniazid on mechanical resistance events and on the generation of RAVs. This knowledge could lead to a better understanding of the mechanisms of acquisition of resistance to MTB over time. To further advance this field, additional research is needed to develop a resistance prediction platform using RAV information from each induction period. The prediction platform, developed utilizing accurate large RAVs databases, could potentially promote successful treatment by enabling clinicians to predict the probability of drug resistance and select appropriate drugs for MDR and XDR-TB patients.

## Data availability statement

The data presented in the study are deposited in the Sequence Read Archive (SRA), NCBI repository, accession number: PRJNA939919.

## Author contributions

PN contributed to the conception and design of this study and assembled the final version of the manuscript. TD performed the generation of induced resistance, phenotypic DST, DNA extraction, and majority analysis of the WGS data. OT provided *M. tuberculosis* H37Rv and a drug-resistant strain from the mycobacterial laboratory at the Siriraj Hospital Department of Microbiology, advised the direction of laboratory processes, and handled the whole genome sequencing samples. KS provided direction in the visualization of WGS data and WGS analysis. TD, OT, and KS worked under the supervision of PN. All authors contributed to this work, review of the manuscript, and approved the submitted version.
