# Supplementary material for: In vitro modeling of isoniazid resistance mechanisms in Mycobacterium tuberculosis H37Rv
Source: Front Microbiol. 2023 Jul 10;14:1171861. doi: 10.3389/fmicb.2023.1171861 (PMC10364472; doi:10.3389/fmicb.2023.1171861)

**Supplementary figures of IGV results in each passage.**

**Passage 1**

| **Passage 1 (0.5x Critical concentration)** | |  |  |  |  |
| --- | --- | --- | --- | --- | --- |
| **Variants detected** | **Estimated fraction (%)** |  |  |  |  |
| *mshA* A254G | 26 |  |  |  |  |
| *rrs* -187C>T | 100 |  |  |  |  |
| *rrs* -60T>G | 17 |  |  |  |  |
| embB R24P | 20 |  |  |  |  |
| *katG* E553V | 20 |  |  |  |  |
| *katG* W91R | 7 |  |  |  |  |
| *katG* S315N | 4 |  |  |  |  |
|  |  |  |  |  |  |
| **The number estimated fractions might not same as all IGV screenshots because mostly retrieved from the TB-profiler reports, and some from IGV for the positions that were not reported by TB-profiler.* | | | | | |


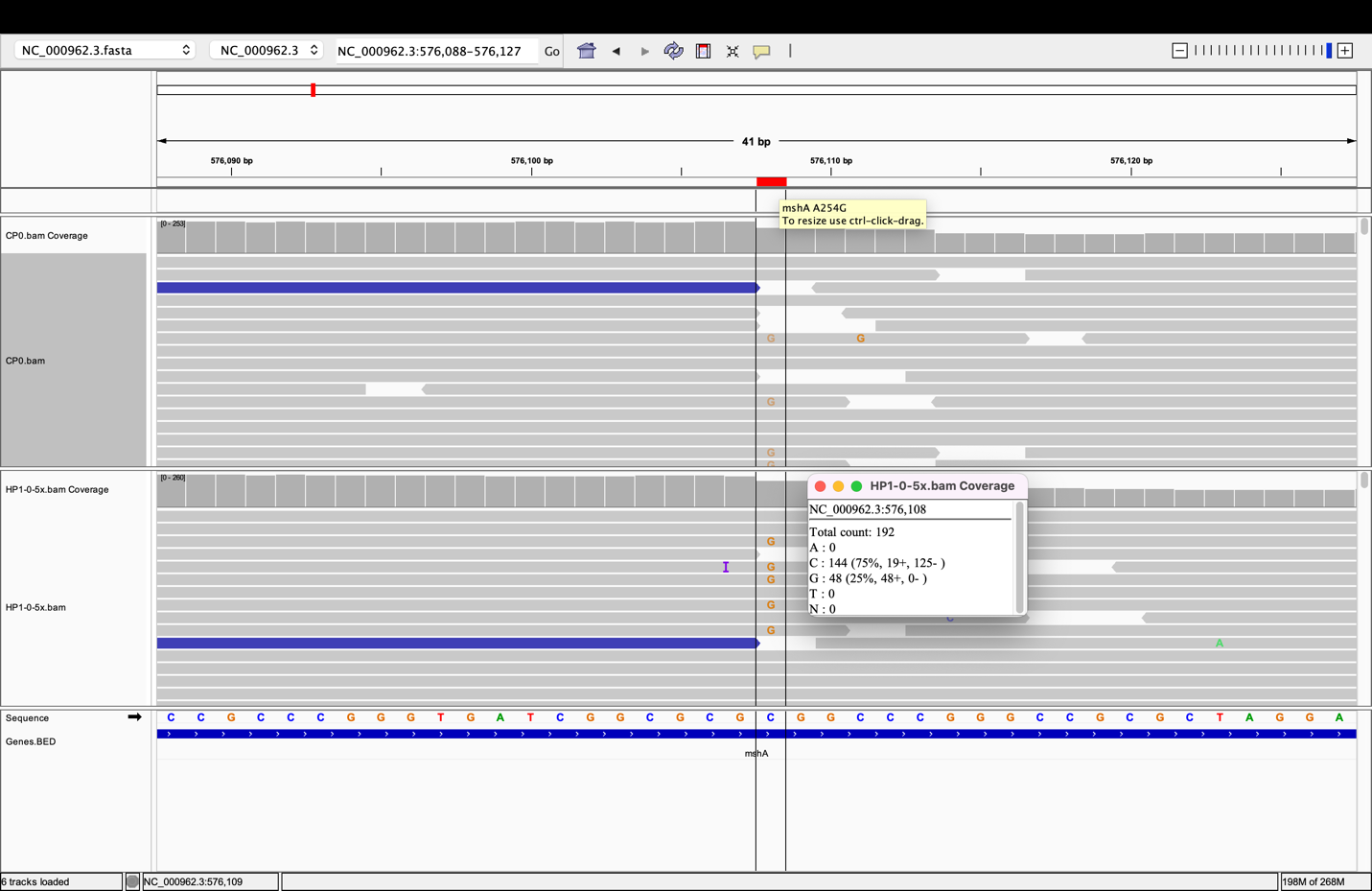


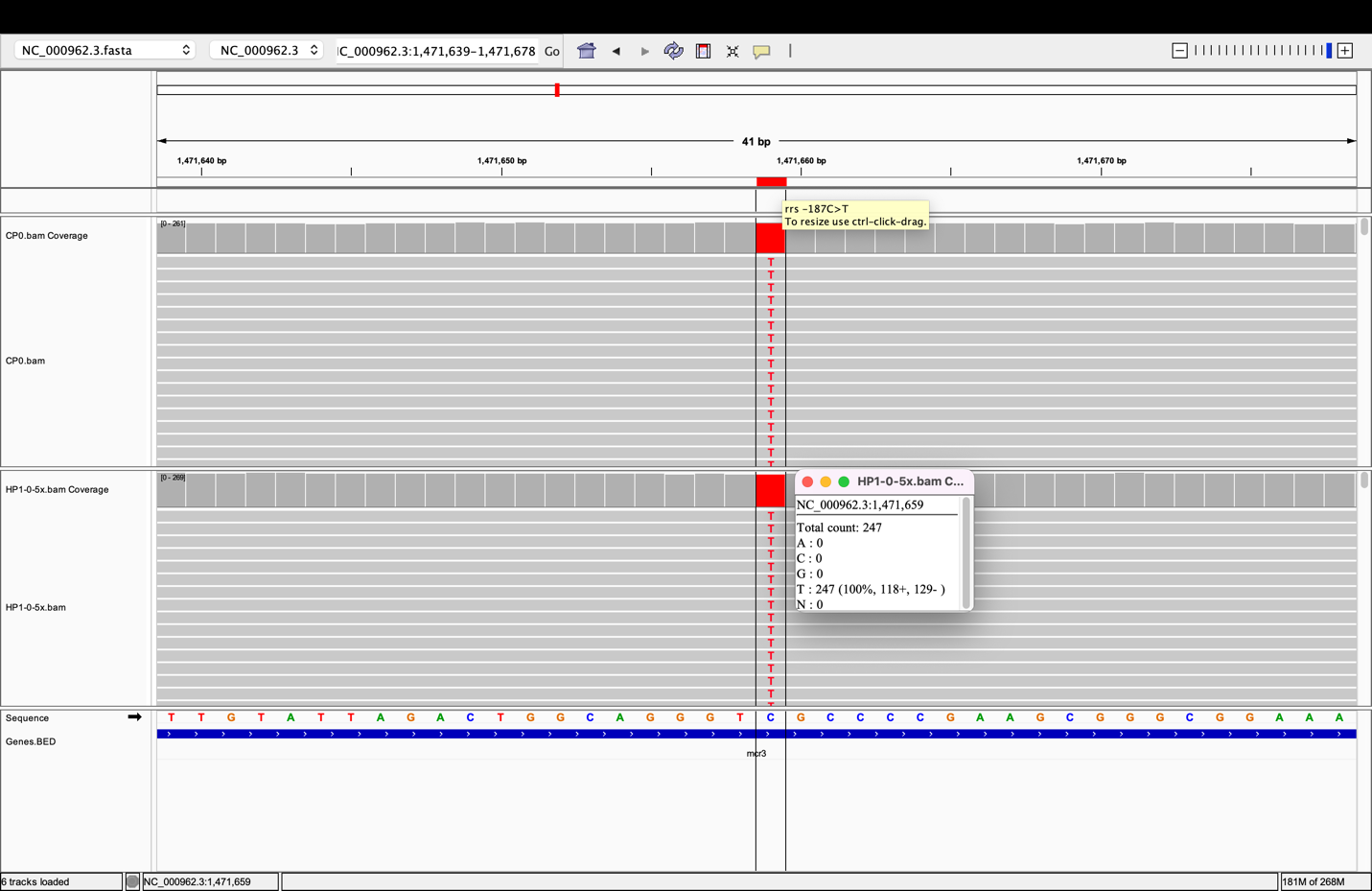


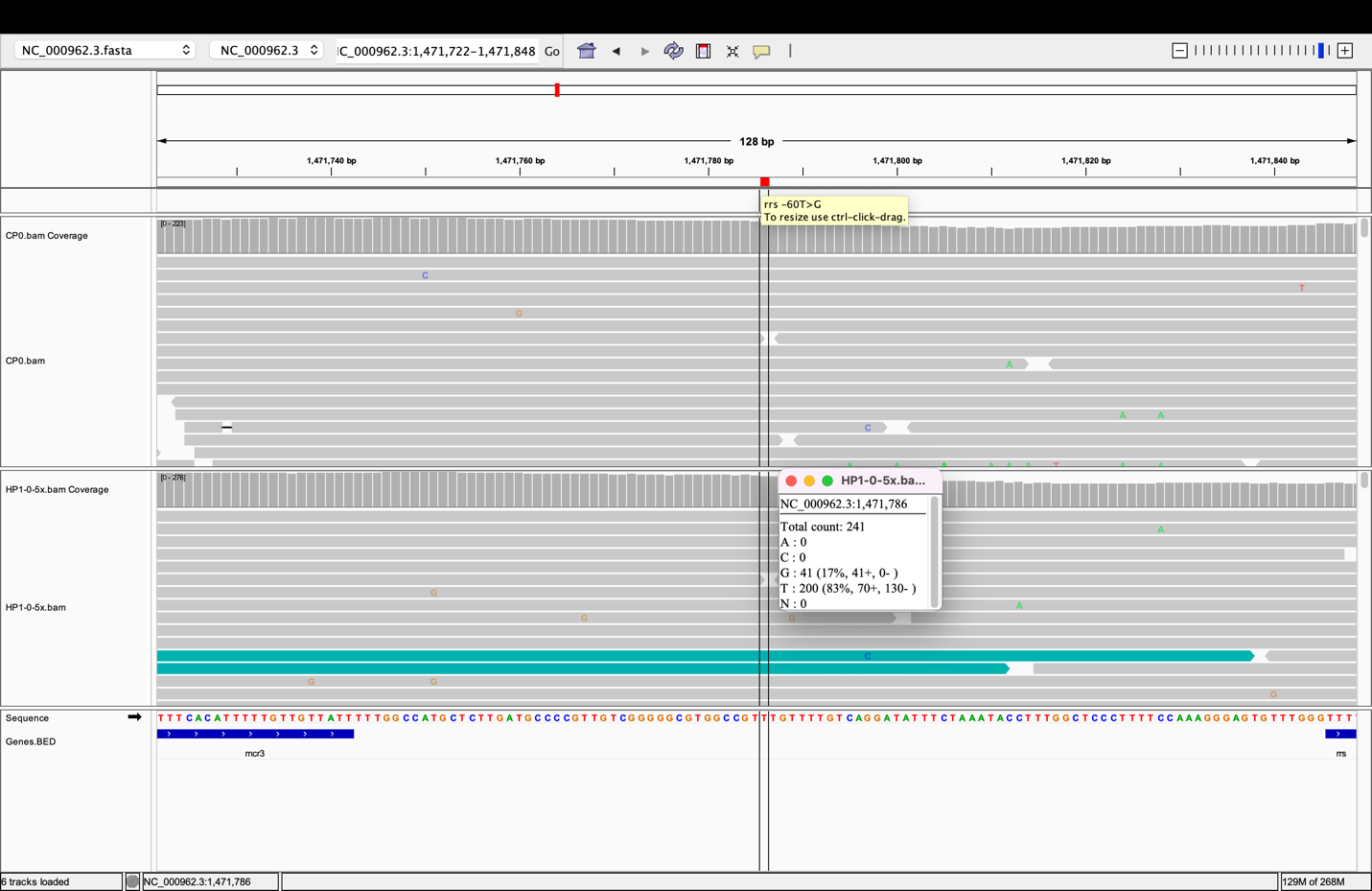


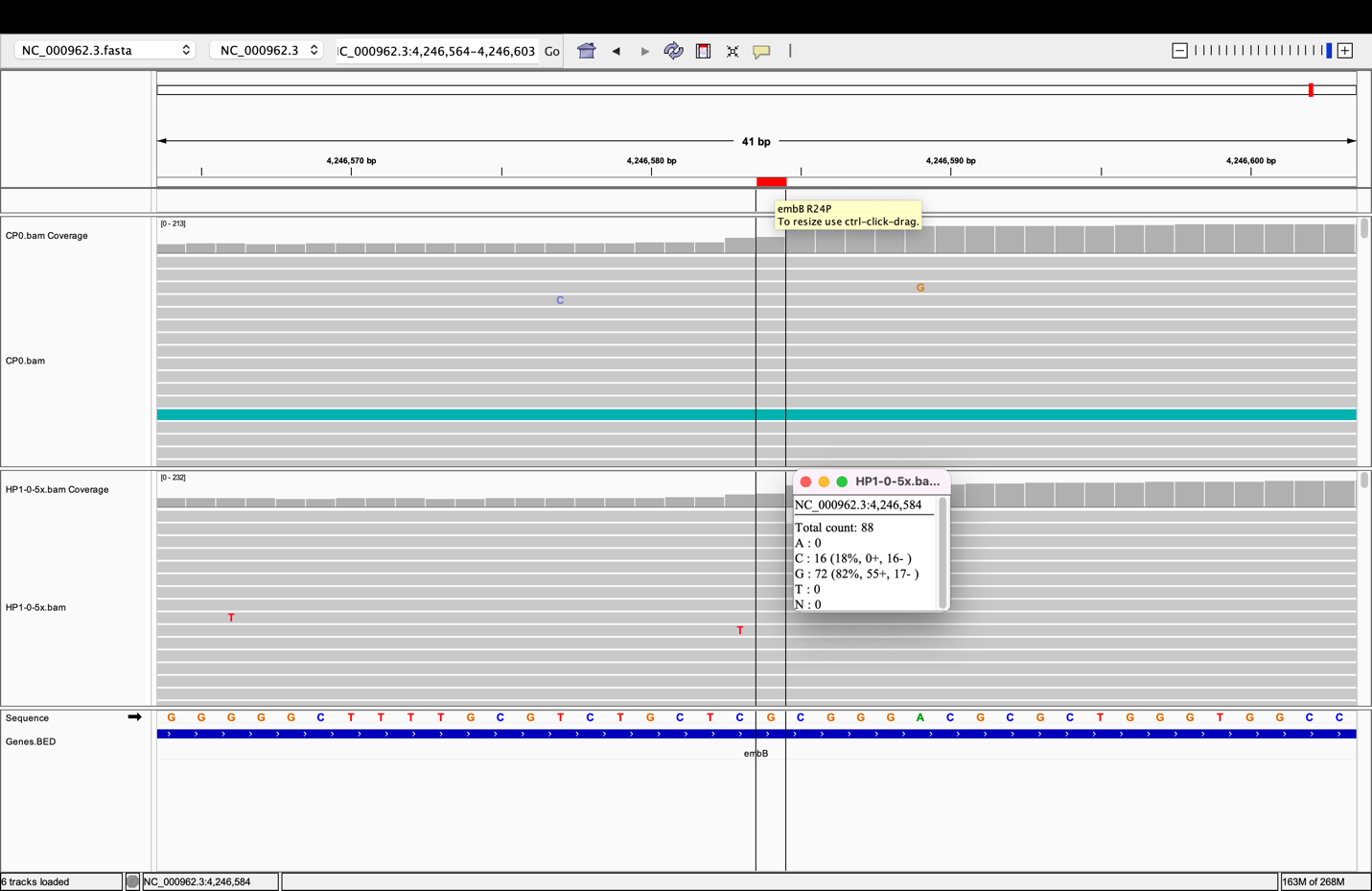


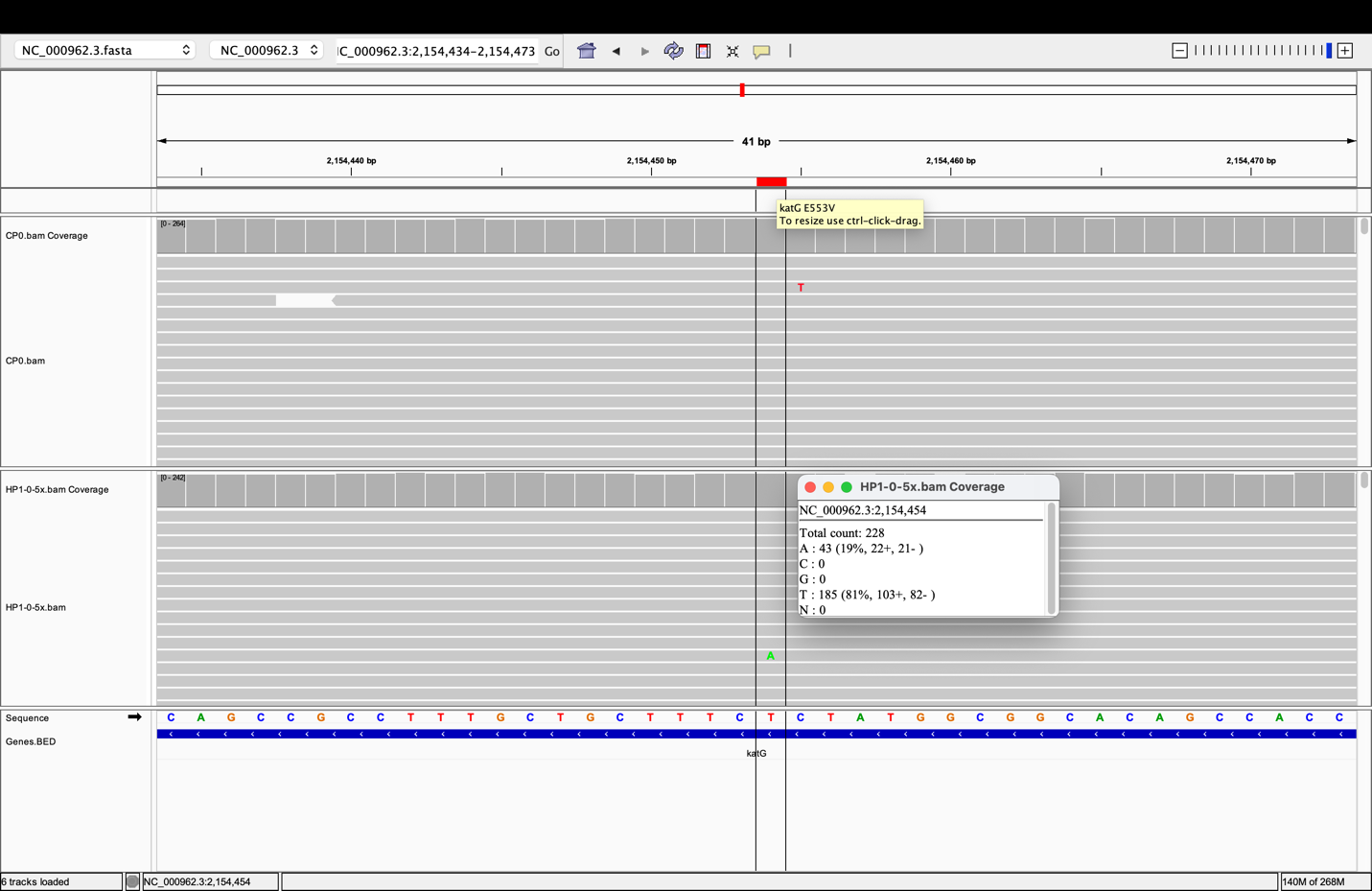


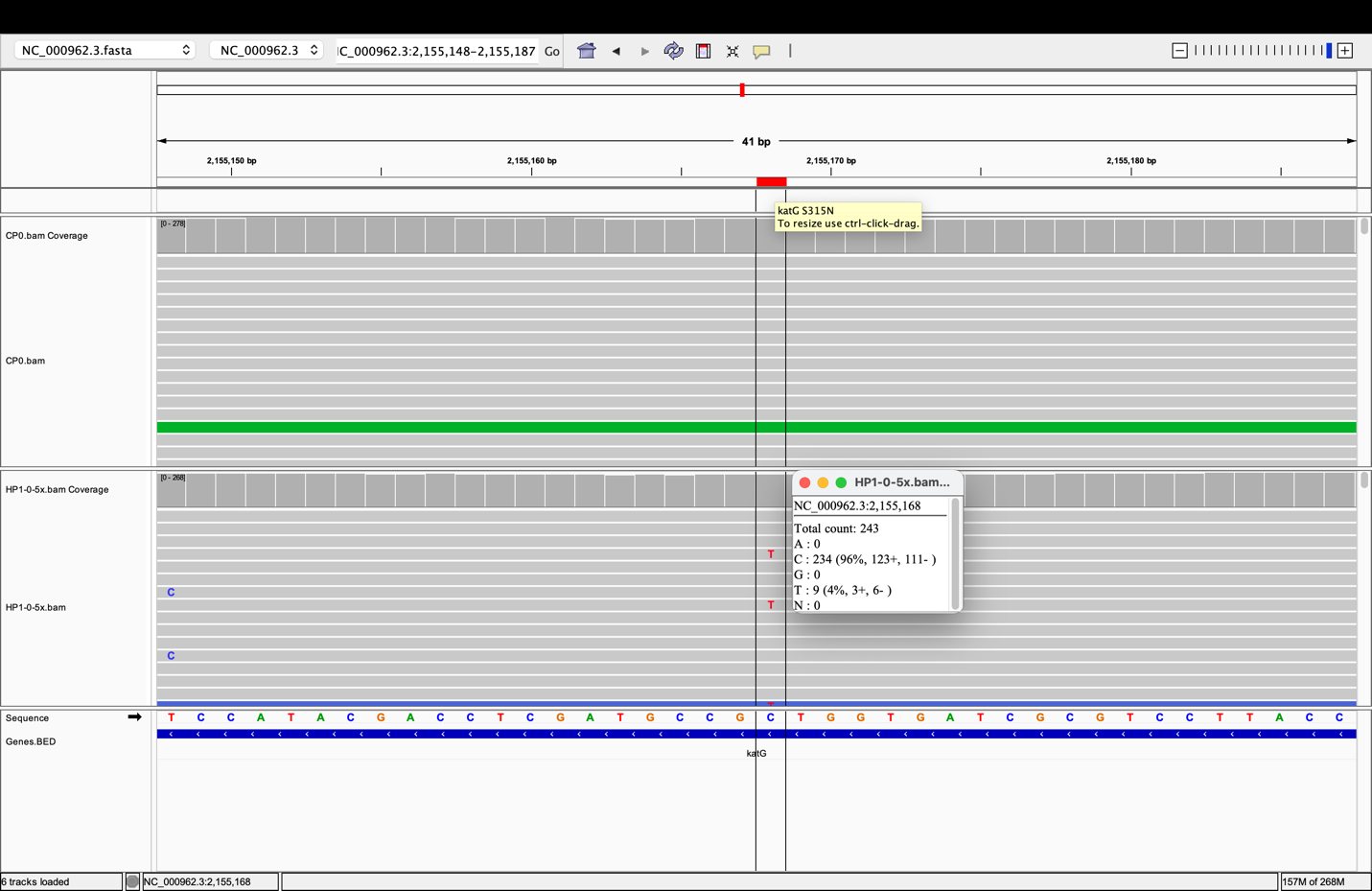


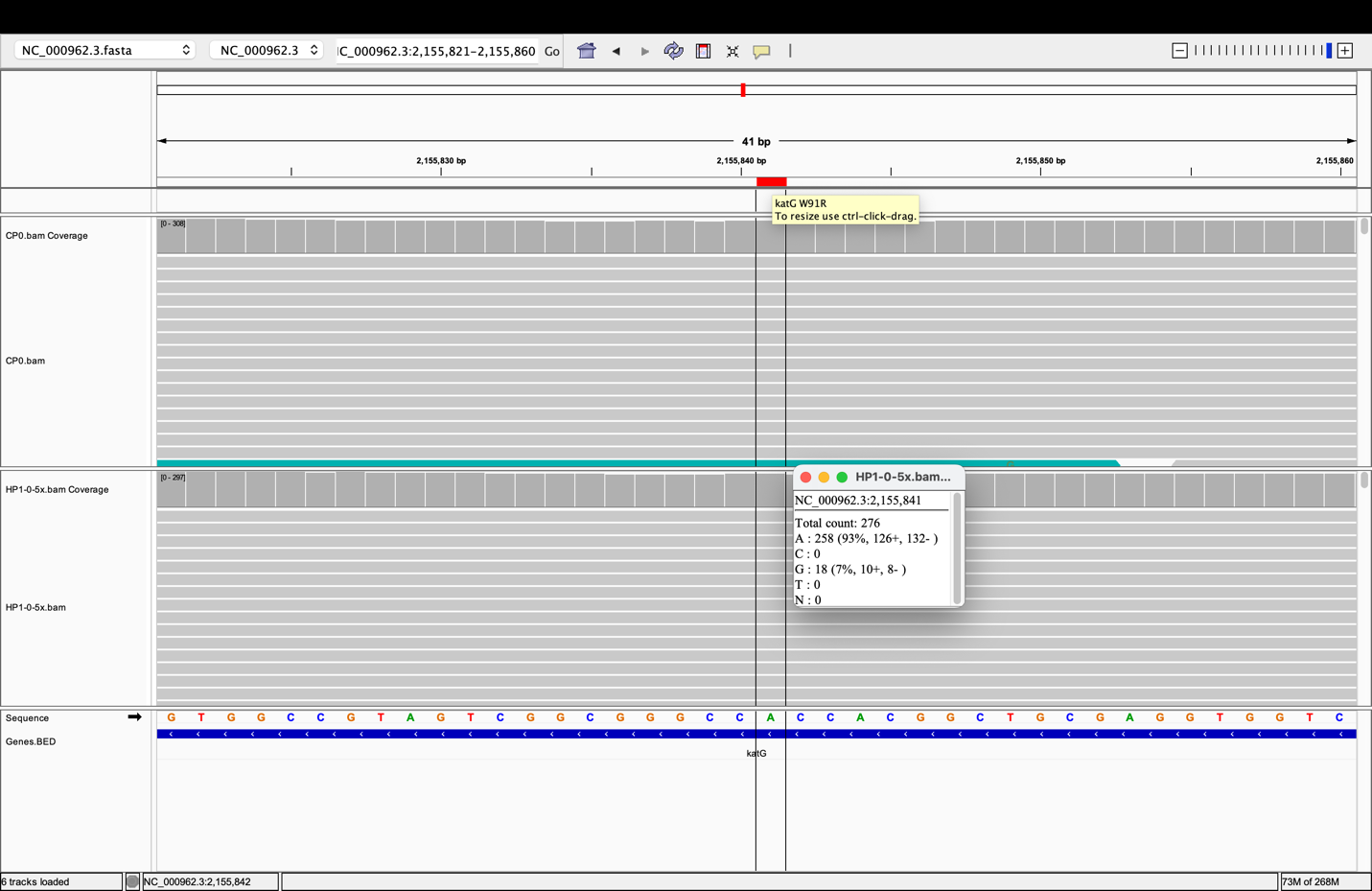


**Passage 2**

| **Passage 2 (1x Critical concentration)** | |  |  |  |  |
| --- | --- | --- | --- | --- | --- |
| **Variants detected** | **Estimated fraction (%)** |  |  |  |  |
| *mshA* A254G | 30 |  |  |  |  |
| *rrs* -187C>T | 100 |  |  |  |  |
| *rrs* -60T>G | 14 |  |  |  |  |
| embB R24P | 17 |  |  |  |  |
| *katG* E553V | 4 |  |  |  |  |
| *katG* W91R | 20 |  |  |  |  |
| *katG* S315N | 0 |  |  |  |  |
| *fabG1* -15C>T | 64 |  |  |  |  |
|  |  |  |  |  |  |
| **The number estimated fractions might not same as all IGV screenshots because mostly retrieved from the TB-profiler reports, and some from IGV for the positions that were not reported by TB-profiler.* | | | | | |


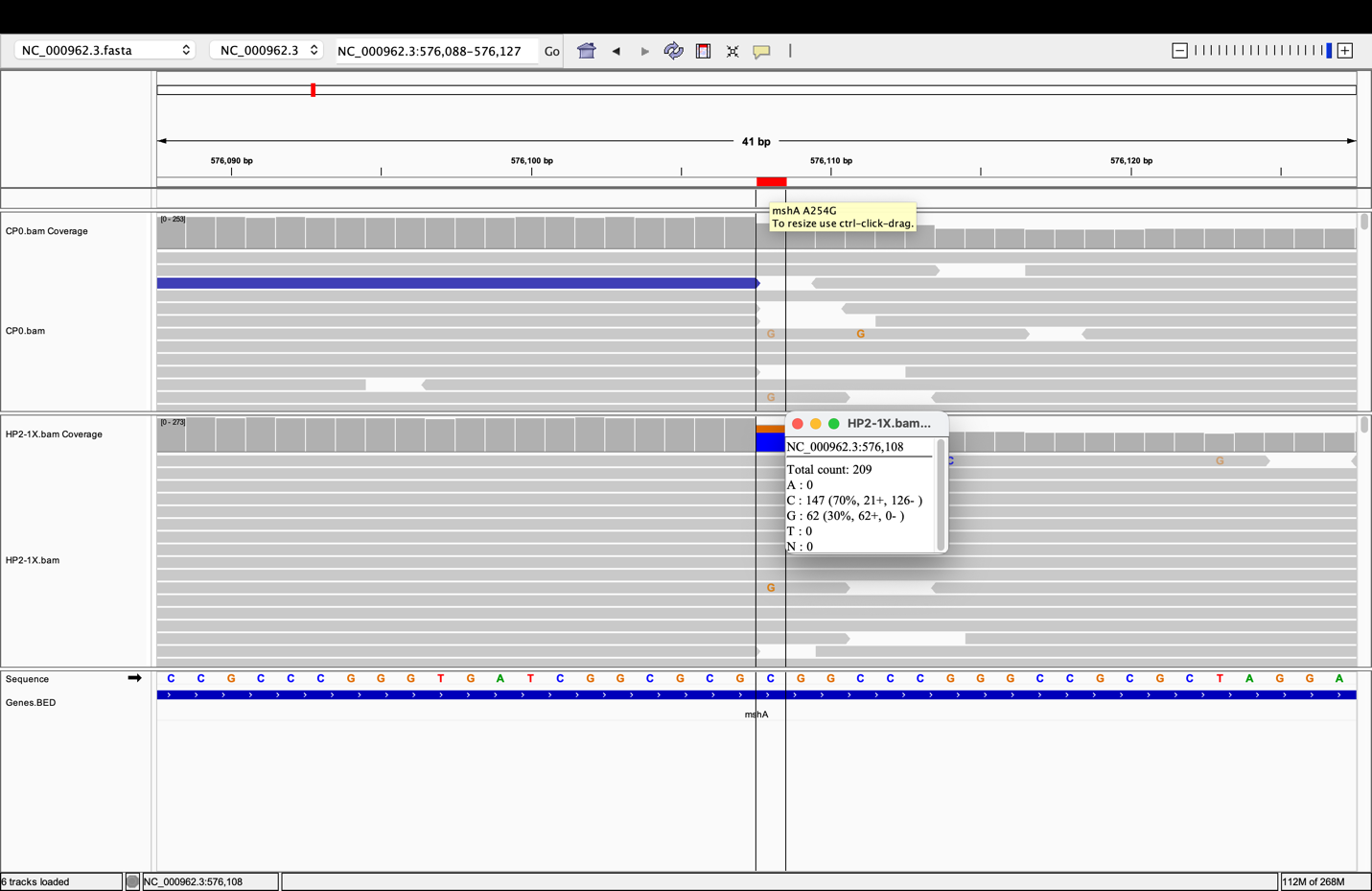


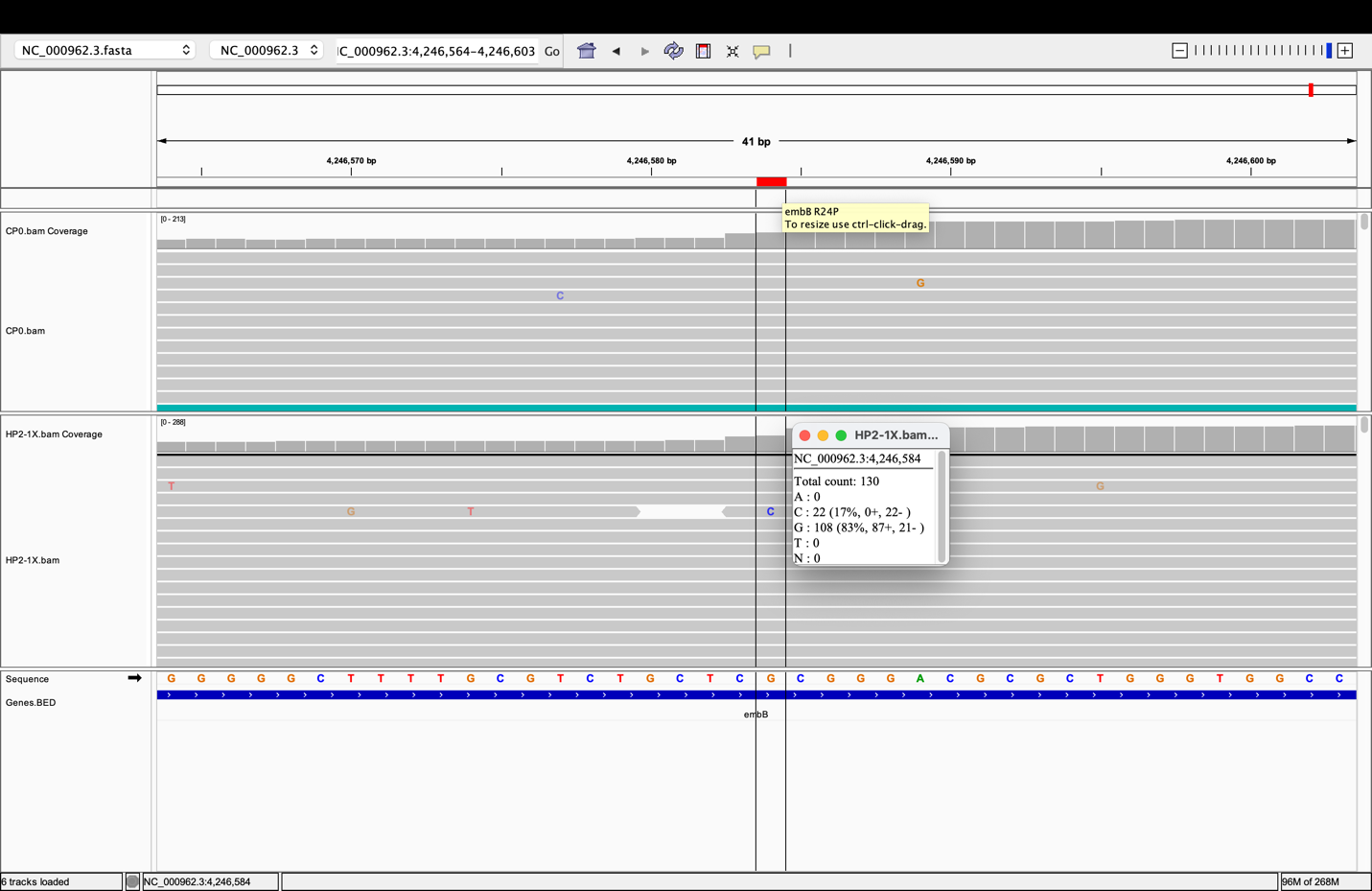

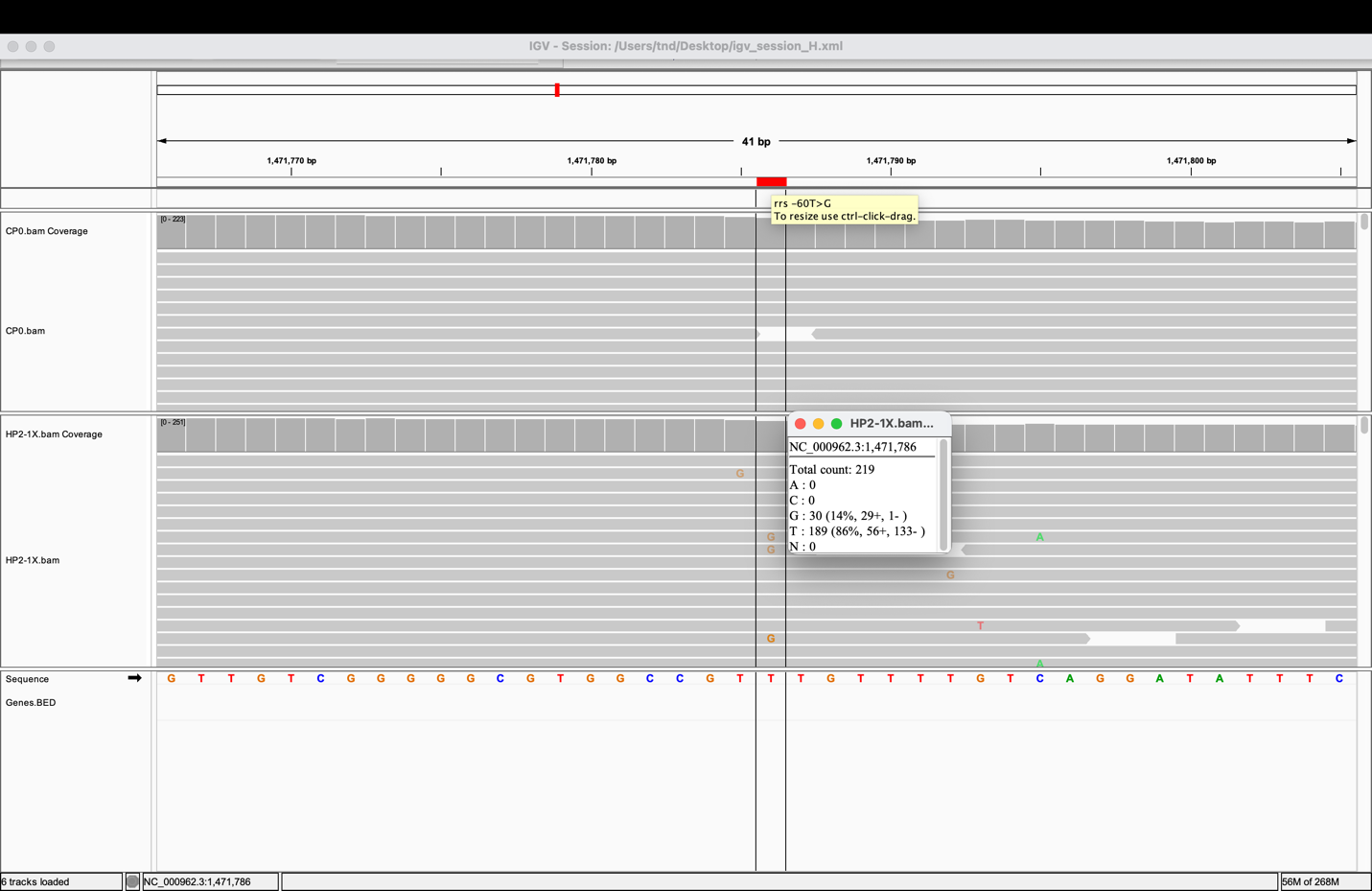

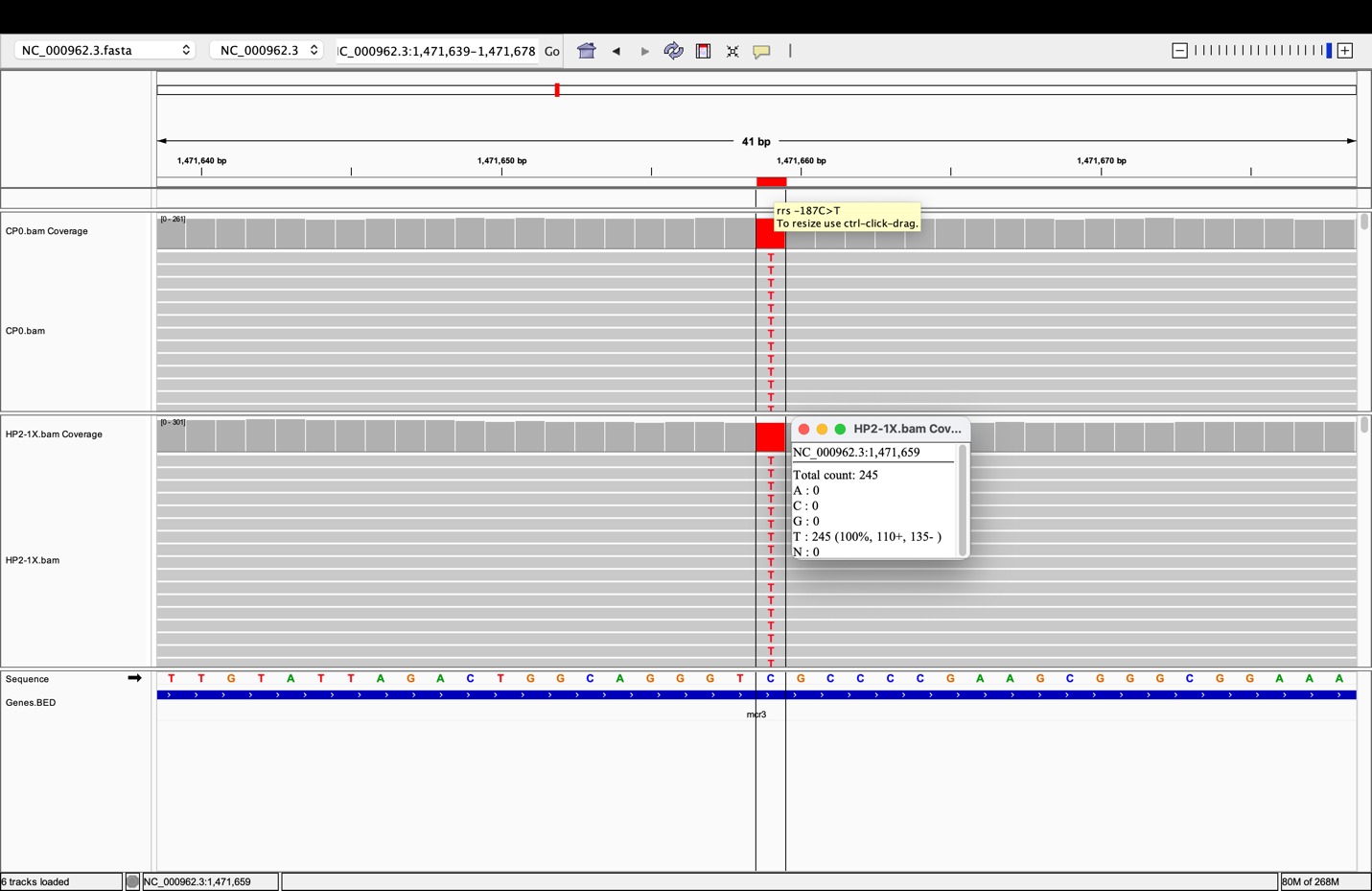


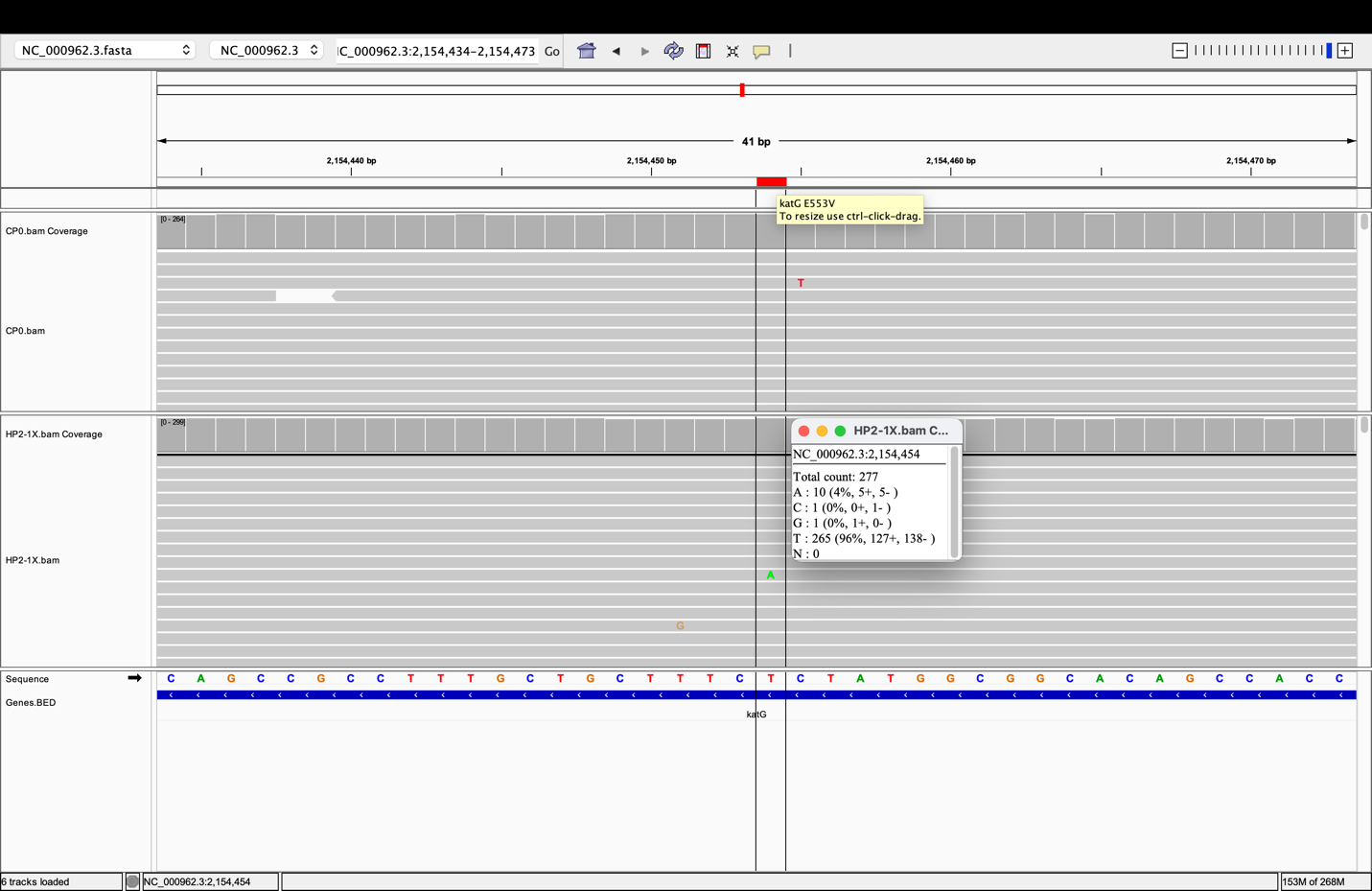

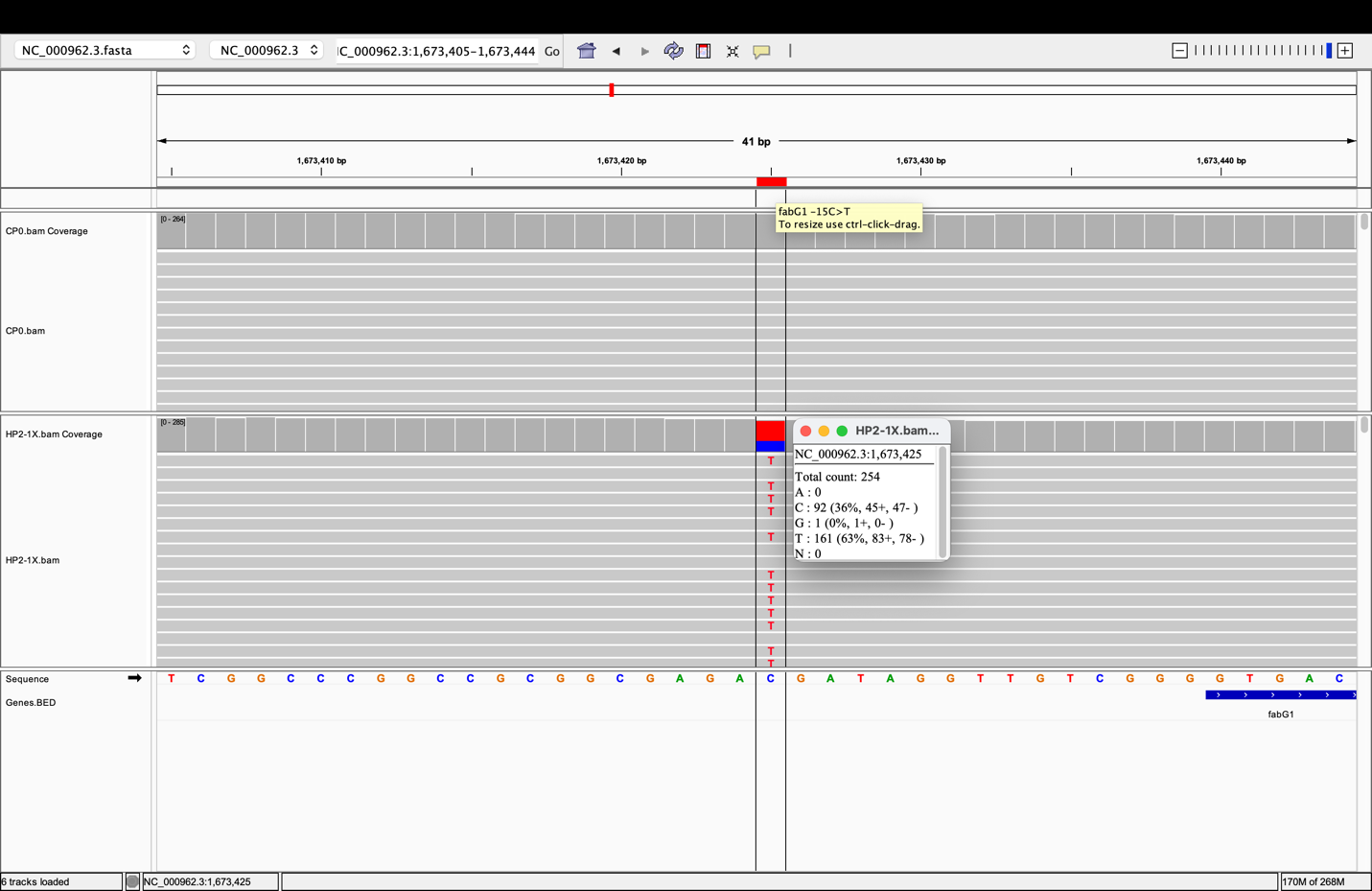

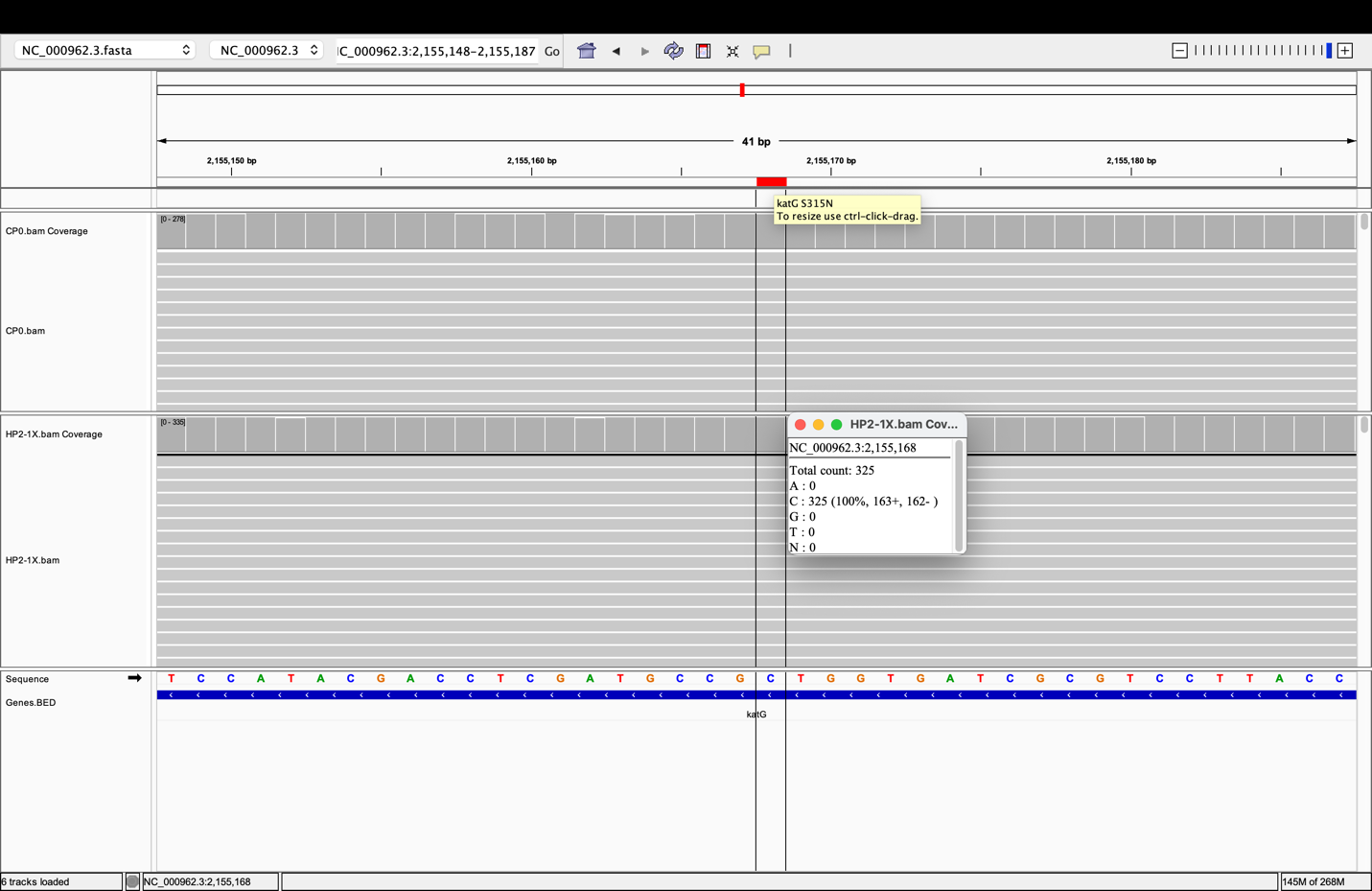

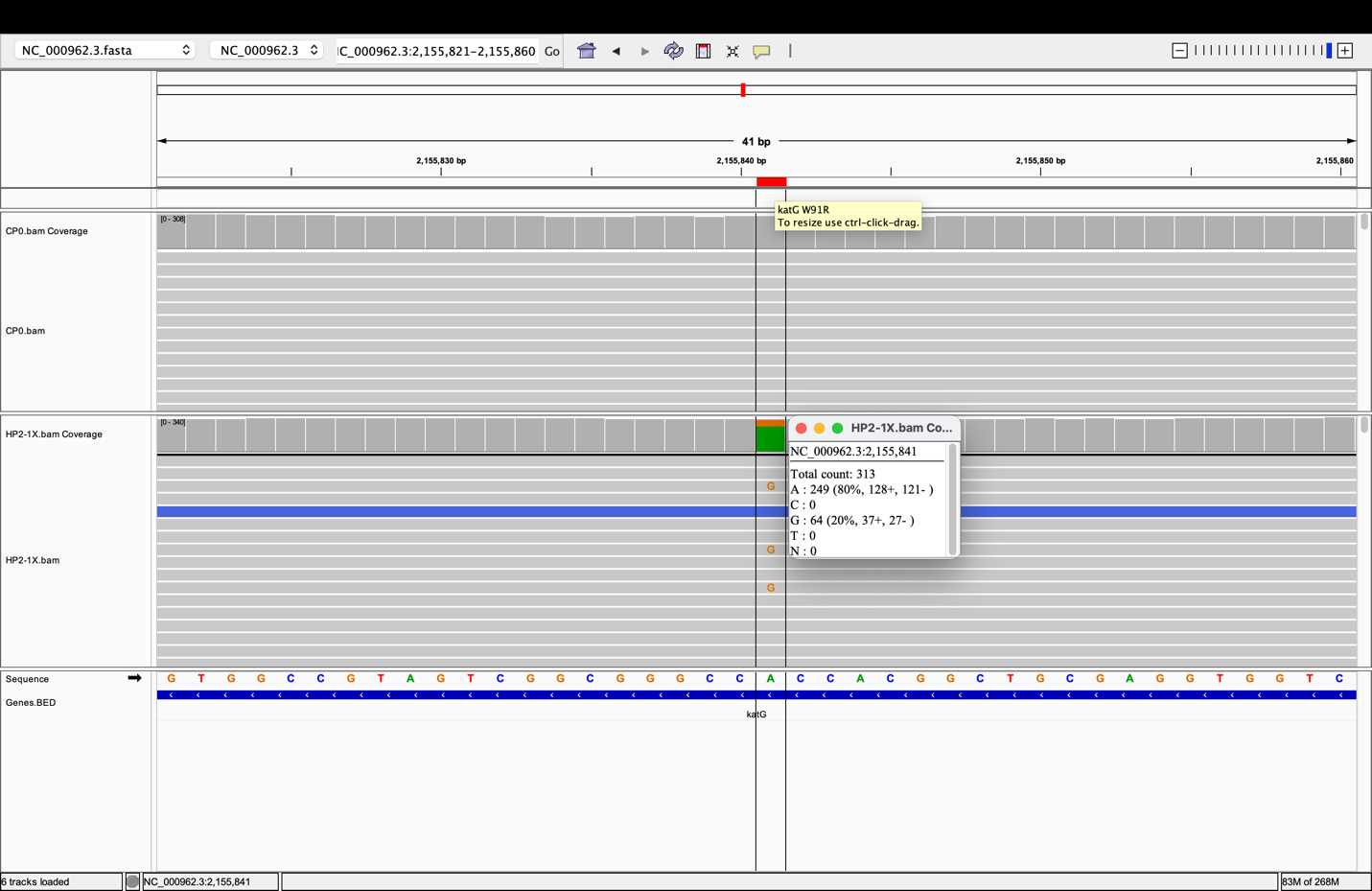


**Passage 3**

| **Passage 3 (2x Critical concentration)** | |  |  |  |  |
| --- | --- | --- | --- | --- | --- |
| **Variants detected** | **Estimated fraction (%)** |  |  |  |  |
| *mshA* A254G | 20 |  |  |  |  |
| *rrs* -187C>T | 100 |  |  |  |  |
| *rrs* -60T>G | 18 |  |  |  |  |
| embB R24P | 20 |  |  |  |  |
| *katG* E553V | 4 |  |  |  |  |
| *katG* W91R | 4 |  |  |  |  |
| *katG* S315N | 0 |  |  |  |  |
| *katG* N138S | 4 |  |  |  |  |
| *fabG1* -15C>T | 84 |  |  |  |  |
|  |  |  |  |  |  |
| **The number estimated fractions might not same as all IGV screenshots because mostly retrieved from the TB-profiler reports, and some from IGV for the positions that were not reported by TB-profiler.* | | | | | |


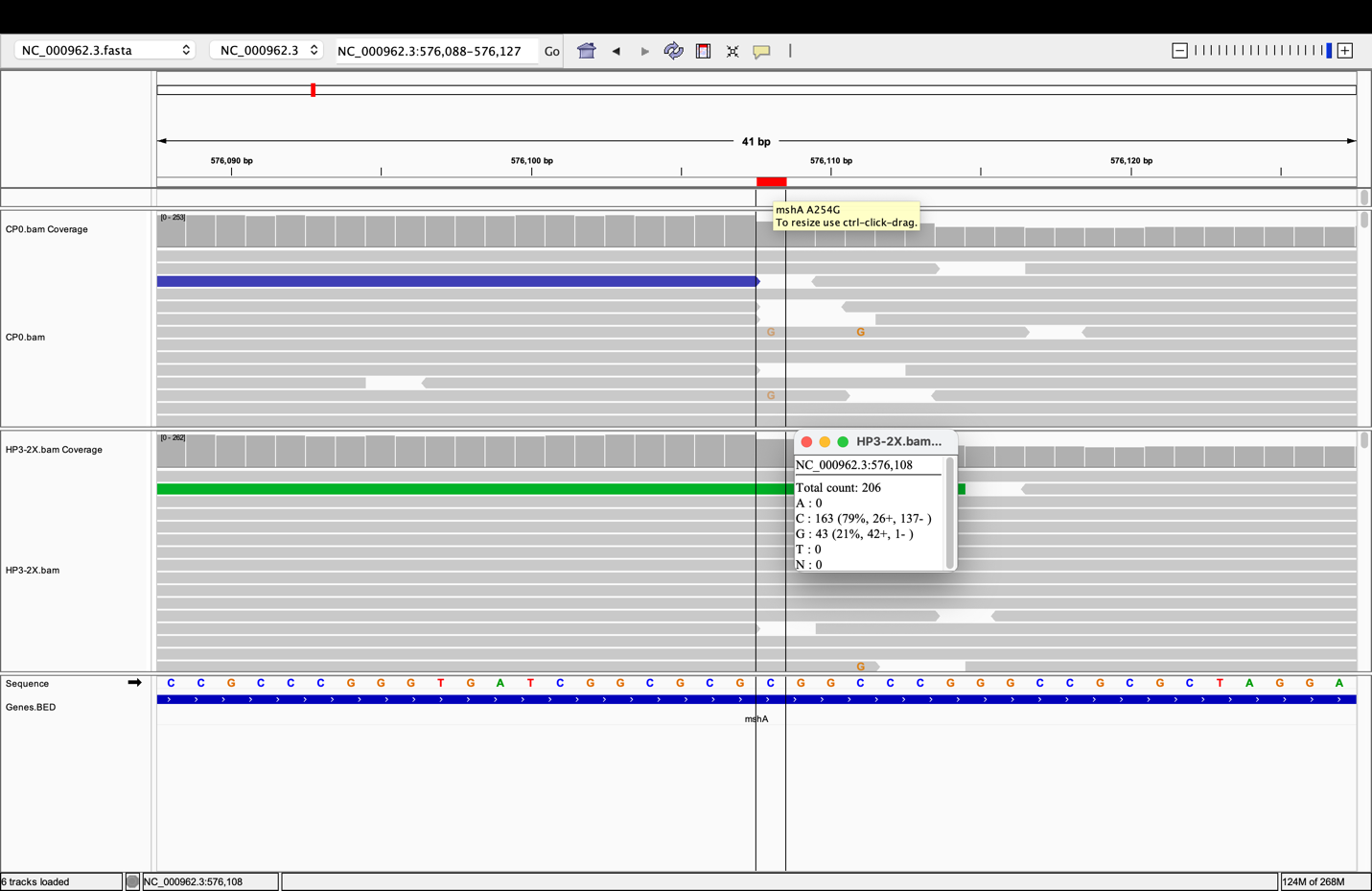


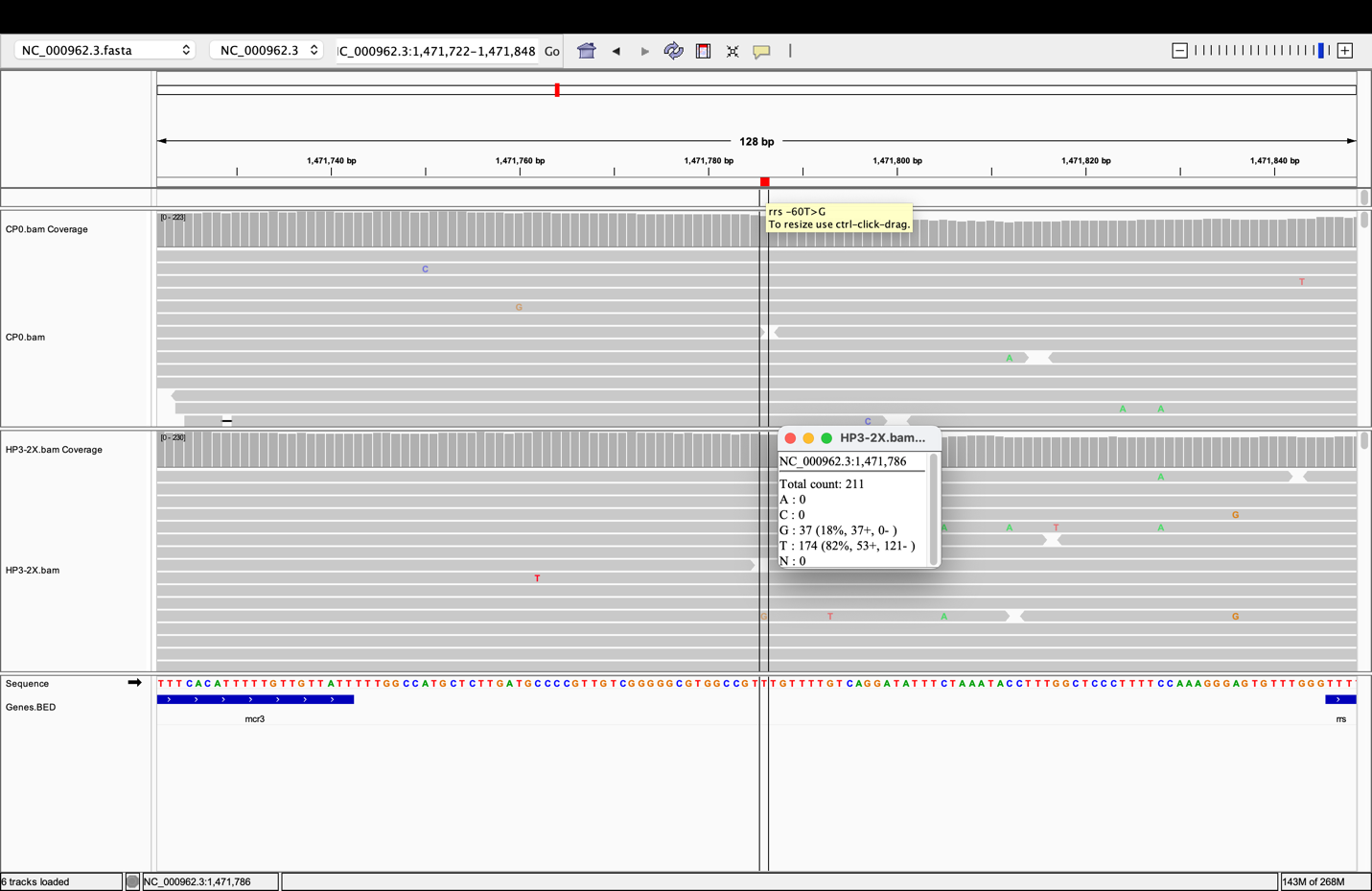

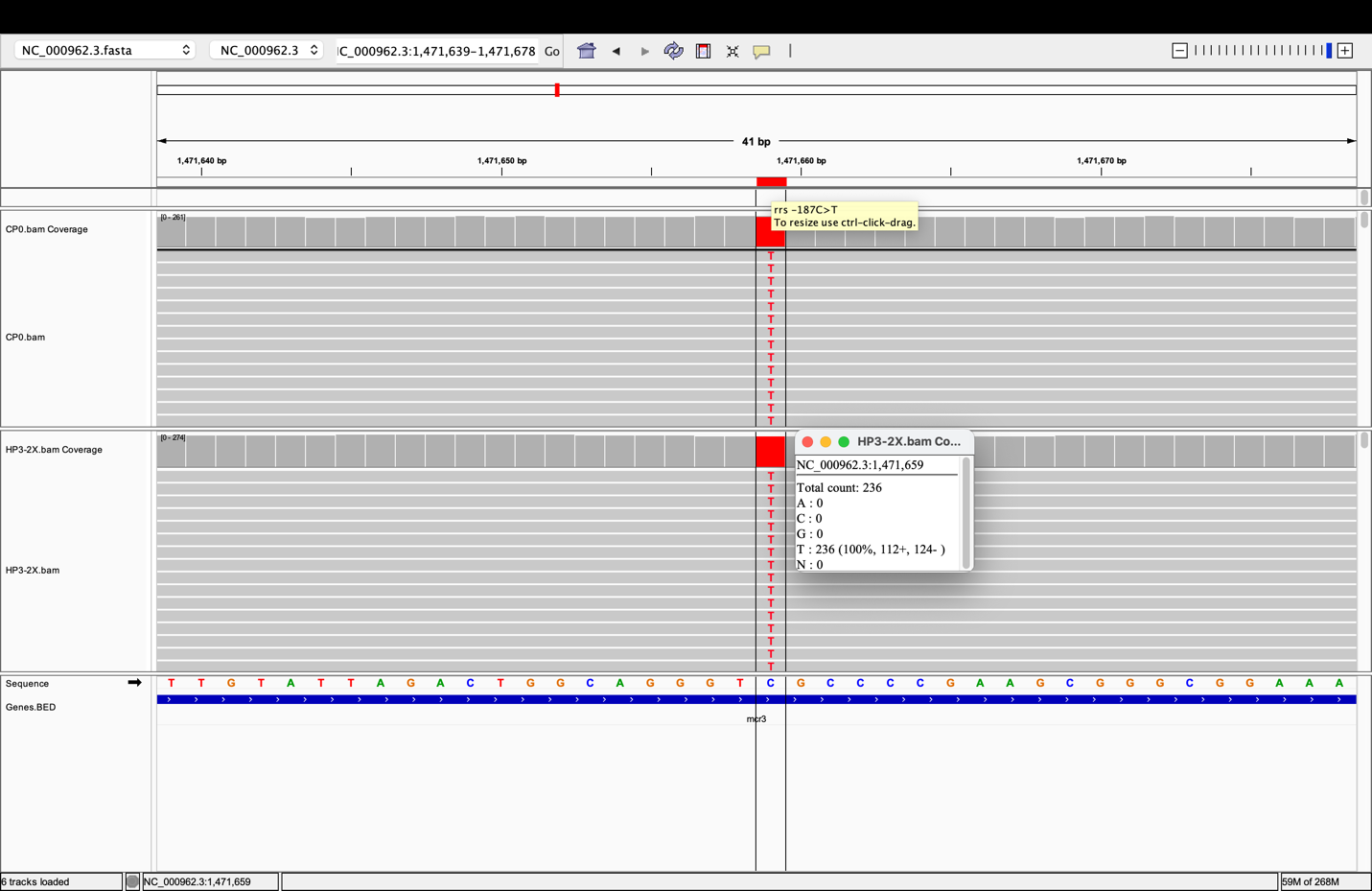


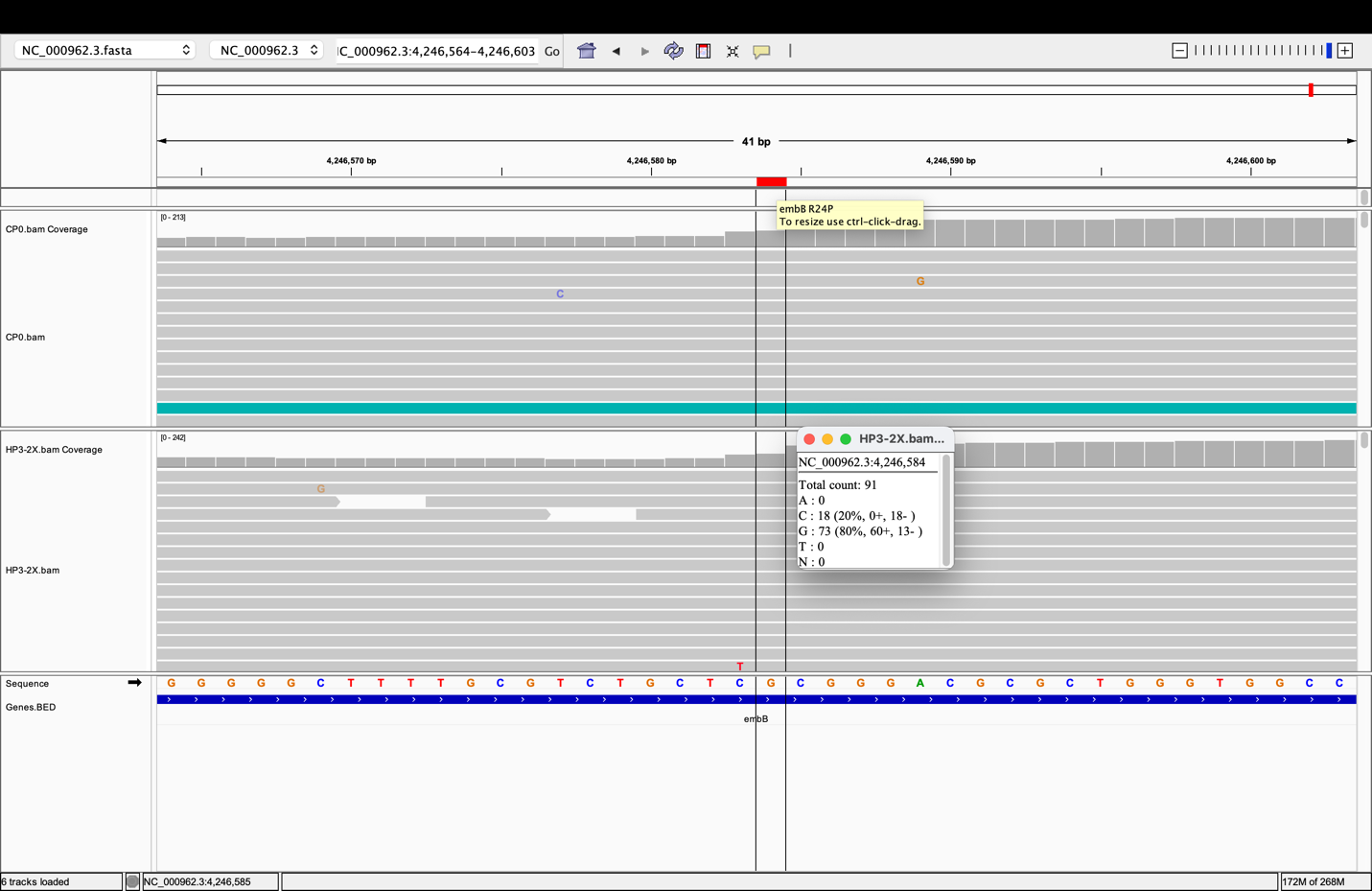

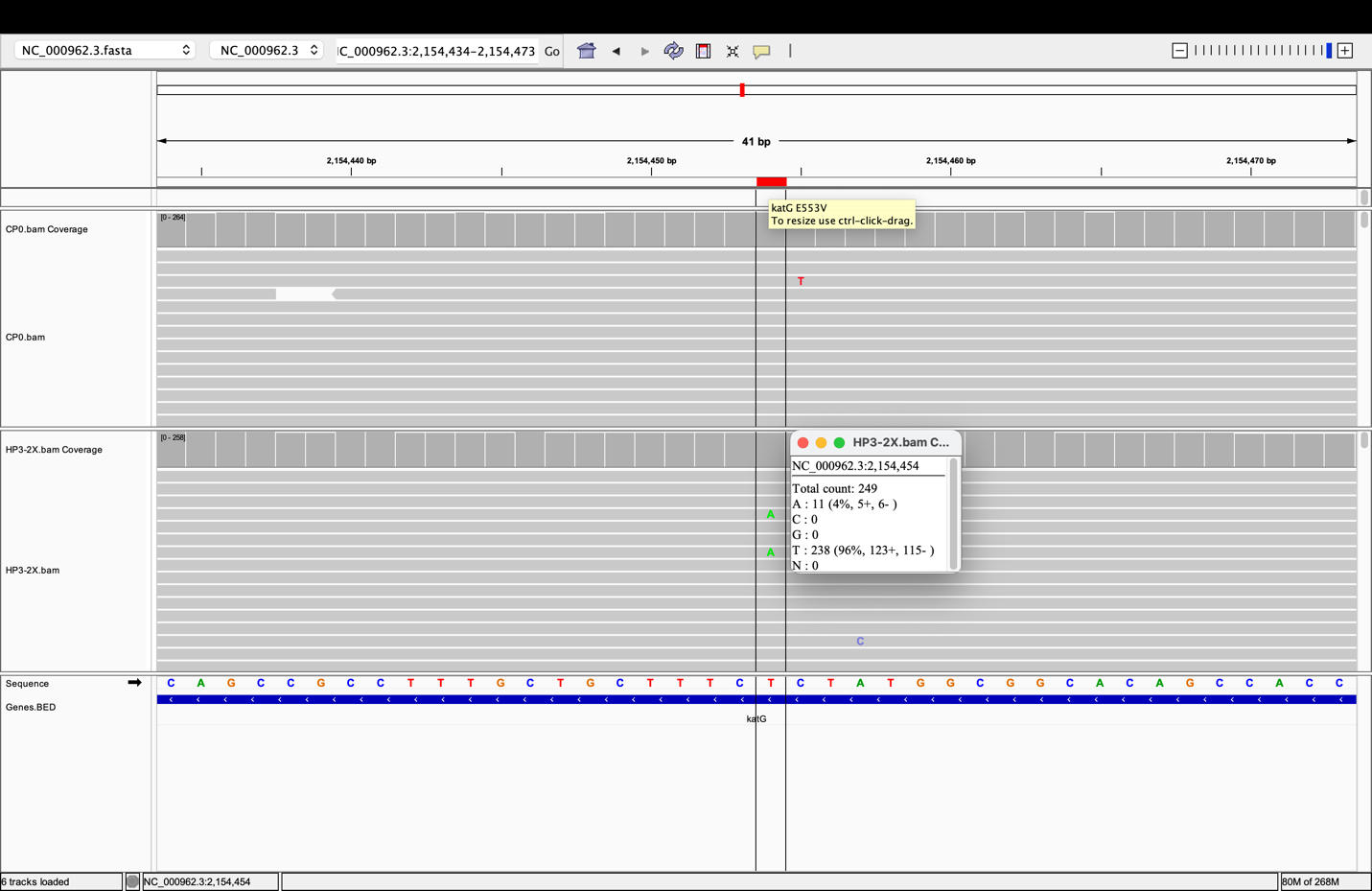


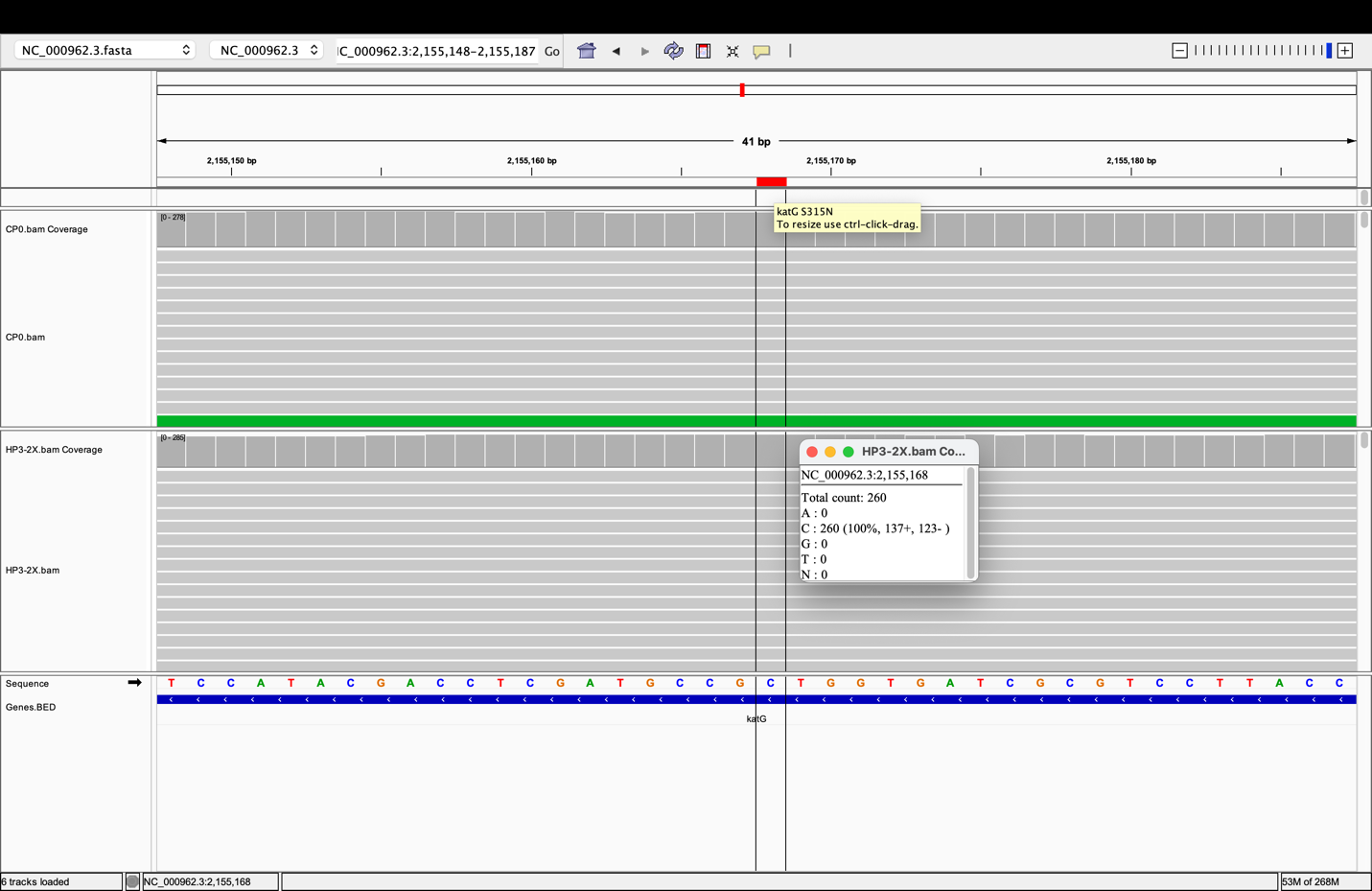

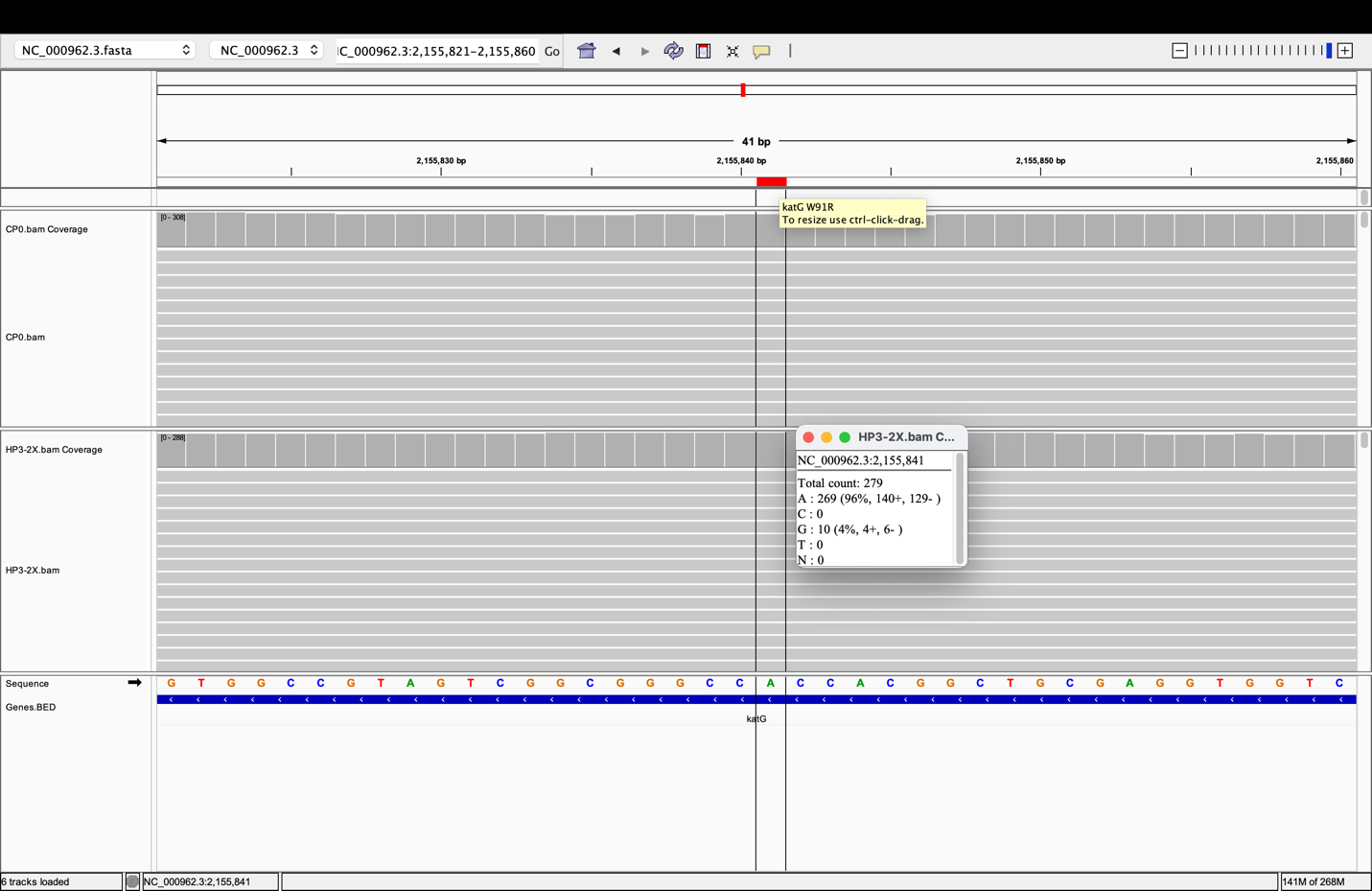


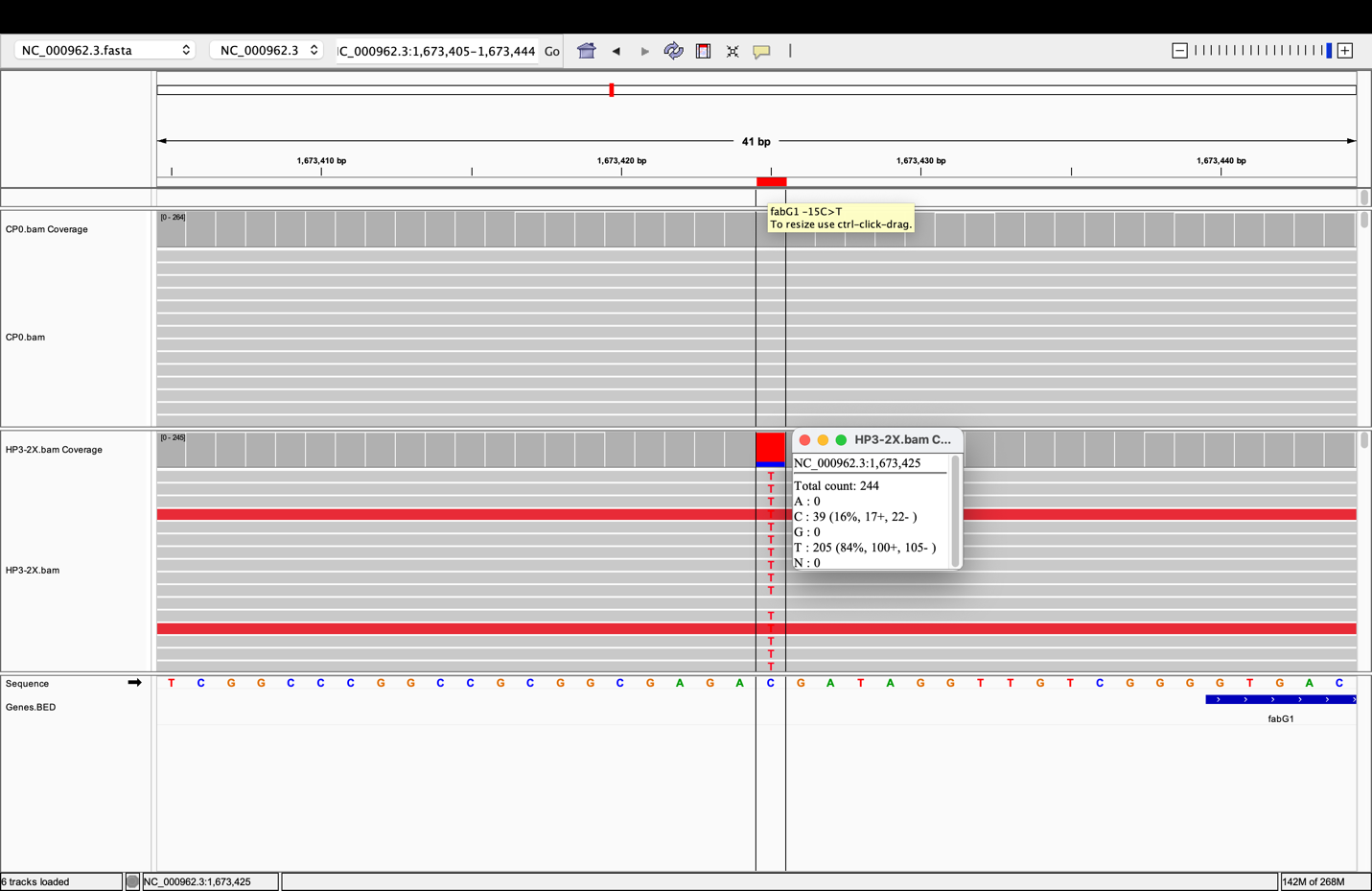

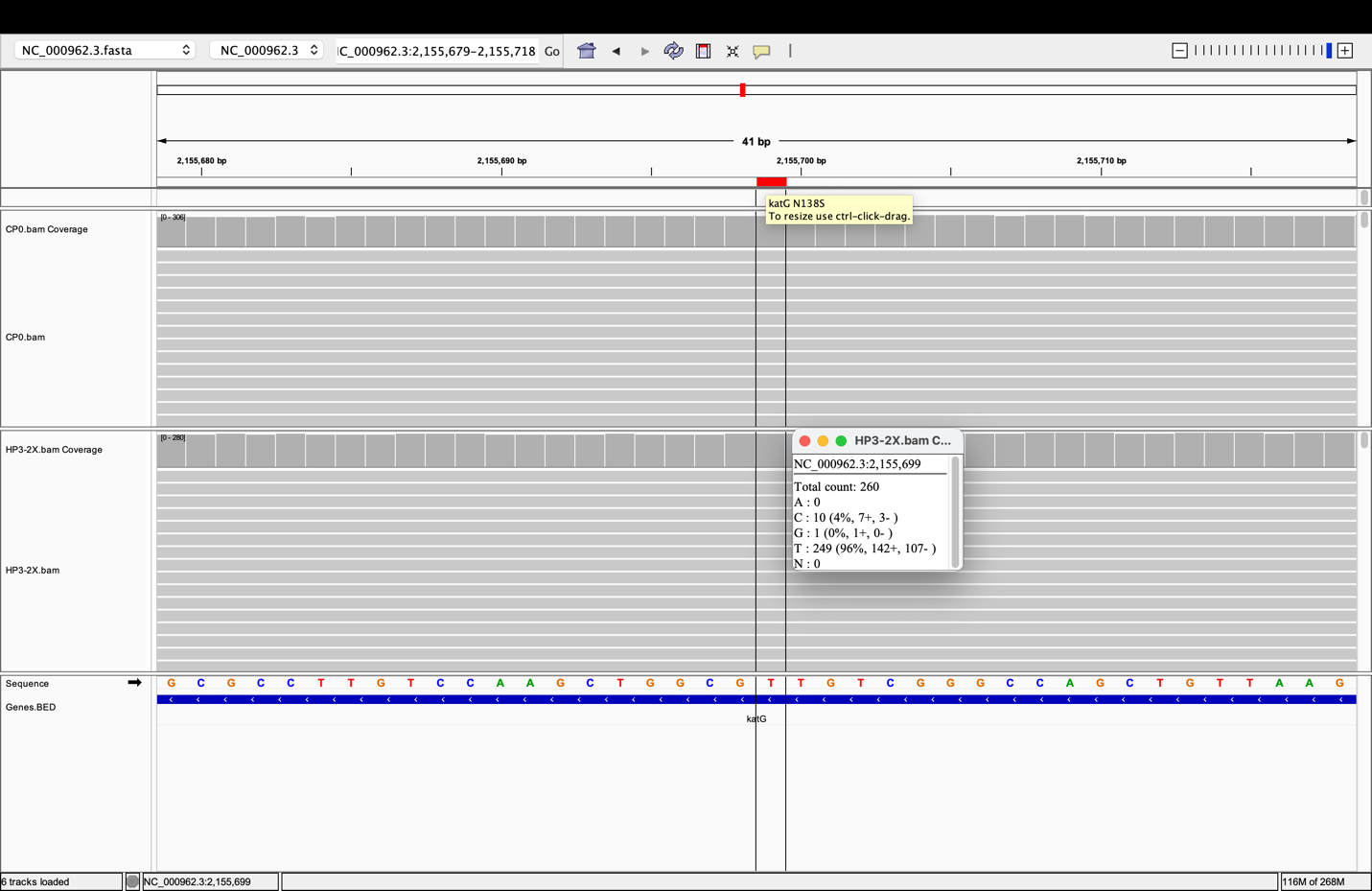


**Passage 4**

| **Passage 4 (2x Critical concentration)** | |  |  |  |  |
| --- | --- | --- | --- | --- | --- |
| **Variants detected** | **Estimated fraction (%)** |  |  |  |  |
| *mshA* A254G | 29 |  |  |  |  |
| *rrs* -187C>T | 100 |  |  |  |  |
| *rrs* -60T>G | 15 |  |  |  |  |
| embB R24P | 16 |  |  |  |  |
| *katG* E553V | 5 |  |  |  |  |
| *katG* W91R | 6 |  |  |  |  |
| *katG* S315N | 0 |  |  |  |  |
| *katG* N138S | 47 |  |  |  |  |
| *fabG1* -15C>T | 13 |  |  |  |  |
|  |  |  |  |  |  |
| **The number estimated fractions might not same as all IGV screenshots because mostly retrieved from the TB-profiler reports, and some from IGV for the positions that were not reported by TB-profiler.* | | | | | |


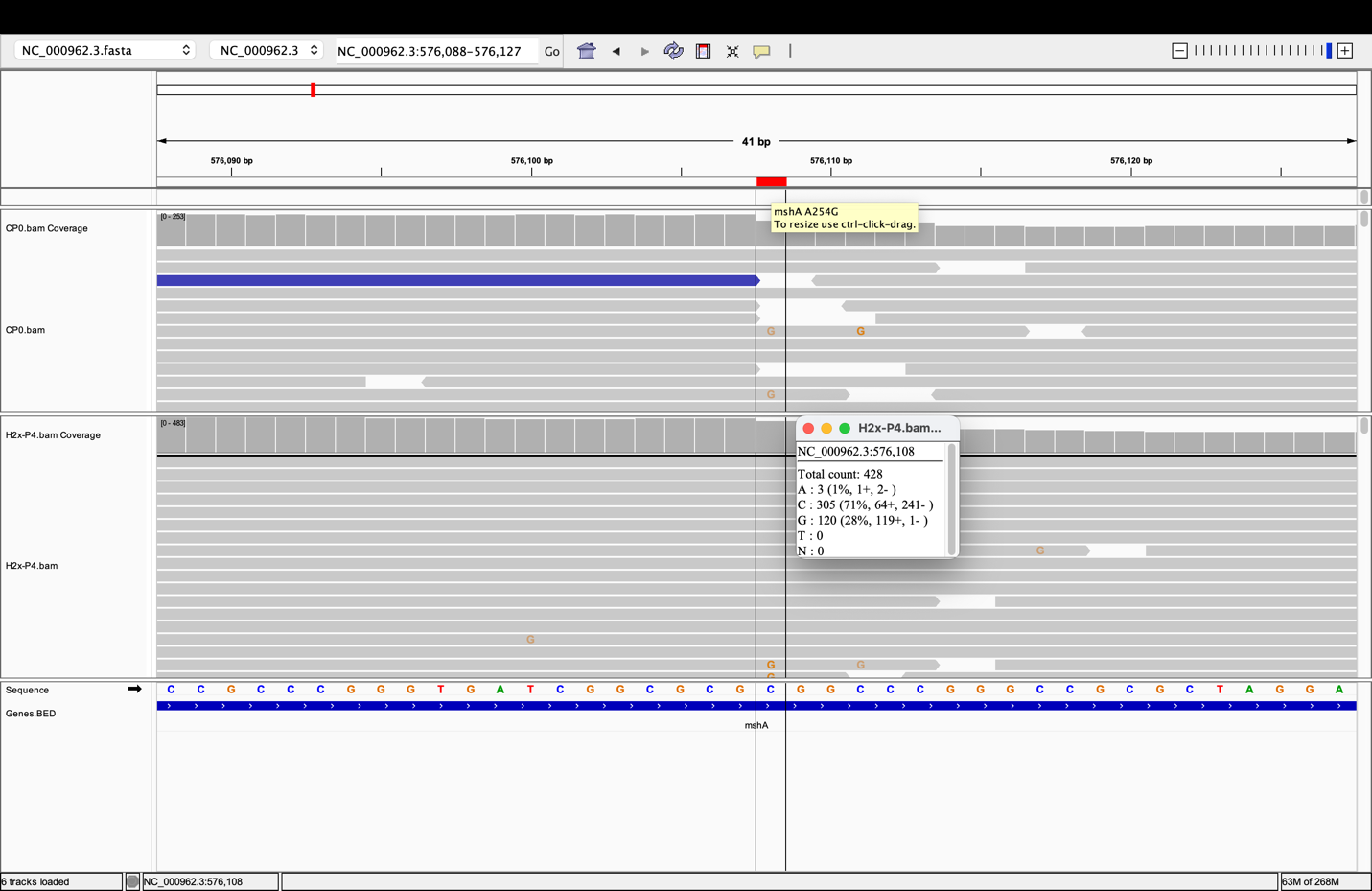


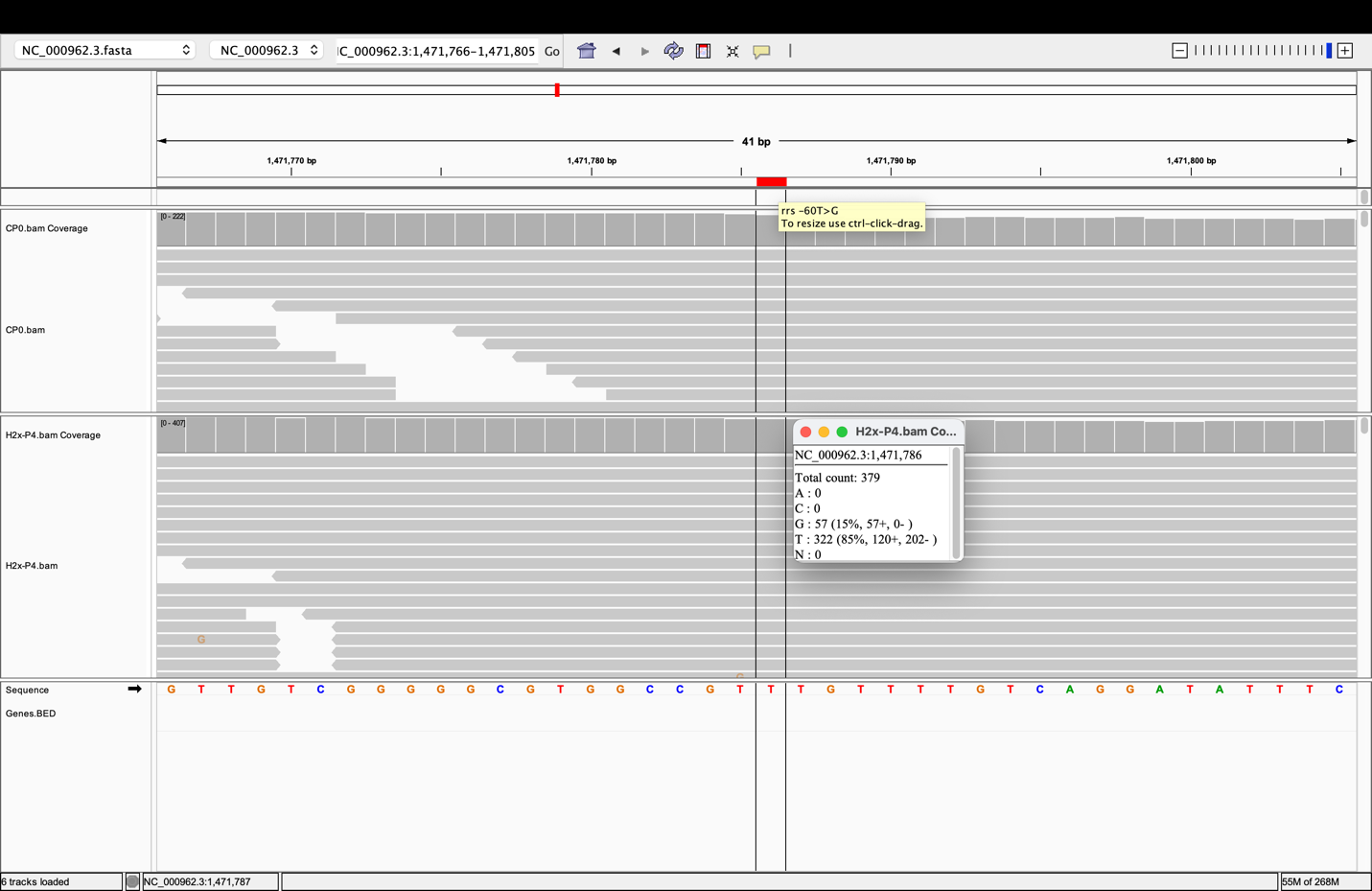

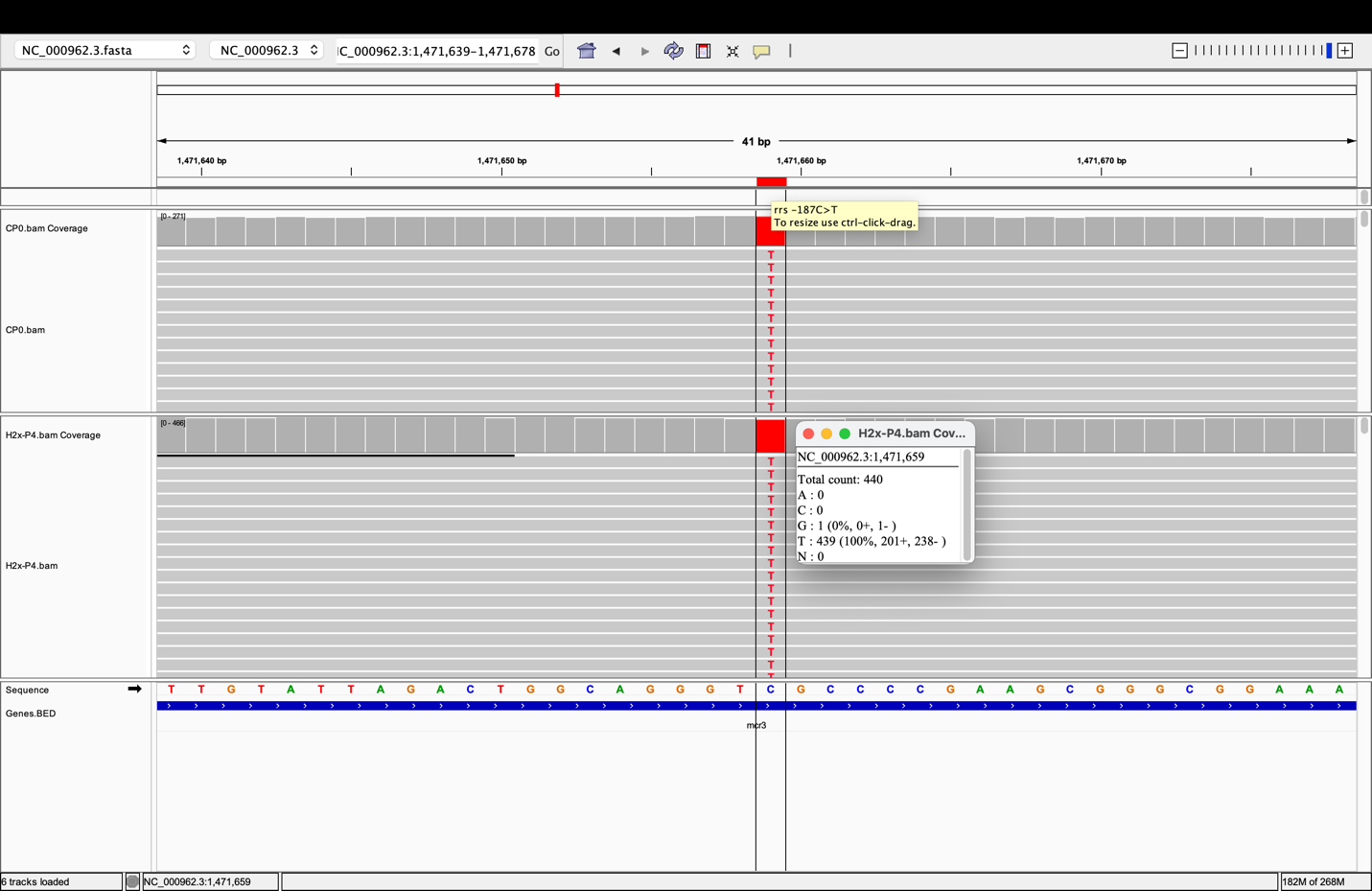


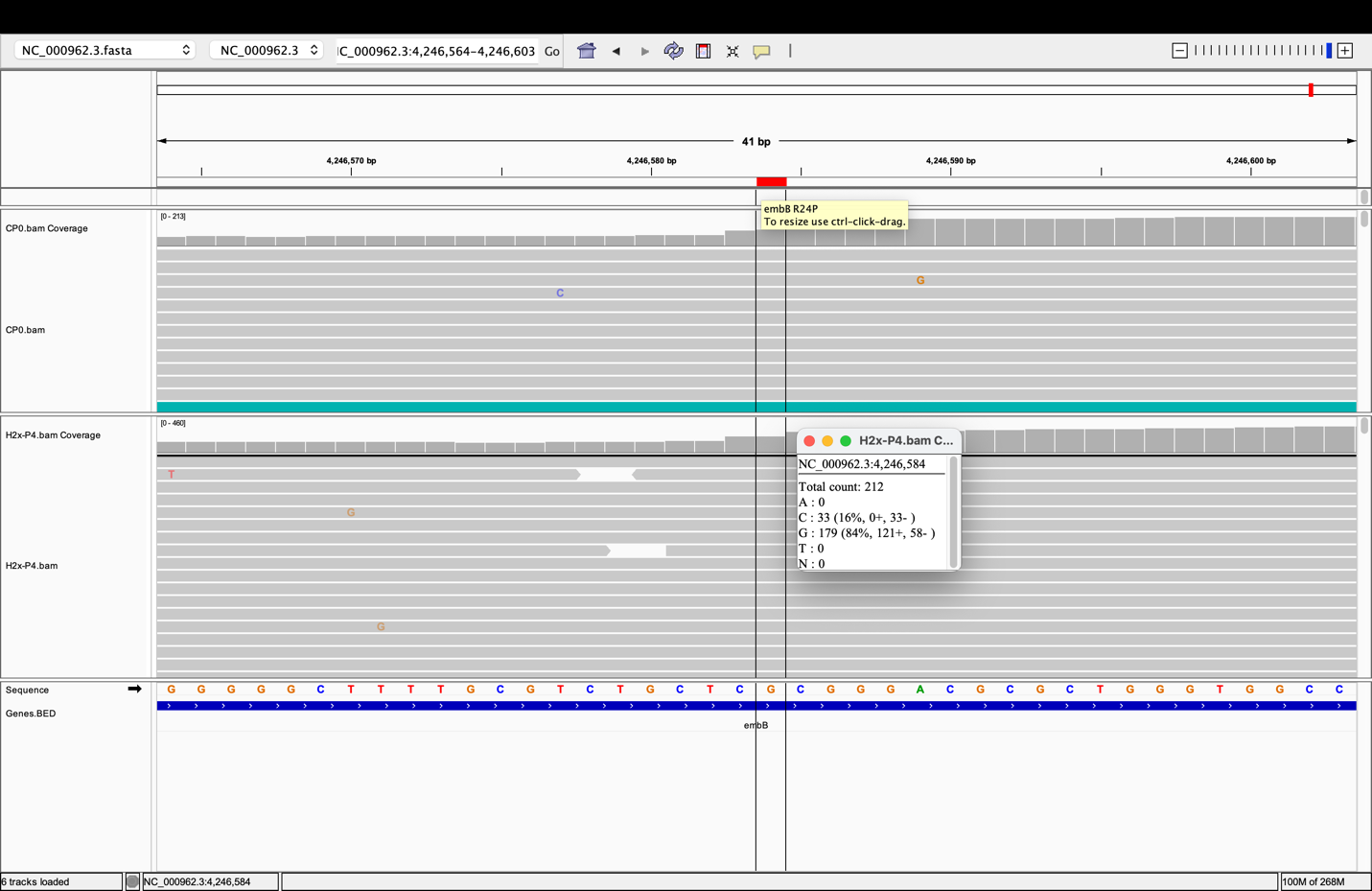

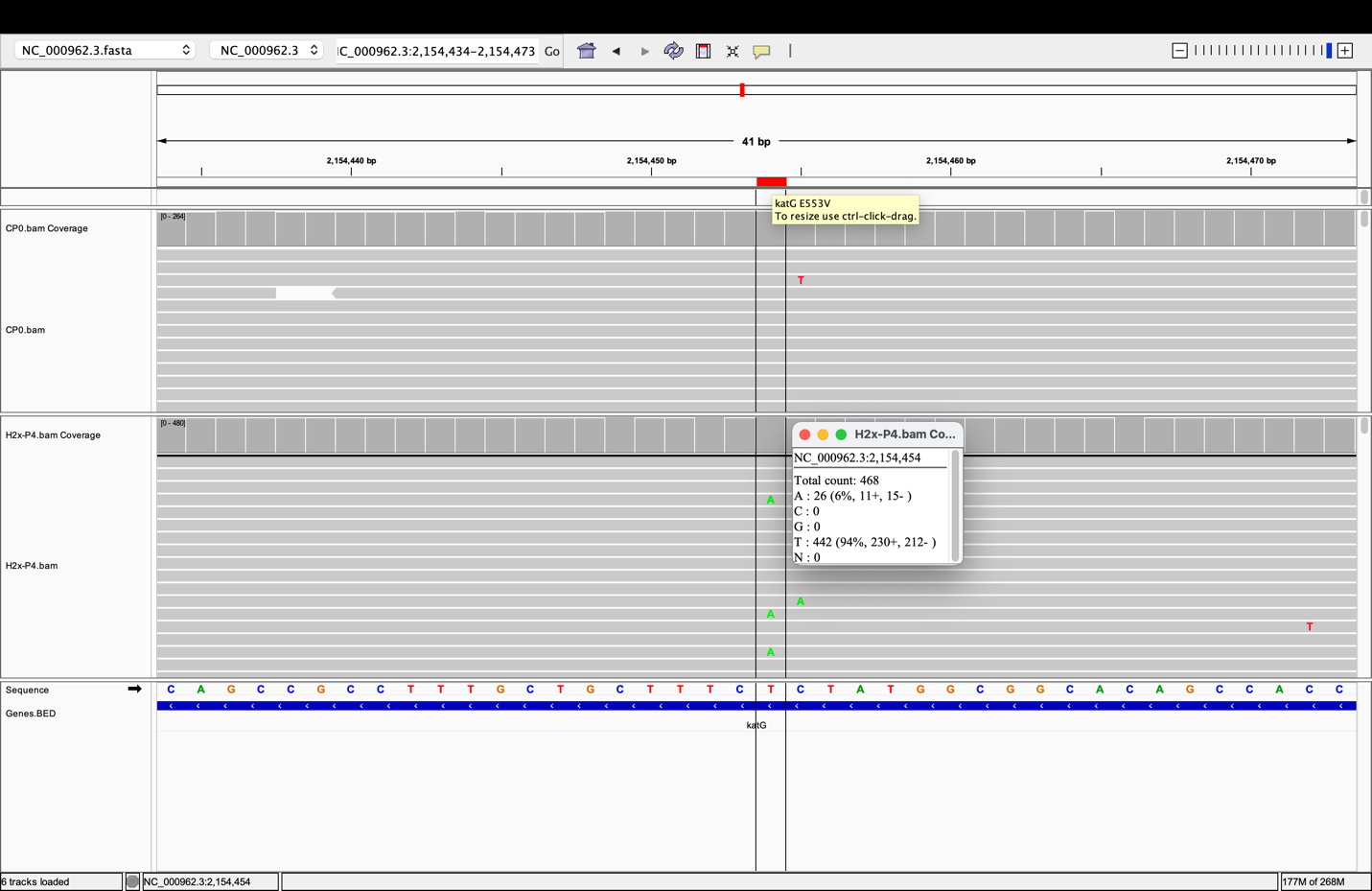


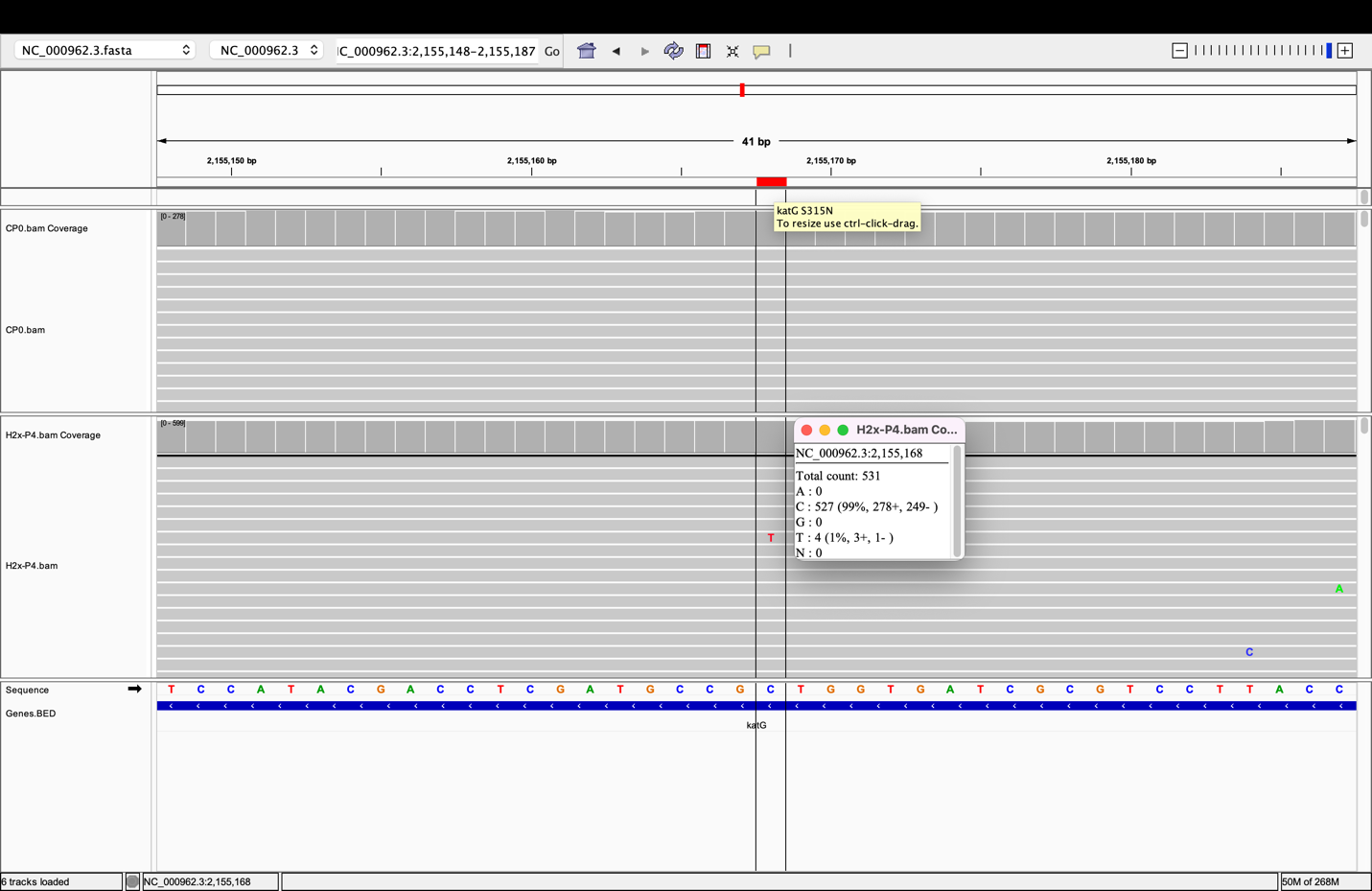

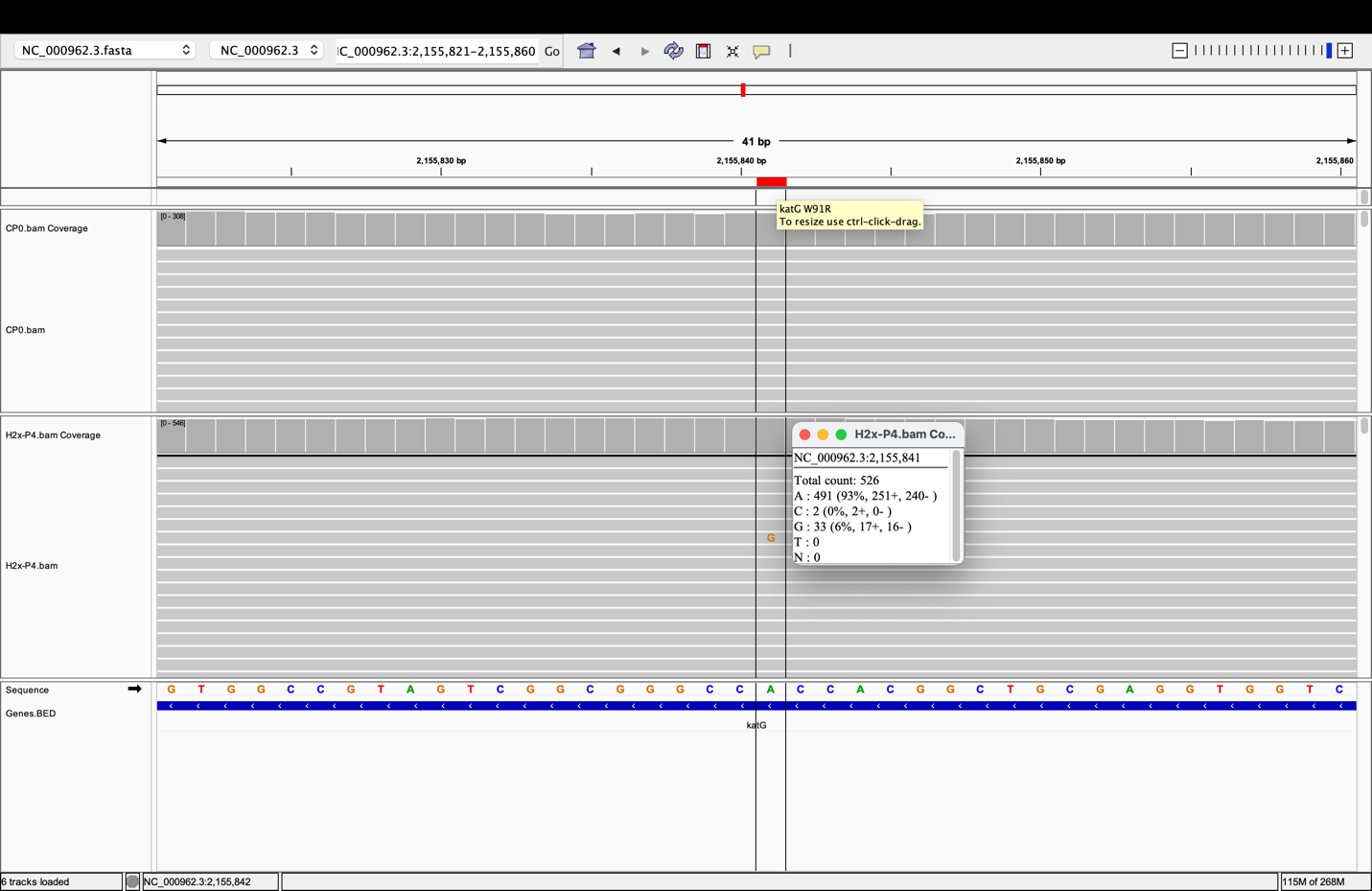


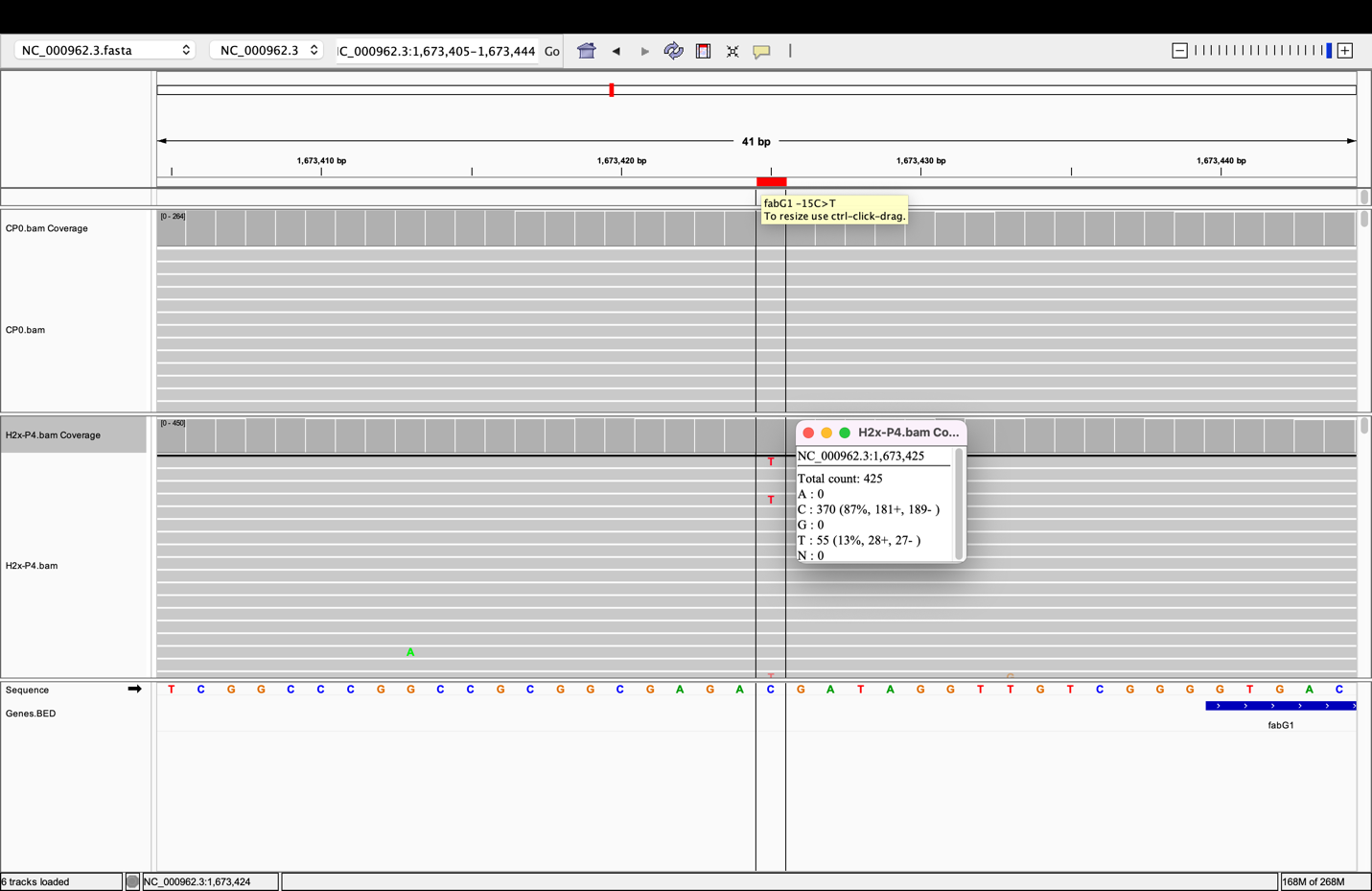

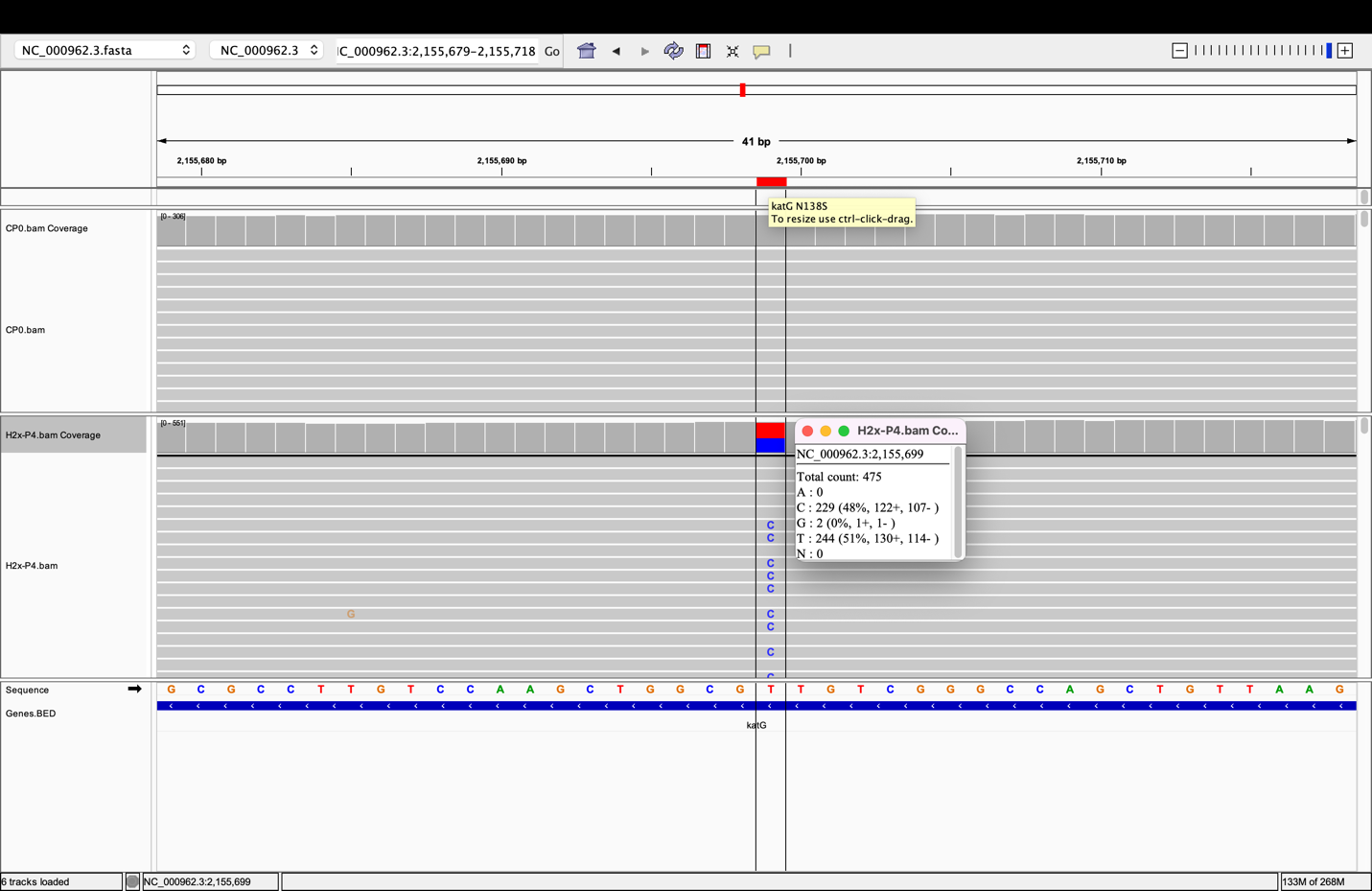


**Passage 5**

| **Passage 5 (4x Critical concentration)** | |  |  |  |  |
| --- | --- | --- | --- | --- | --- |
| **Variants detected** | **Estimated fraction (%)** |  |  |  |  |
| *mshA* A254G | 23 |  |  |  |  |
| *rrs* -187C>T | 100 |  |  |  |  |
| *rrs* -60T>G | 11 |  |  |  |  |
| *rrl* 2904C>T | 30 |  |  |  |  |
| embB R24P | 16 |  |  |  |  |
| *katG* E553V | 0 |  |  |  |  |
| *katG* W91R | 0 |  |  |  |  |
| *katG* S315N | 12 |  |  |  |  |
| *katG* N138S | 41 |  |  |  |  |
| *fabG1* -15C>T | 0 |  |  |  |  |
| *katG* A162E | 18 |  |  |  |  |
| *katG* K414N | 39 |  |  |  |  |
| *ahpC* -72C>T | 38 |  |  |  |  |
| *ahpC* 21C>A | 15 |  |  |  |  |
|  |  |  |  |  |  |
| **The number estimated fractions might not same as all IGV screenshots because mostly retrieved from the TB-profiler reports, and some from IGV for the positions that were not reported by TB-profiler.* | | | | | |


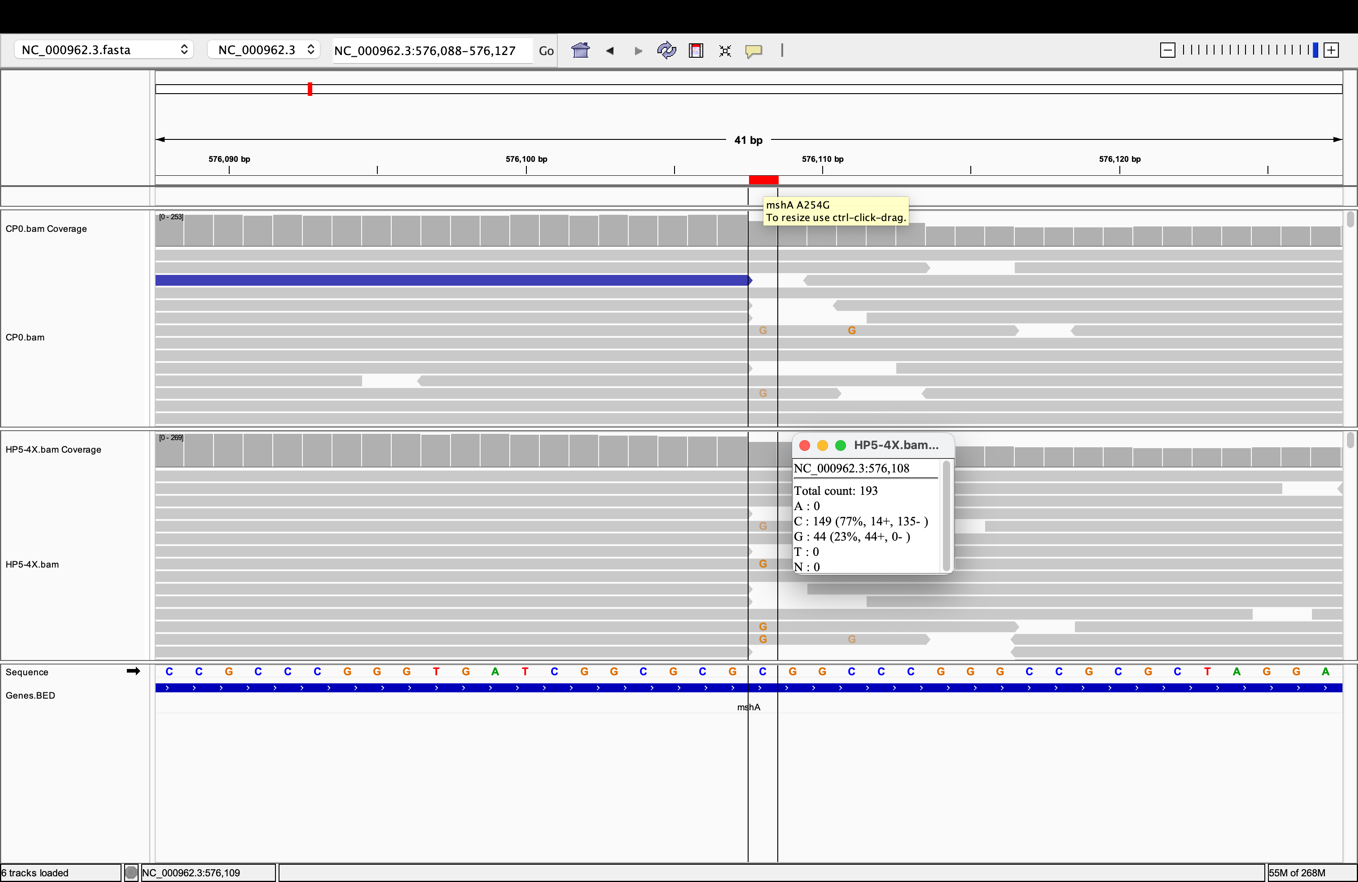


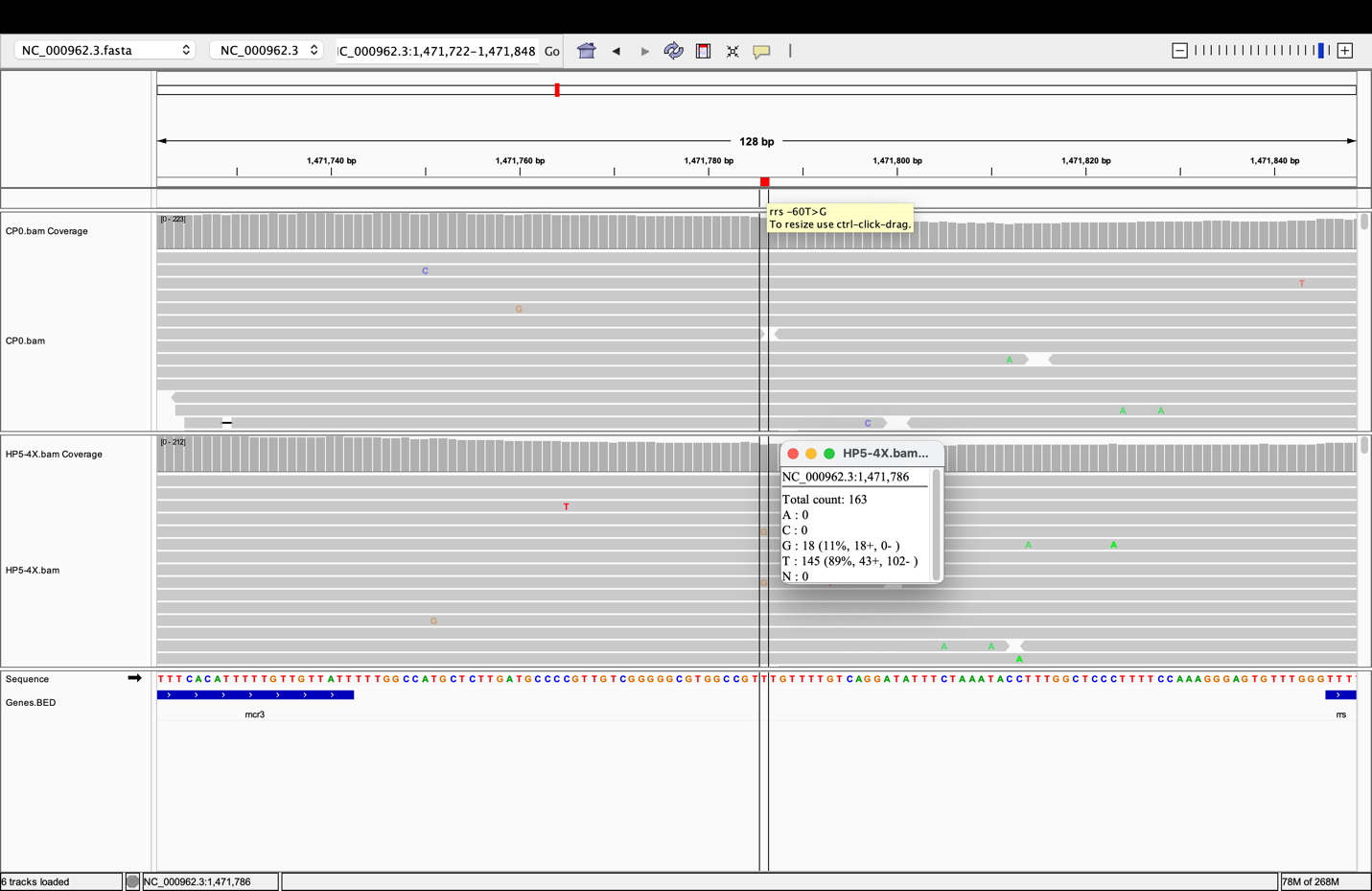

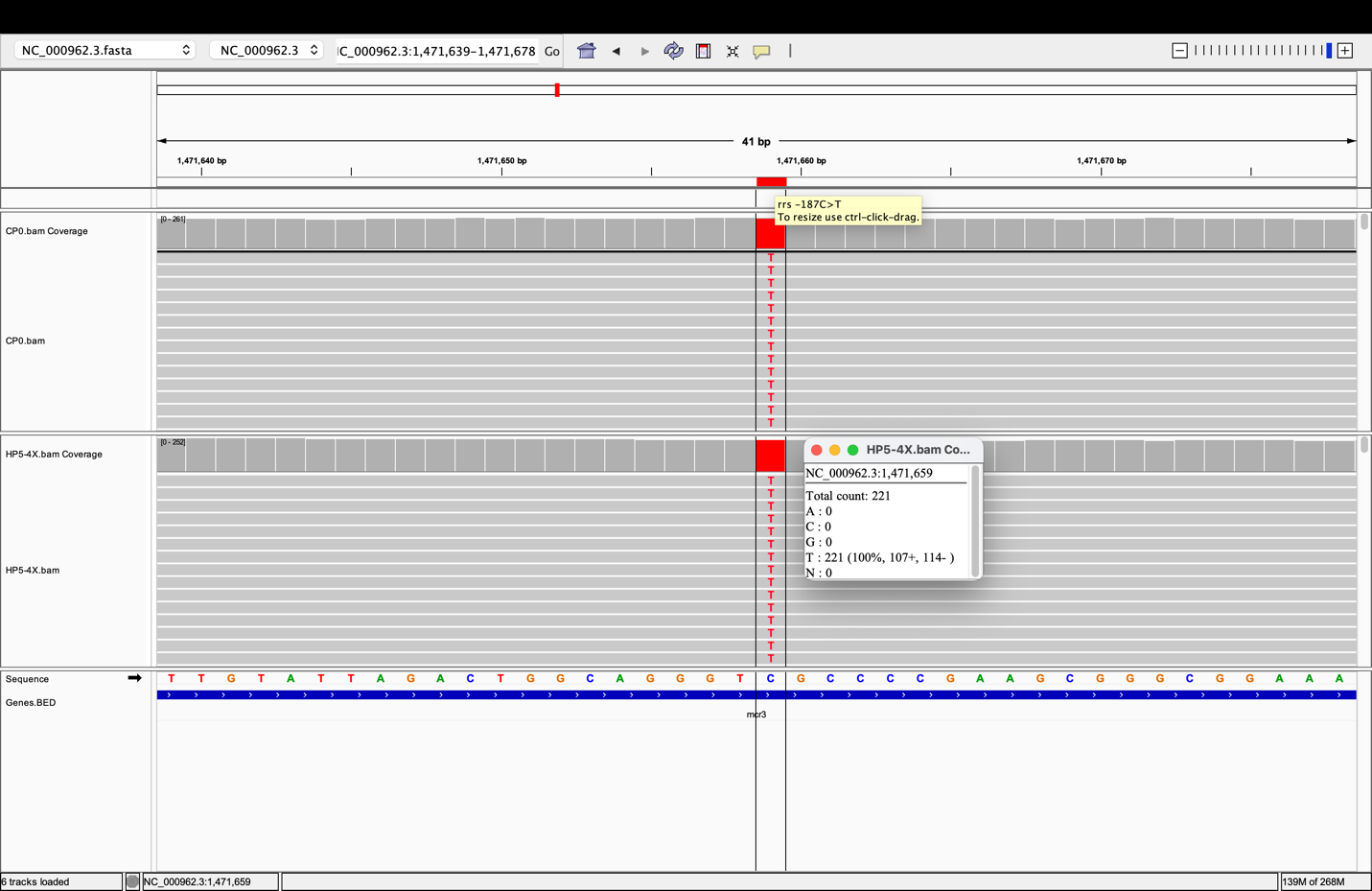


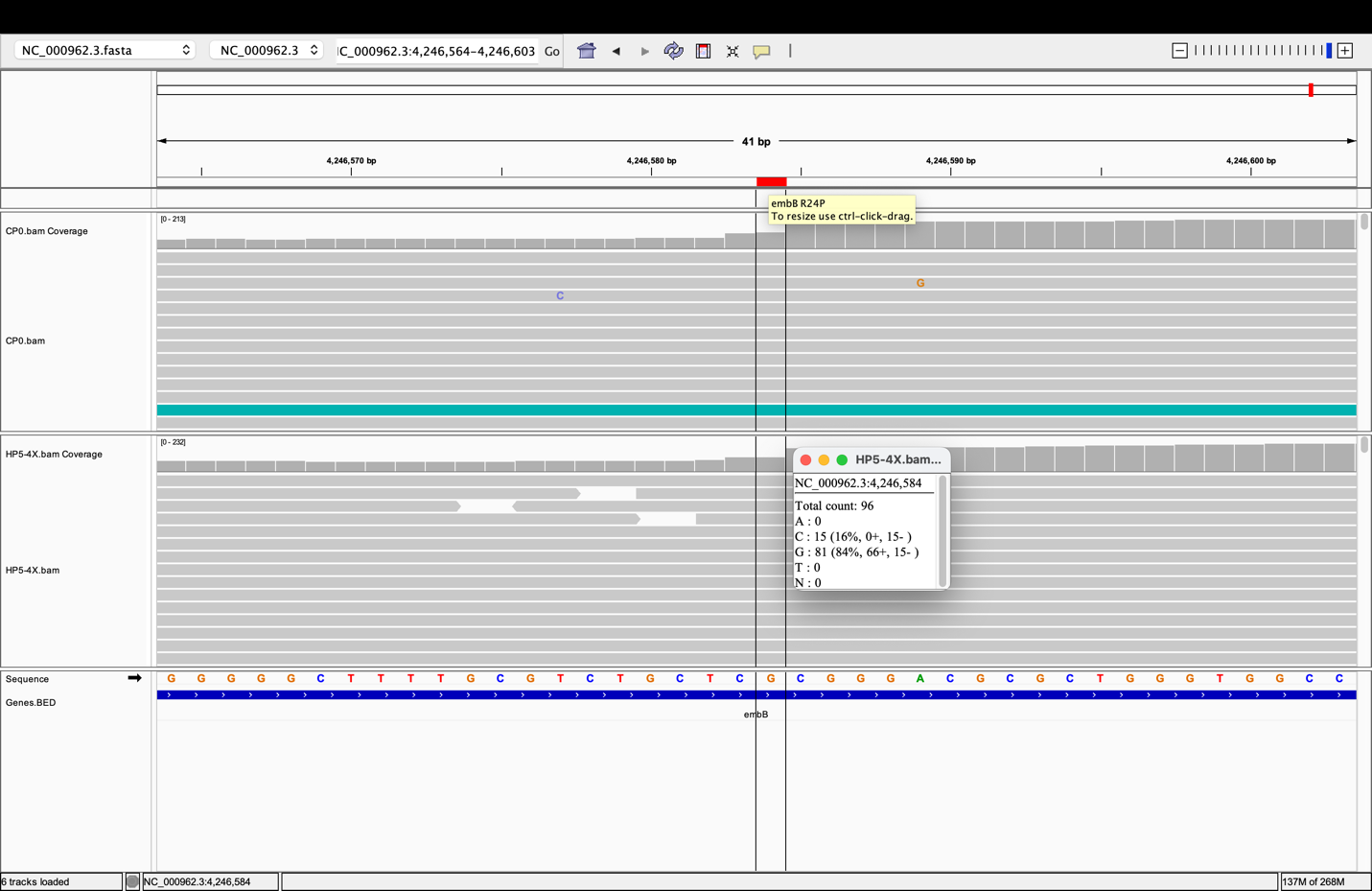

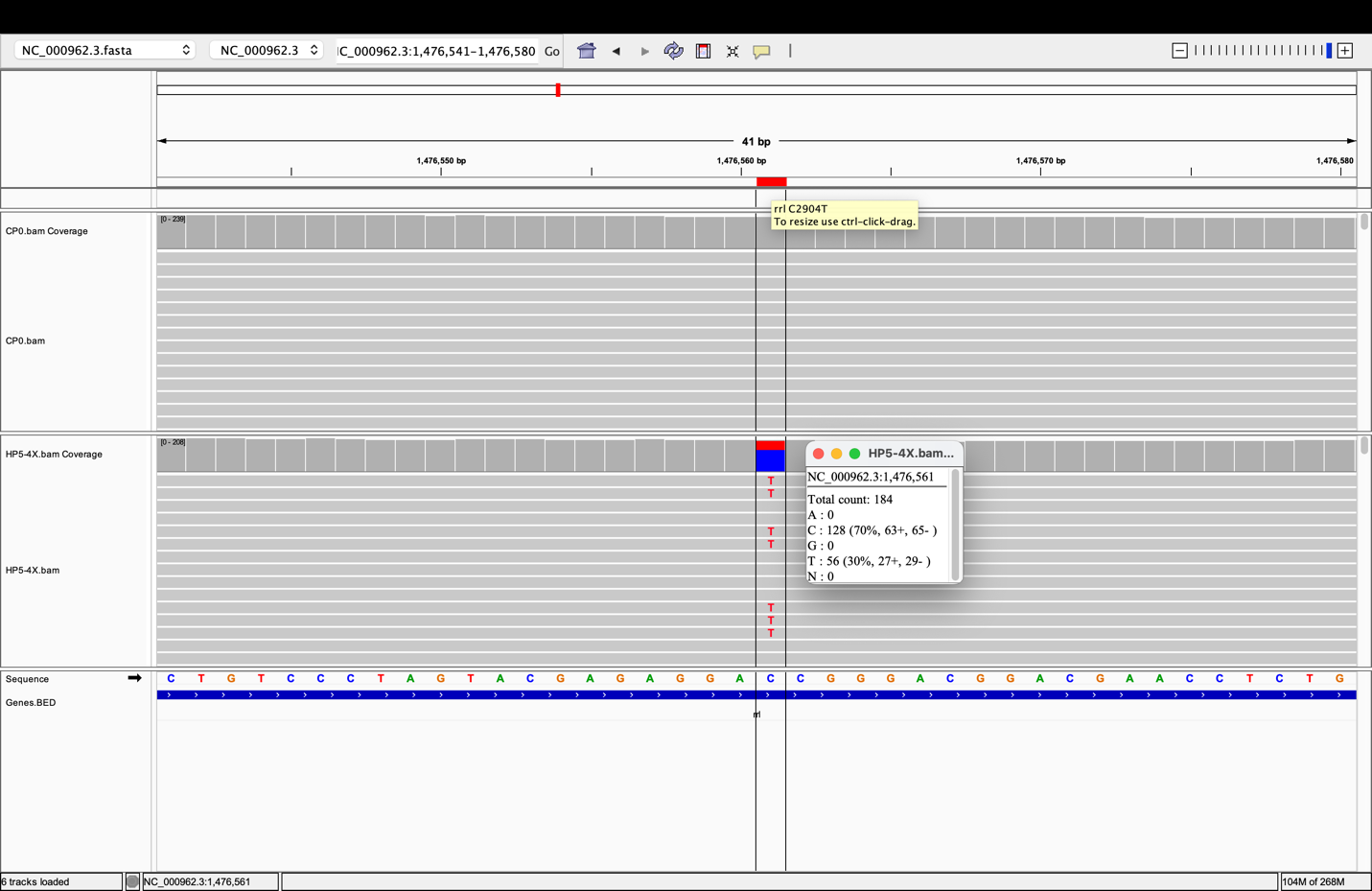


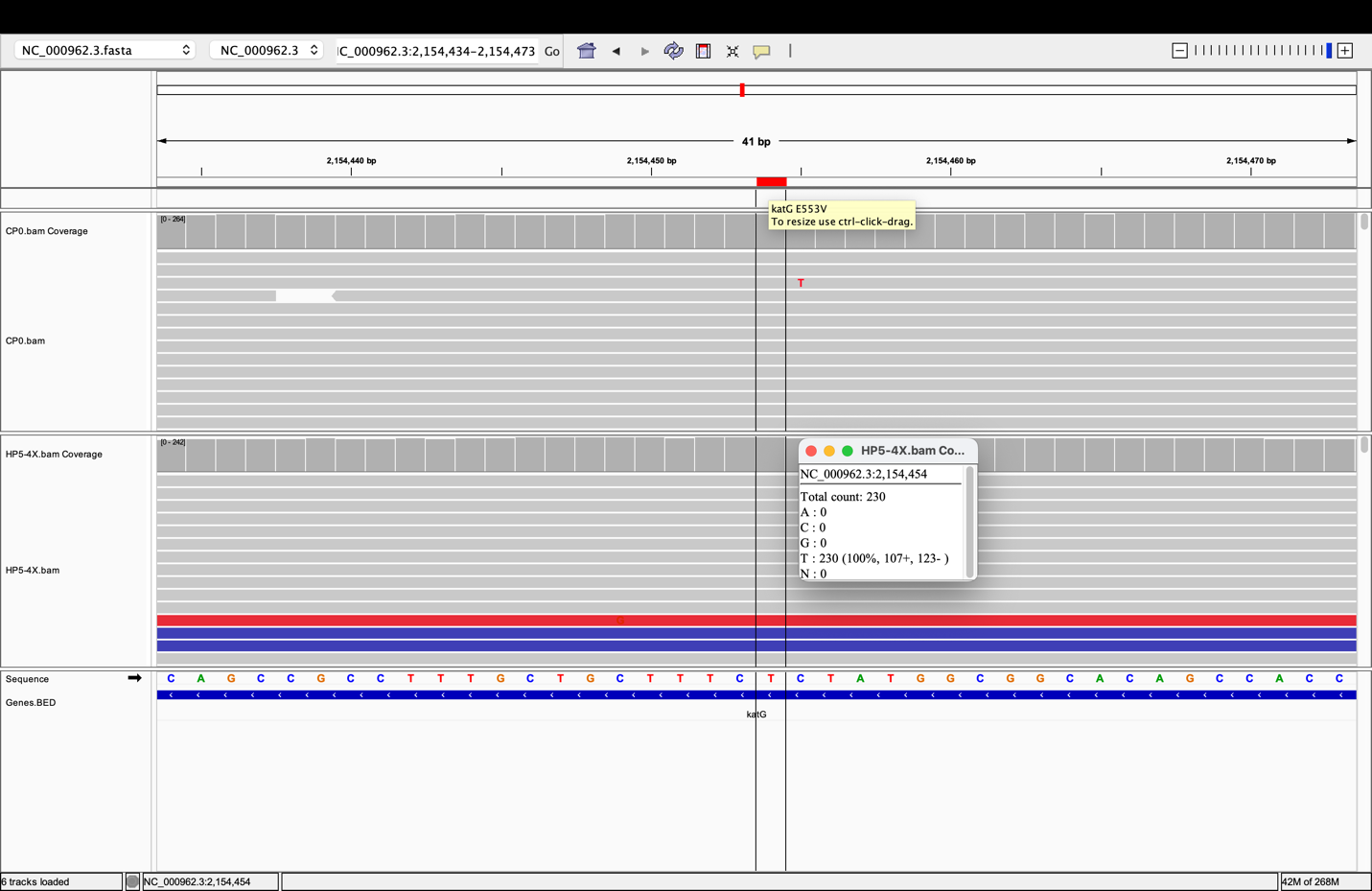

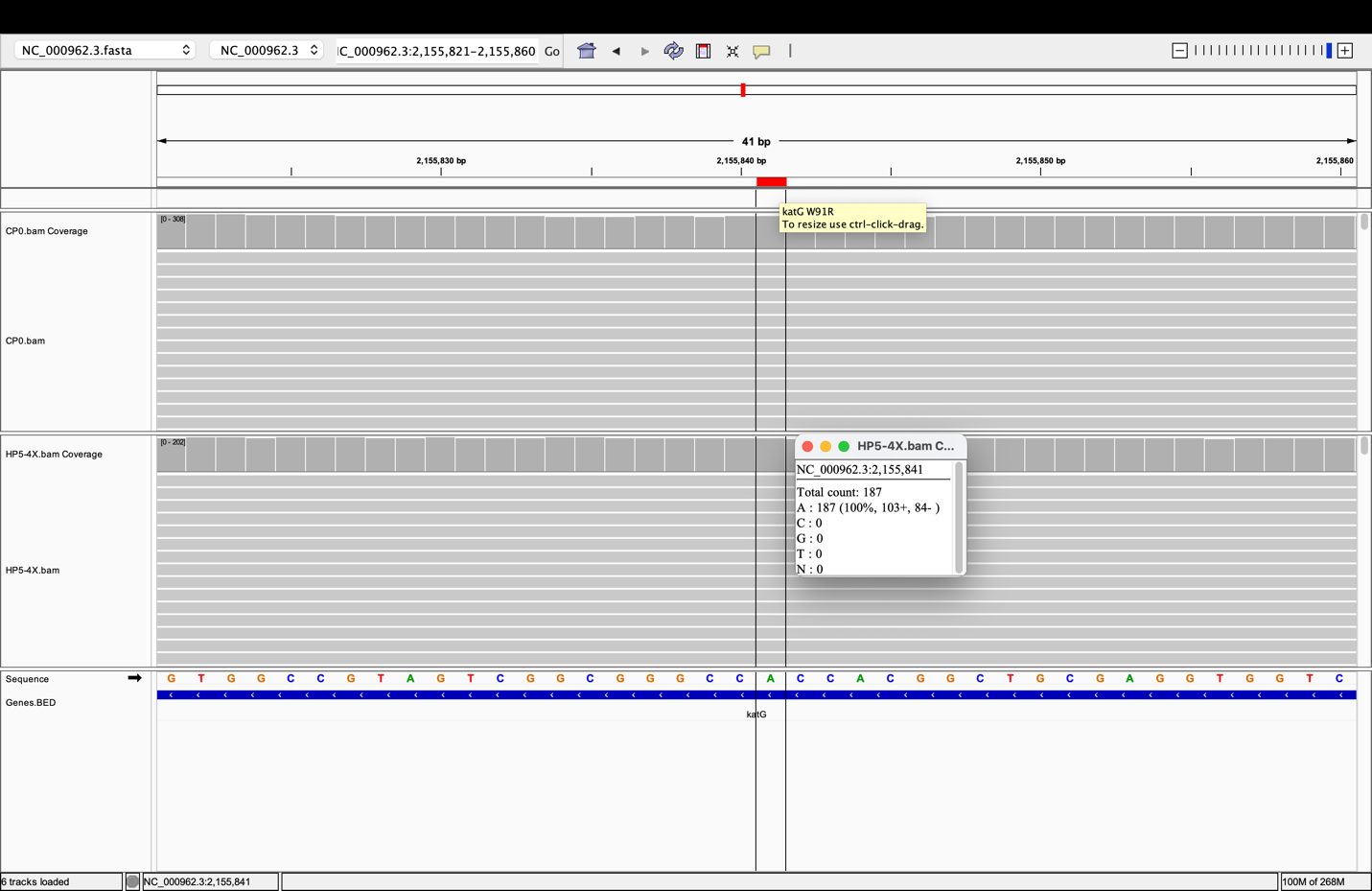


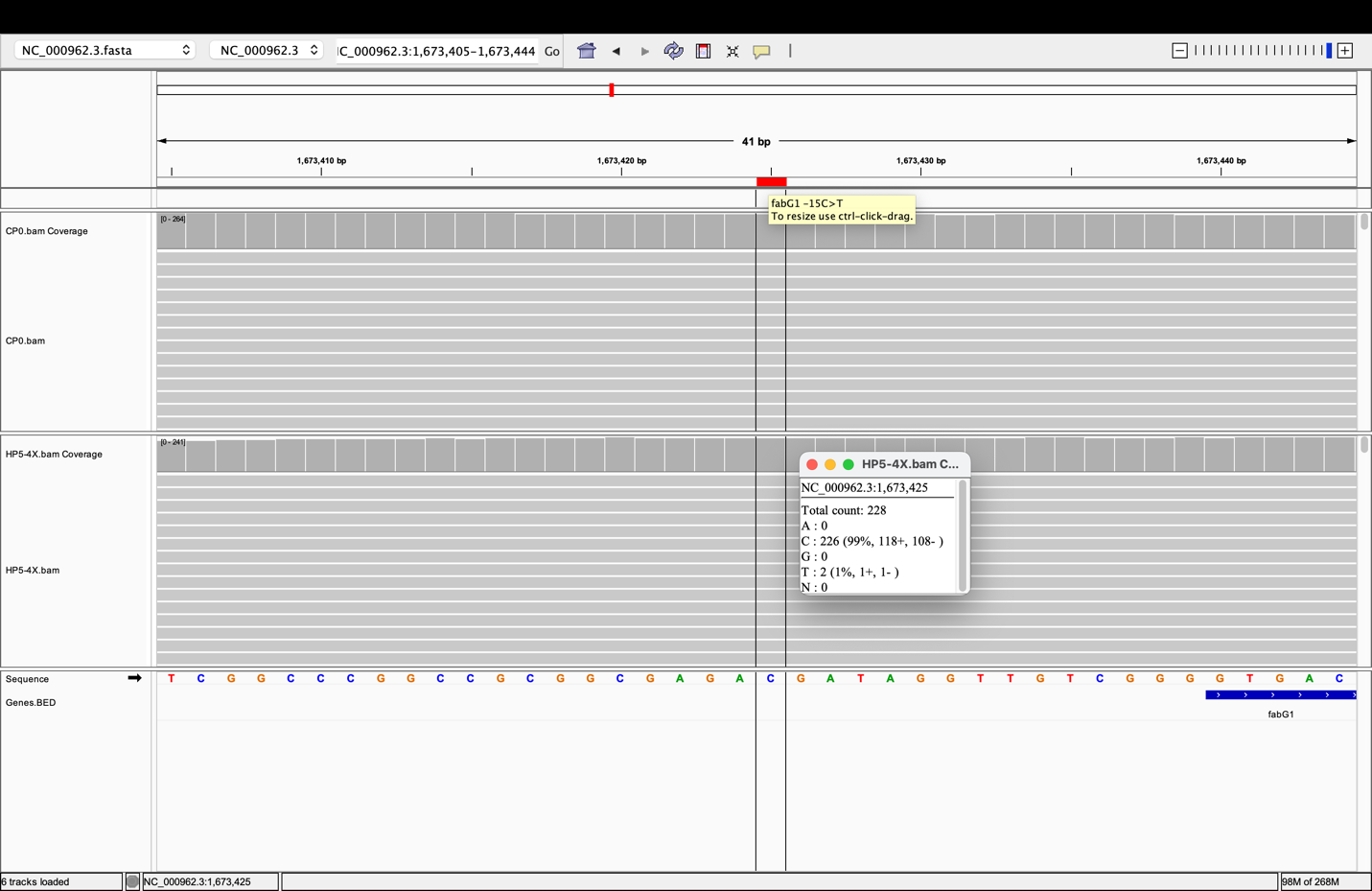

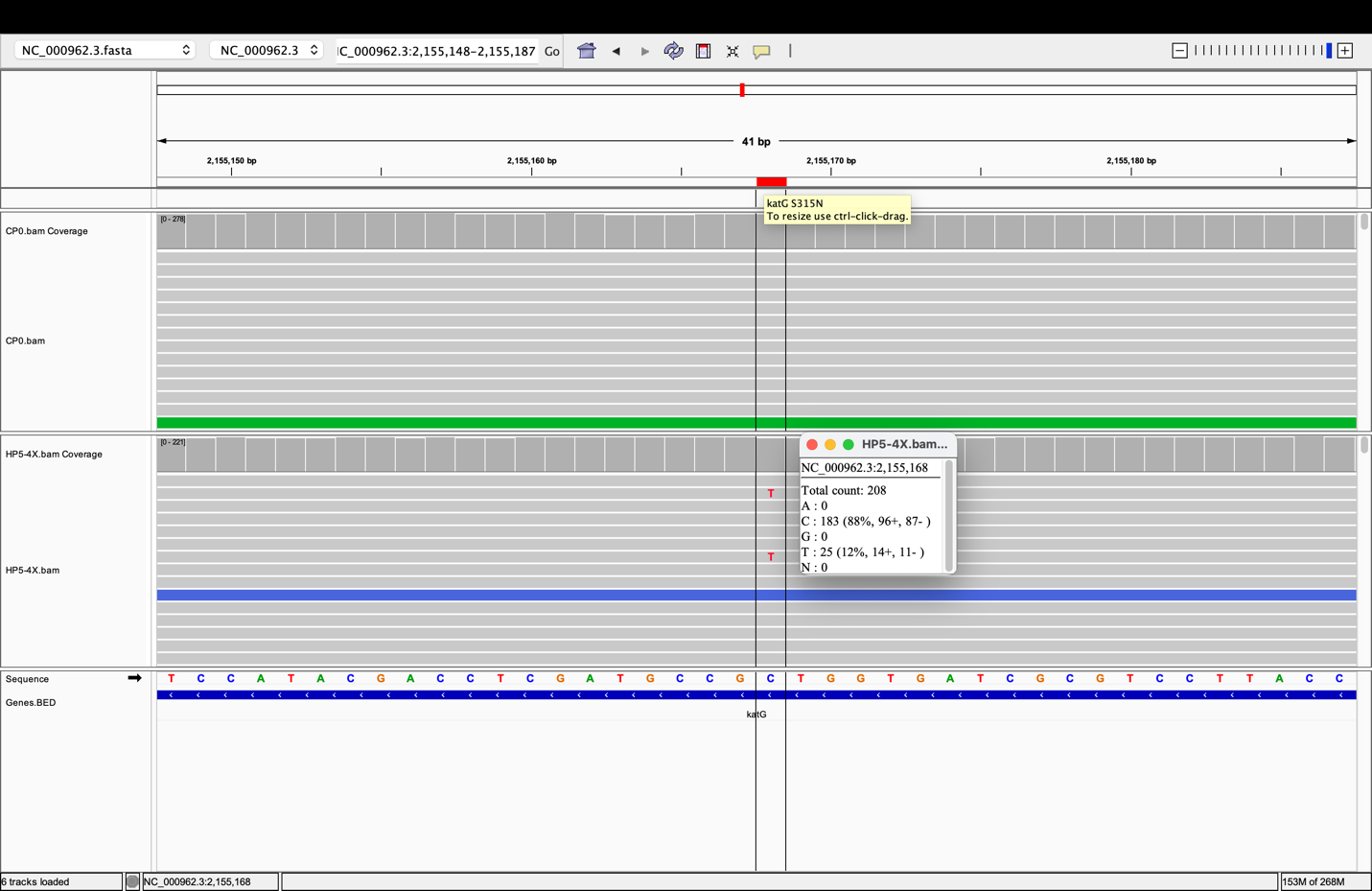


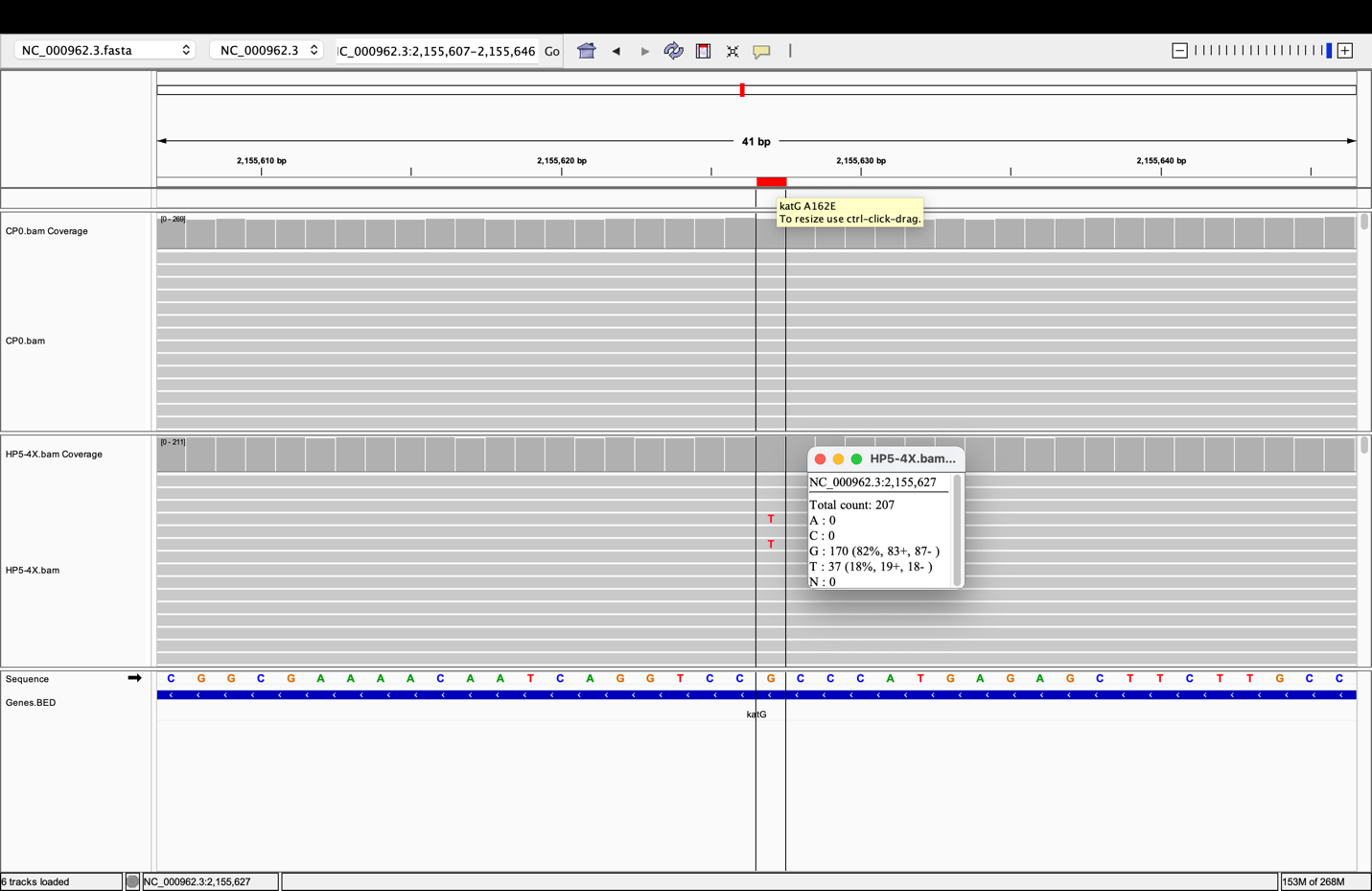

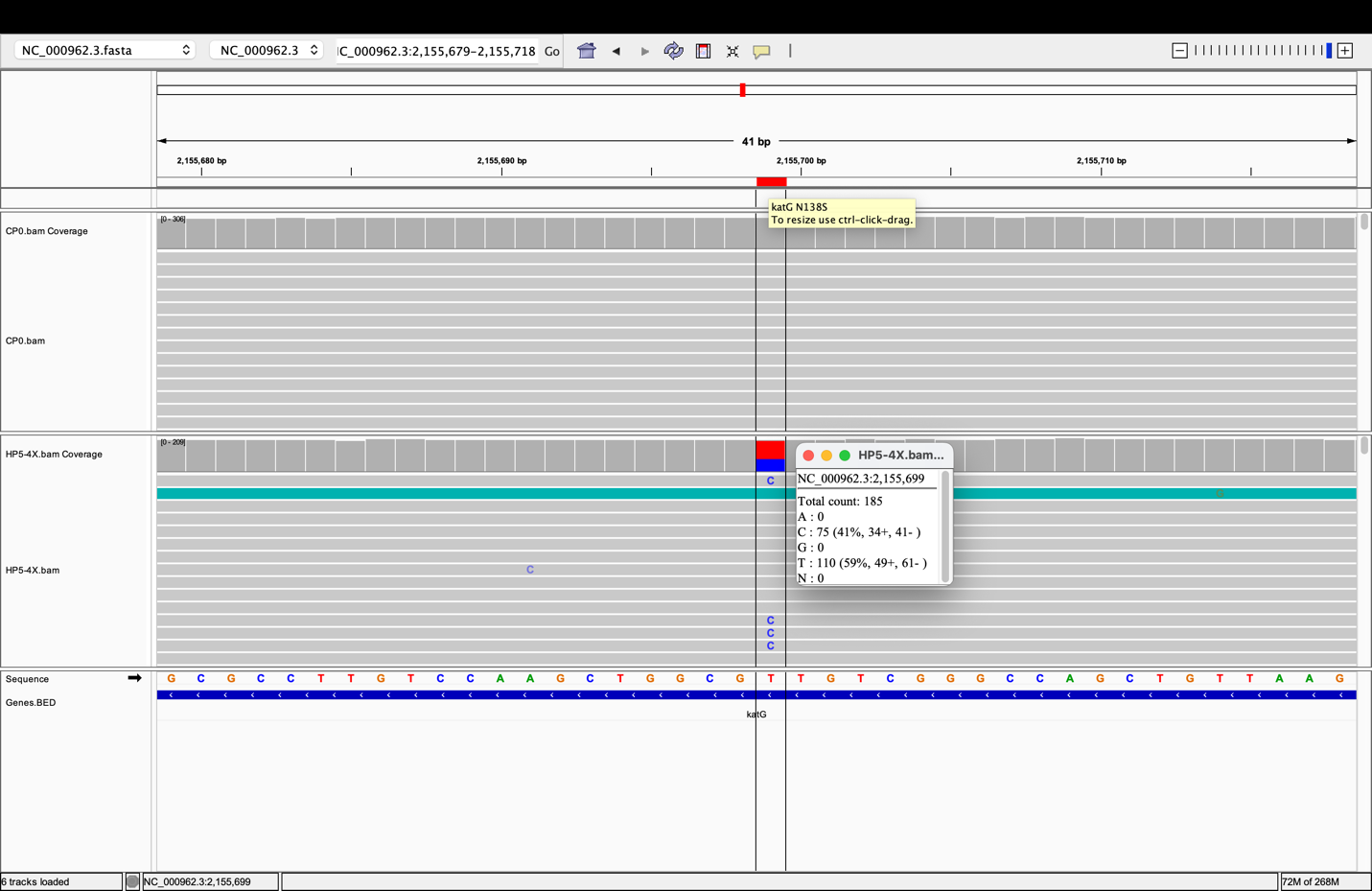


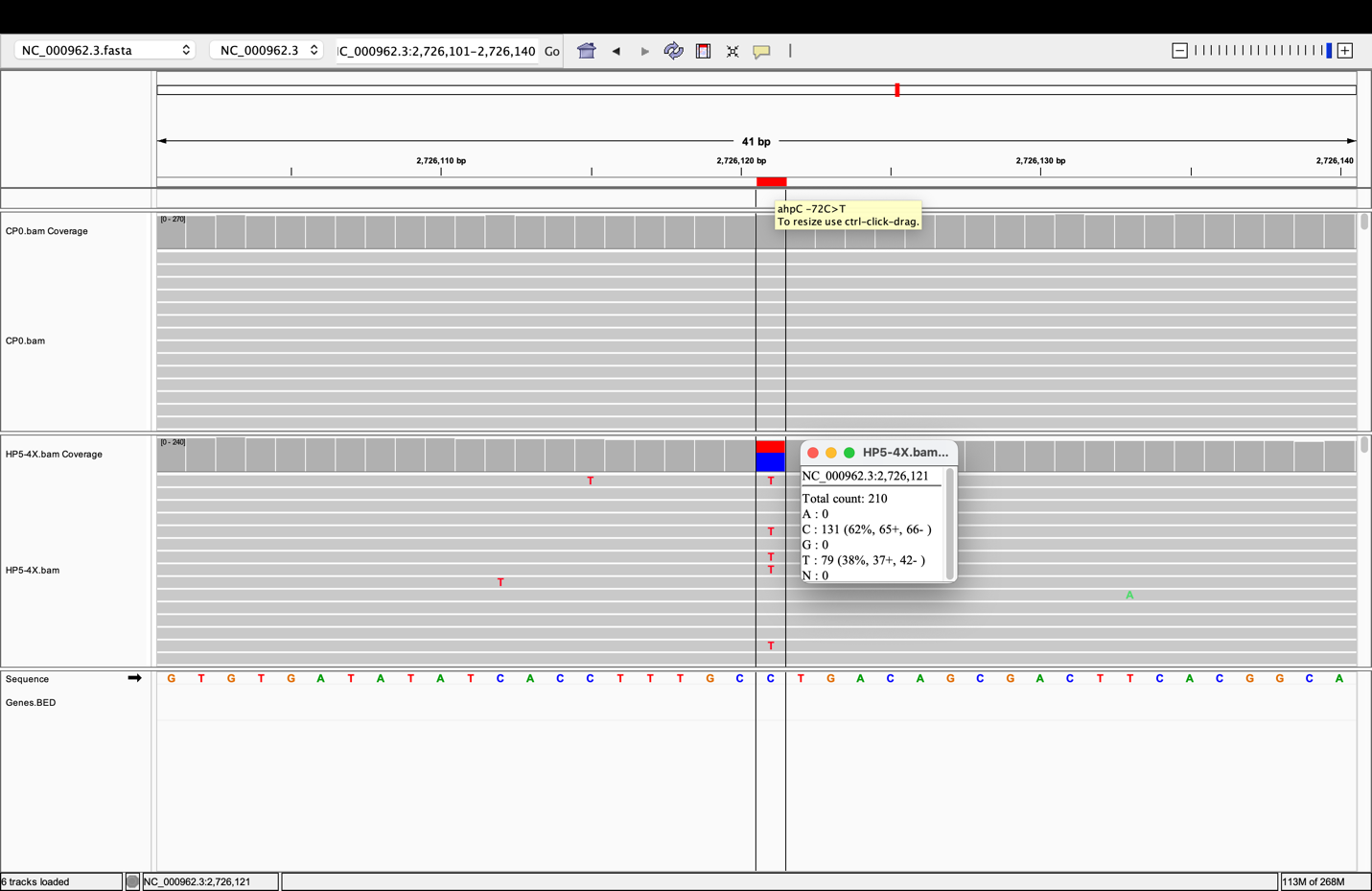

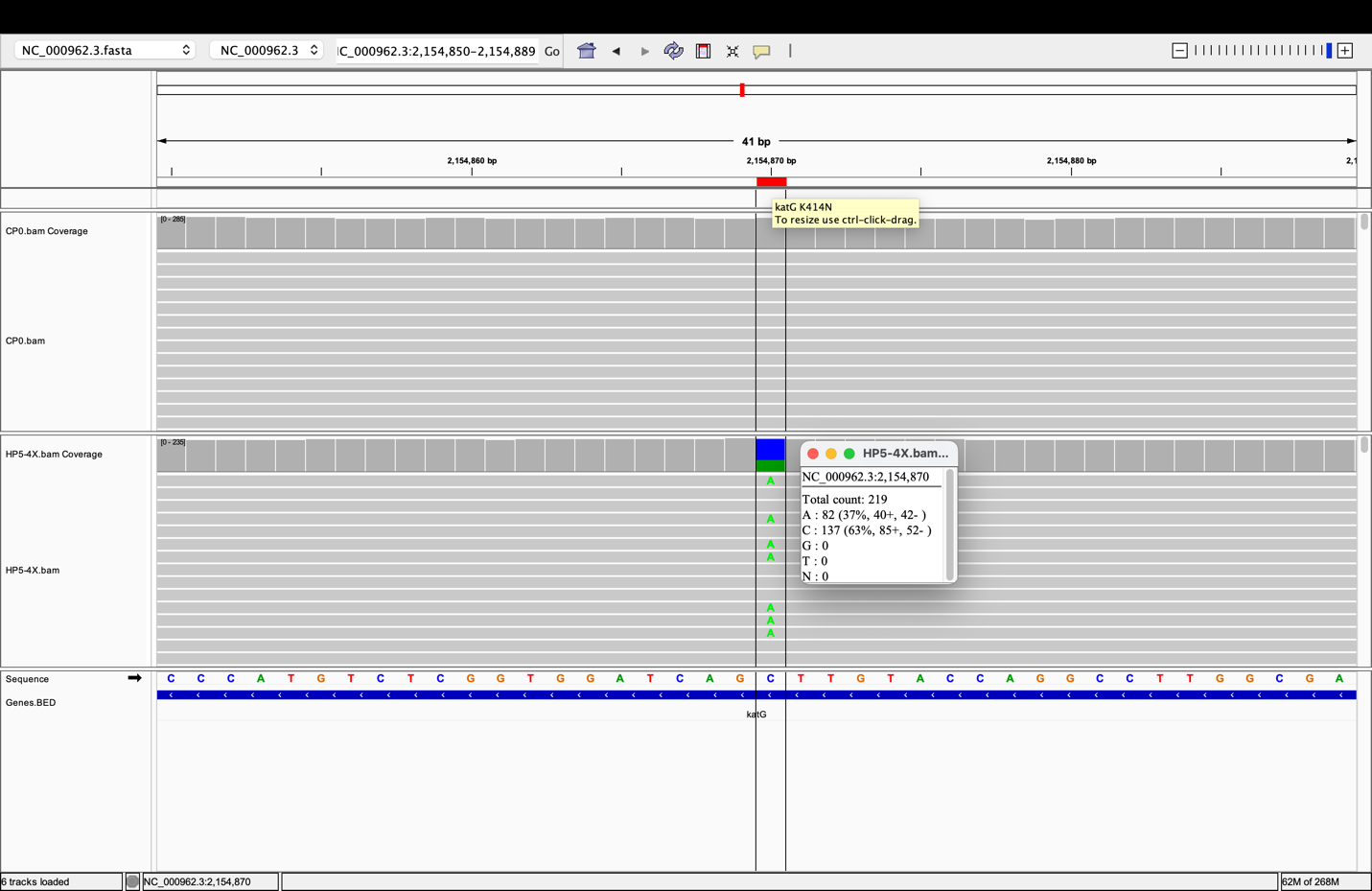


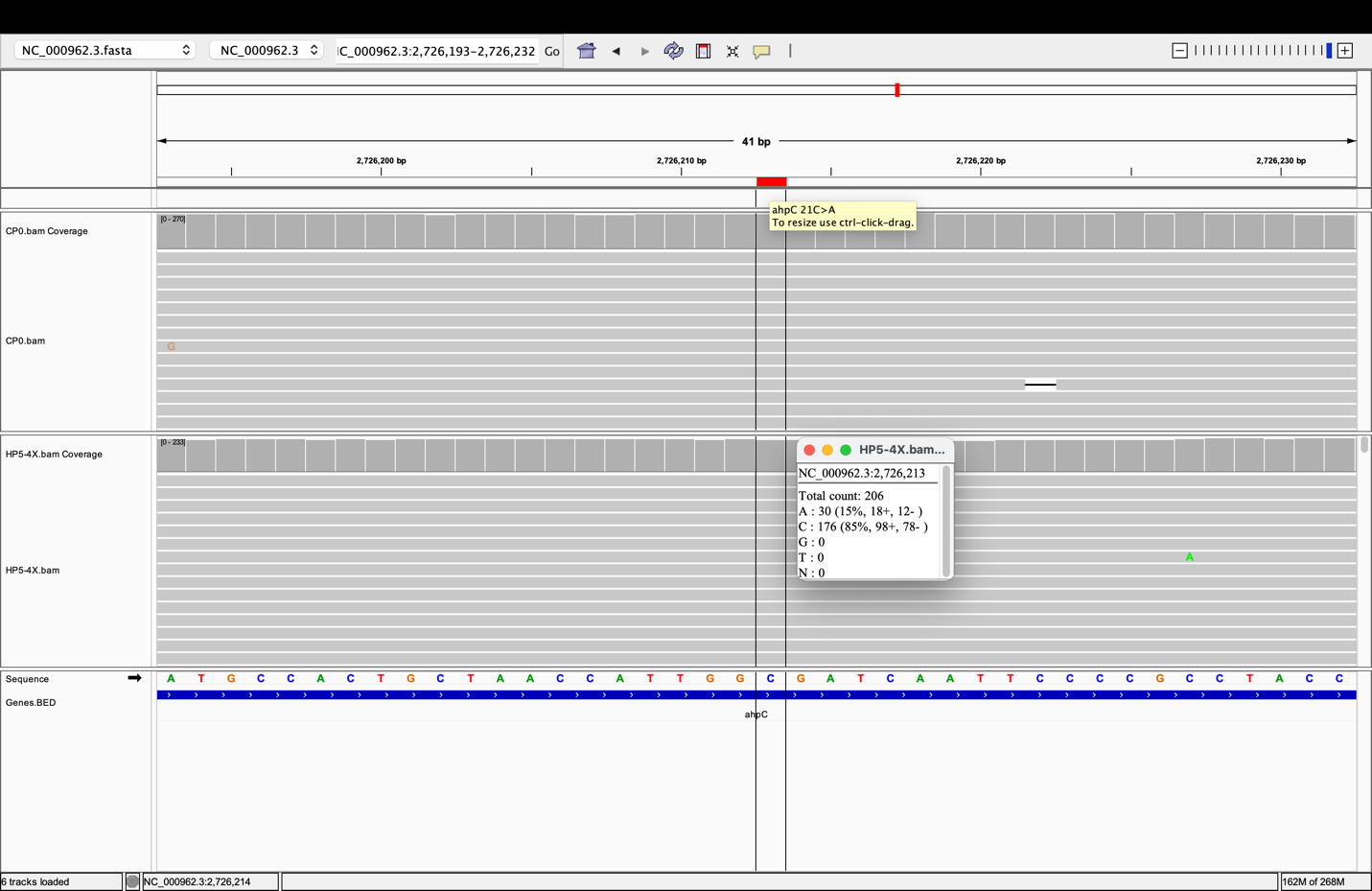

Supplement: Supplementary file 1 [file Data_Sheet_1.docx]
